# Rutin-encapsulated manganese carbonate nanoparticles for ‘liver-dark/tumor-bright’ magnetic resonance imaging and inhibition of breast cancer with liver metastases

**DOI:** 10.1093/rb/rbag049

**Published:** 2026-03-09

**Authors:** Lingfeng Zhao, Yuanyuan Gao, Tao Chen, Yongxi Li, Aijia Chen, Zhengju Hou, Yu Pu, Wenxue Li, Xinghui Li, Xiaoming Zhang, Changqiang Wu

**Affiliations:** Medical Imaging Key Laboratory of Sichuan Province and School of Medical Imaging, North Sichuan Medical College, Nanchong 637000, China; Medical Imaging Key Laboratory of Sichuan Province and School of Medical Imaging, North Sichuan Medical College, Nanchong 637000, China; Medical Imaging Key Laboratory of Sichuan Province and School of Medical Imaging, North Sichuan Medical College, Nanchong 637000, China; Medical Imaging Key Laboratory of Sichuan Province and School of Medical Imaging, North Sichuan Medical College, Nanchong 637000, China; Medical Imaging Key Laboratory of Sichuan Province and School of Medical Imaging, North Sichuan Medical College, Nanchong 637000, China; Medical Imaging Key Laboratory of Sichuan Province and School of Medical Imaging, North Sichuan Medical College, Nanchong 637000, China; Medical Imaging Key Laboratory of Sichuan Province and School of Medical Imaging, North Sichuan Medical College, Nanchong 637000, China; Department of Radiology, Affiliated Hospital of North Sichuan Medical College, Nanchong, Sichuan 637000, China; Medical Imaging Key Laboratory of Sichuan Province and School of Medical Imaging, North Sichuan Medical College, Nanchong 637000, China; Medical Imaging Key Laboratory of Sichuan Province and School of Medical Imaging, North Sichuan Medical College, Nanchong 637000, China; Department of Radiology, Affiliated Hospital of North Sichuan Medical College, Nanchong, Sichuan 637000, China; Medical Imaging Key Laboratory of Sichuan Province and School of Medical Imaging, North Sichuan Medical College, Nanchong 637000, China; Department of Radiology, Affiliated Hospital of North Sichuan Medical College, Nanchong, Sichuan 637000, China; Medical Imaging Key Laboratory of Sichuan Province and School of Medical Imaging, North Sichuan Medical College, Nanchong 637000, China

**Keywords:** rutin, manganous carbonate, breast cancer liver metastasis, magnetic resonance imaging, theranostics

## Abstract

Patients with breast cancer liver metastasis (BCLM) have a low survival rate and poor prognosis; thus, early and precise diagnosis and treatment are important. Here, we developed rutin-encapsulated manganese carbonate nanoparticles (RM NPs) for sensitive magnetic resonance imaging (MRI) diagnosis and effective inhibition of BCLM. RM NPs are nanoclusters consisting of manganese carbonate nanocrystals. They have pH-responsive properties and release Mn^2+^ following their uptake into cells, generating MRI signals. Using a 4T1 mouse liver metastasis model, we demonstrated different uptake rates of RM NPs between normal liver tissue and metastatic tumors. The ‘liver-dark/tumor-bright’ (tumor-to-normal liver contrast ratio reached 219%) phenomenon can be produced using a specific T_1_WI imaging sequence, which enables precise imaging of submillimeter (less than 1 mm) BCLM. *In vitro* and *in vivo* experiments confirmed that because of the rutin sugar groups on the surface of RM NPs, they actively target tumor cells with overexpressed glucose transporters (Gluts). After being taken up by tumor cells, RM NPs release rutin, which induces tumor cell apoptosis by upregulating cleaved cysteine protease-3 and thereby inhibiting tumor cell growth and liver metastasis. Overall, RM NPs can serve as an effective and safe theranostic platform for precise MRI and treatment of BCLM.

## Graphical Abstract

**Graphical Abstract:**
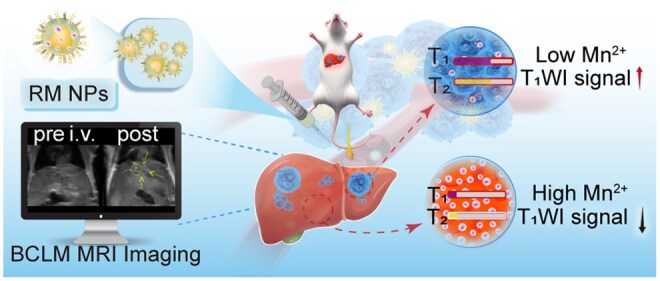

## Introduction

Because of its abundant blood supply and sinusoidal cytoarchitecture, which promote the rapid invasion of circulating cancer cells, liver metastasis (LM) frequently occurs in many cancers [1]. Approximately 20% of patients with breast cancer experience a recurrence, with liver metastases (BCLM) occurring in 50–70% of the metastatic cases [2]. The median survival of BCLM patients is only 3–15 months [3], which highlights the importance of precise diagnosis during the therapeutic window. Currently, the clinical diagnosis of liver metastases primarily relies on magnetic resonance imaging (MRI), computed tomography (CT) and ultrasonography [4], with contrast-enhanced MRI exhibiting high sensitivity [5]; however, gadolinium-based contrast agents (GBCAs), such as Magnevist and Primovist, can cause nephrogenic systemic fibrosis in patients with renal insufficiency, leading to their restrictions by regulatory agencies [6–8]. Moreover, Primovist exhibits limited sensitivity (45–80%) for detecting 1–2 cm liver metastases and even lower sensitivity for lesions <1 cm [9]. This highlights the need for alternatives to Gd to achieve the precise diagnosis of small BCLM lesions.

To overcome the limitations of GBCAs, multifunctional nanoparticle-based MRI contrast agents have been developed as theranostic nanomedicines [10]. Various inorganic NPs, such as noble metal NPs, quantum dots, magnetic iron oxide NPs and magnetic/optical rare-earth NPs [11], have been developed and studied [12]. However, most NPs exhibit ‘always-on’ MRI signals regardless of their interaction with cancer biomarkers [13, 14], making imaging outcome dependent on their biodistribution, particularly in vascularized tissues. Because of the highly vascular nature of the liver, developing activatable NPs that respond to tumor-specific pathological parameters is important for accurate BCLM diagnosis while minimizing vascular interference. Such nanoprobes are typically designed to respond to the characteristics of the tumor microenvironment, such as pH [15–17], redox potential [18–21] and specific enzyme expression [22–25], to enhance diagnostic sensitivity and specificity.

Manganese (Mn^2+^)-based responsive nanomaterials exhibit distinct advantages for addressing these challenges. As an essential micronutrient, Mn^2+^ plays an important role in various cellular and physiological processes [26, 27]. The released Mn^2+^ can enhance T_1_-weighted imaging (T_1_WI) signals, with further signal amplification following protein binding. For example, manganese oxide (MnO_*x*_) nanomaterials can release Mn^2+^ into acidic environments, significantly improving T_1_ relaxivity, whereas nonspecific protein binding of the released Mn^2+^ further enhances contrast [28, 29]. However, most MnO_*x*_ materials release Mn^2+^ only under relatively acidic conditions (pH 4–6) and sometimes require glutathione (GSH) for assistance [30]. This limits their activation in response to intracellular acidic conditions rather than the tumor microenvironment. Considering the kinetics of internalization, transmembrane pathways and cellular distribution of MnO_*x*_ NPs, the development of activatable MRI probes that can rapidly and accurately respond to tumor microenvironmental factors is necessary [31].

To achieve microenvironment-responsive targeting, exploiting the metabolic vulnerabilities of a tumor offers a strategic solution. One feature of malignant tumors is their dependence on inefficient glycolysis for energy production (Warburg effect) [32]. This results in elevated glucose metabolism compared with normal cells [33]. Glucose transporters (Gluts), which are often overexpressed in malignant cells, represent promising targets for cancers, including breast cancer [34]. Glucose and its derivatives may be exploited as tumor-targeting moieties for designing nanomedicines. Rutin, a natural flavonoid containing glucosyl–rhamnosyl disaccharide groups and derived from fruits and vegetables, exhibits multiple biological activities, including cytoprotective, vasculoprotective, antioxidant and anticancer effects [35, 36], making it an ideal natural molecule for breast cancer inhibition.

**Figure 1:**
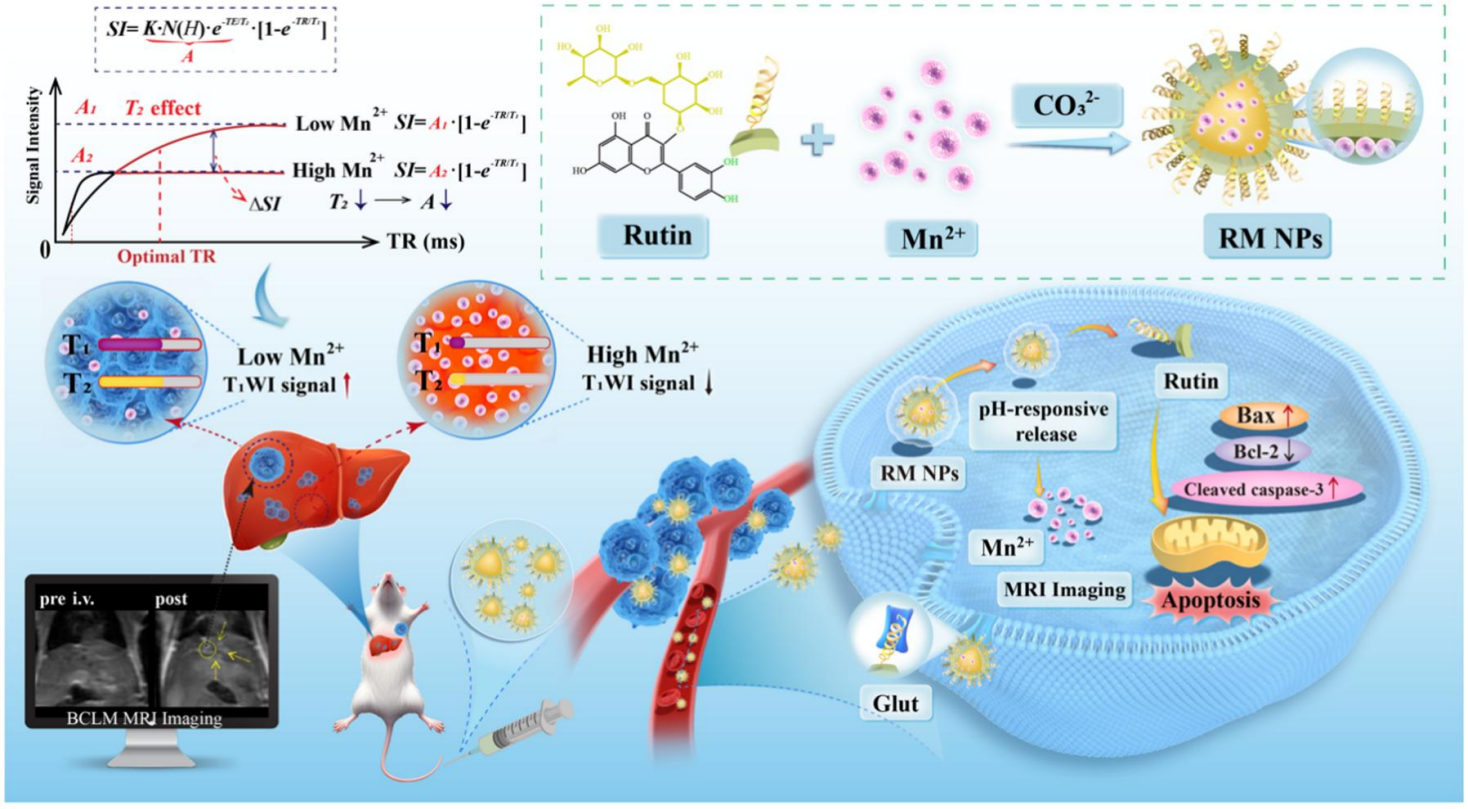
Diagram illustrating the synthesis of RM NPs and the mechanisms of MRI imaging and tumor suppression within BCLM.

In the present study, we developed a novel theranostic Mn^2+^-based responsive nanomaterial, rutin-coated manganese carbonate NPs (RM NPs), using a one-step mineralization approach (Figure 1). These NPs were synthesized by chelating Mn^2+^ with the catechol groups of rutin, followed by ${\mathrm{CO}}_{3}^{2-}$ mineralization. Following intravenous administration, these RM NPs respond to the mildly acidic tumor milieu by releasing Mn^2+^ for T_1_WI. This enables imaging of primary breast tumors and provides unique advantages for BCLM detection in the highly vascularized liver, because RM NPs exhibit minimal T_1_WI signals at a neutral pH. In normal hepatocytes and Kupffer cells, the faster absorption and greater release of Mn^2+^ induce strong T_2_ shortening effects [37] and reduce liver background signals. In contrast, tumor cells take up moderate RM NPs and exhibit T_1_ hyperintensity, thereby creating a ‘dark liver/bright tumor’ contrast pattern that improves the diagnostic specificity for BCLM. The released rutin molecules modulate the expression of the mitochondrial apoptosis-related proteins Bax and Bcl-2 [38] and upregulate cleaved Caspase-3 to promote breast cancer cell apoptosis [39]. These nanoprobe features were studied through a series of *in vitro* and *in vivo* experiments. Overall, we provide a novel design integrating MRI capacity and therapeutic activity in a nanoprobe for BCLM by exploiting aberrant cancer metabolism.

## Materials and methods

### Synthesis of RM NPs

To synthesize RM NPs, 150 mg of rutin (10 mg/mL) was dissolved in 10 mL of dimethyl sulfoxide (DMSO) in a 50 mL round-bottom flask. Next, 50 mg of MnCl_2_·4H_2_O was added under constant stirring for 1 h. Subsequently, 5 mL of sodium carbonate solution (200 mM) was added during sonication. After 5 min of sonication, when the solution color changed from light green to dark green, the mixture was continuously stirred at room temperature for 4 h. The resulting product was collected via centrifugation at 8000 rpm for 5 min and washed once with ethanol and twice with water. The resulting RM NPs were dissolved in 5 mL of ultrapure water and stored at 4°C. Mn concentration was quantified using atomic absorption spectroscopy (AAS) following nitric acid digestion.

### *In vitro* targeting

4T1 and RAW 264.7 cells were seeded into 6-well plates. After these cells reached the logarithmic growth phase, the experiments were commenced. Both cell types were treated identically. The cells were divided into two groups (*n* = 12 wells total). One group received PBS containing 20% (w/v) glucose, whereas the other received only PBS. Each group contained RM NPs at Mn concentrations of 10, 30, 50 and 80 µg/mL (*n* = 3 per concentration). After a 2 h coincubation, the supernatant was removed, and the cells were washed three times with PBS to remove residual NPs, trypsinized, counted and pelleted. The cells (1 × 10^6^) were immobilized in 1 mL of 8% (w/v) agarose. The T_1_ relaxation times for the fixed cell pellets were measured at 3.0 T, and T_1_-mapping images were generated using MATLAB software. Aliquots of the cell suspension were digested overnight with 75% nitric acid. After dilution, the intracellular Mn concentration was quantified using AAS, and the mass of Mn uptake per 1 × 10^6^ cells was calculated. T_1_ relaxation times at 3.0 T were acquired using a conventional IR-Fast Spin Echo (FSE) sequence using the following parameters: TE = 16 ms, TR = 3000 ms, TI = 50–1500 ms, slice thickness = 3 mm, FOV = 180 mm, ETL = 24 and flip angle = 125°.

### Examining the T_2_ shortening effect

RM NPs were prepared at manganese concentrations ranging from 0 to 0.5 mM in PBS (pH 6.6), with three replicates for each concentration. To establish consistency with the MRI sequence used for BCLM, an FSE sequence was used to measure the T_2_ shortening effect. The parameters were as follows: TE = 20 ms (fixed), TR = 100–4000 ms, ETL = 3, slice thickness = 2 mm and FA = 90°. MRI was performed while varying the TR values. A blank control group consisting of PBS (pH 6.6) was also included. After acquiring the MRI images, the signal intensity of each manganese concentration of RM NPs (*SI*) was measured using DICOM analysis software. The corresponding signal intensity of the blank control (*SI*_0_) was also measured. The following formula was used:


$$\begin{array}{c} \text{Relative}\;\text{Signal}\;\text{Intensity}\;\left(\mathrm{Relative}\;SI\right)=SI/SI_{0}\\ \text{Signal}\;\text{Intensity}\;\left(SI\right)=K\cdot N\;\left(H\right)\cdot \underset{T_{2}\text{WI}\;\text{effect}}{\underset{︸}{e^{-TE/T_{2}}}}\cdot [\underset{T_{1}\text{WI}\;\text{effect}}{\underset{︸}{1-e^{-TR/T_{1}}}}]\\ \frac{1}{T_{1}}=\frac{1}{T_{1m}}+r_{1}C\;\;\;\frac{1}{T_{2}}=\frac{1}{T_{2m}}+r_{2}C \end{array}$$

*SI*: signal intensity; *K*: constant; *N(H)*: proton density; *e*: base of the natural logarithm; *TE*: echo time; *TR*: repetition time; *T_1m_* and *T_2m_*: longitudinal and transverse relaxation time of the matrix without contrast agent, respectively; *r*_1_ and *r*_2_: longitudinal and transverse relaxivities of the contrast agent, respectively; *C*: concentration of the contrast agent.

### *In vivo* experiments

#### In vivo targeting and biodistribution analysis

4T1 cells (5 × 10^6^) were implanted subcutaneously into the left axilla of female BALB/c mice (18–22 g). When the tumors reached approximately 50 mm^3^, the mice were fasted for 24 h. Twelve mice were divided into two groups (*n* = 3 per time point per group). Group I received RM NPs via tail vein injection (0.1 mmol Mn/kg body weight). Group II initially received a 20% glucose solution via oral gavage (10 mL/kg). Blood glucose levels were monitored using a portable glucometer. Blood samples were collected from the retro-orbital venous plexus at various time points within a 120 min period post-gavage (specifically at 0, 15, 20, 25, 30, 35, 40, 50, 60 and 120 min) to determine the peak glucose time and ensure effective competitive inhibition of Gluts. The results showed that blood glucose peaked at 15–20 min after gavage (approximately 25–35 mmol/L) and gradually declined, returning to baseline levels by 120 min (Supplementary Figure S1). At 15–20 min post-gavage (during the glucose peak), RM NPs were injected intravenously (0.1 mmol Mn/kg). At 1, 2, 3 and 6 h post injection, three mice per group per time point were euthanized. The liver, spleen, kidney and tumor were harvested from group I. Only tumors were harvested from group II. The tissues were washed with PBS, digested in 75% nitric acid for 7 days, diluted and the Mn concentration was quantified using inductively coupled plasma mass spectrometry (ICP-MS). Three control mice injected with saline were subjected to the same digestion/dilution procedure. Baseline Mn levels in the liver, spleen, kidney and tumor were measured. The net Mn content in the major organs from RM NP-injected mice was obtained by subtracting the corresponding values from the saline-injected controls. All animal procedures were approved by the Animal Ethics Committee of North Sichuan Medical College (Approval No. 2025067).

#### MRI of BCLM

Liver metastases were established by injecting 1 × 10^6^ 4T1 cells into the spleens of female BALB/c (6–8 weeks old, 18–22 g) and allowing metastasis formation for 4–5 days. The mice were divided into two groups (*n* = 3 per group). Group I was administered RMNPs. Group II received Primovist^®^ (gadoxetate disodium, a clinically used hepatobiliary-specific contrast agent; injection doses: 0.1 mmol/kg Mn and 0.1 mmol/kg Gd, respectively). Precontrast images were acquired, followed by postcontrast imaging at 0.5, 1, 2 and 3 h. Following imaging, the mice were euthanized and dissected, and metastatic foci corresponding to the MRI locations were photographed and processed for hematoxylin and eosin (H&E) staining. The signal characteristics of metastases at different times were analysed using DICOM Viewer software. The signal intensity within the regions of interest was analysed to determine the advantages of RM NPs over Primovist^®^ for LM imaging. LM imaging at 3.0 T (GE scanner) was performed using a T_1_-weighted rapid acquisition with relaxation enhancement (RARE) sequence with the following parameters: *TE *= 17 ms, *TR *= 480 ms, slice thickness = 0.4 mm, spacing between slices = 0.8 mm, *ETL *= 3, *FOV *= 100 mm, number of averages = 2 and flip angle = 142°.

#### Therapeutic evaluation of subcutaneous orthotopic breast tumors

4T1 cells (5 × 10^6^) were implanted subcutaneously into the left axilla of female BALB/c (6–8 weeks old, 18–22 g). After 5 days, when tumors reached 50–80 mm^3^, treatment was commenced. The mice were randomly divided into three groups (*n* = 4 per group). On days 0, 3 and 6, the groups were intravenously administered PBS, low-dose RM NPs (0.05 mmol Mn/kg), and high-dose RM NPs (0.1 mmol Mn/kg). Tumor size and body weight were recorded every 2 days for 14 days. On the final day, the mice were euthanized, and the tumors were excised, photographed, weighed and processed for H&E staining. Tumor volume (*V*) was calculated as follows:


$$V=({\text{width}}^{2}\times \text{length})/2.$$

#### Therapeutic evaluation of BCLM

Liver metastases were established by splenic injection of 1 × 10^6^ 4T1 cells. The mice were randomly divided into three groups (*n* = 4 per group). Four days post-implantation, they received intravenous injections of PBS, low-dose RM NPs (0.05 mmol Mn/kg) or high-dose RM NPs (0.1 mmol Mn/kg). On day 8, an MRI was conducted to obtain pre- and postcontrast abdominal images at 2 h following the injection of the RM NPs (0.1 mmol Mn/kg group). Subsequently, the mice were euthanized and dissected. Metastatic foci were photographed, livers were excised, photographed and sections from the median liver lobe of each group were processed for H&E staining. To assess therapeutic efficacy, after metastasis establishment, the mice were randomly divided into two groups (*n* = 3 per group). On days 4 and 8 post-splenic injection, the groups received PBS or RM NPs (0.05 mmol Mn/kg). On day 12, the mice received RM NPs (0.1 mmol Mn/kg) for LM imaging, acquiring precontrast and 2 h postcontrast (RMNPs, 0.1 mmol Mn/kg) abdominal images. Finally, the abdominal cavity of each mouse was photographed. The mice were euthanized and dissected, and liver metastatic lesions were photographed. The livers were weighed, and sections were processed for H&E staining.

## Results and discussion

### Preparation and characterization of RM NPs

Mn^2+^ carbonate NPs (MnCO_3_ NPs) can be prepared at room temperature using a coprecipitation method; however, their size is hard to control as micron-scale particle growth occurs [40]. Conventional MnCO_3_ NPs are typically synthesized via microemulsion methods [41, 42]. However, surface residual cytotoxic cetyltrimethylammonium bromide requires additional modifications to enhance biocompatibility, which complicates the synthesis process. In the present study, RM NPs were synthesized using a one-step mineralization approach under mild conditions [40, 43], in which the phenolic hydroxyl groups of rutin coordinated with manganese carbonate. To determine the optimal rutin concentration for RM NP synthesis, the NPs were prepared using a range of rutin ligand concentrations (0, 0.3, 0.7, 3.3, 6.7 and 10 mg/mL). Increasing rutin concentration resulted in a smaller nanoparticle size, a more defined morphology and improved dispersibility, as observed via transmission electron microscope (TEM) (Supplementary Figure S2A). Similarly, dynamic light scattering (DLS) revealed a gradual decrease in hydrodynamic diameter and PDI with increasing rutin concentration (Supplementary Figure S2B). Overall, a rutin concentration of 10 mg/mL was selected for further characterization to confirm successful RM NP preparation.

**Figure 2:**
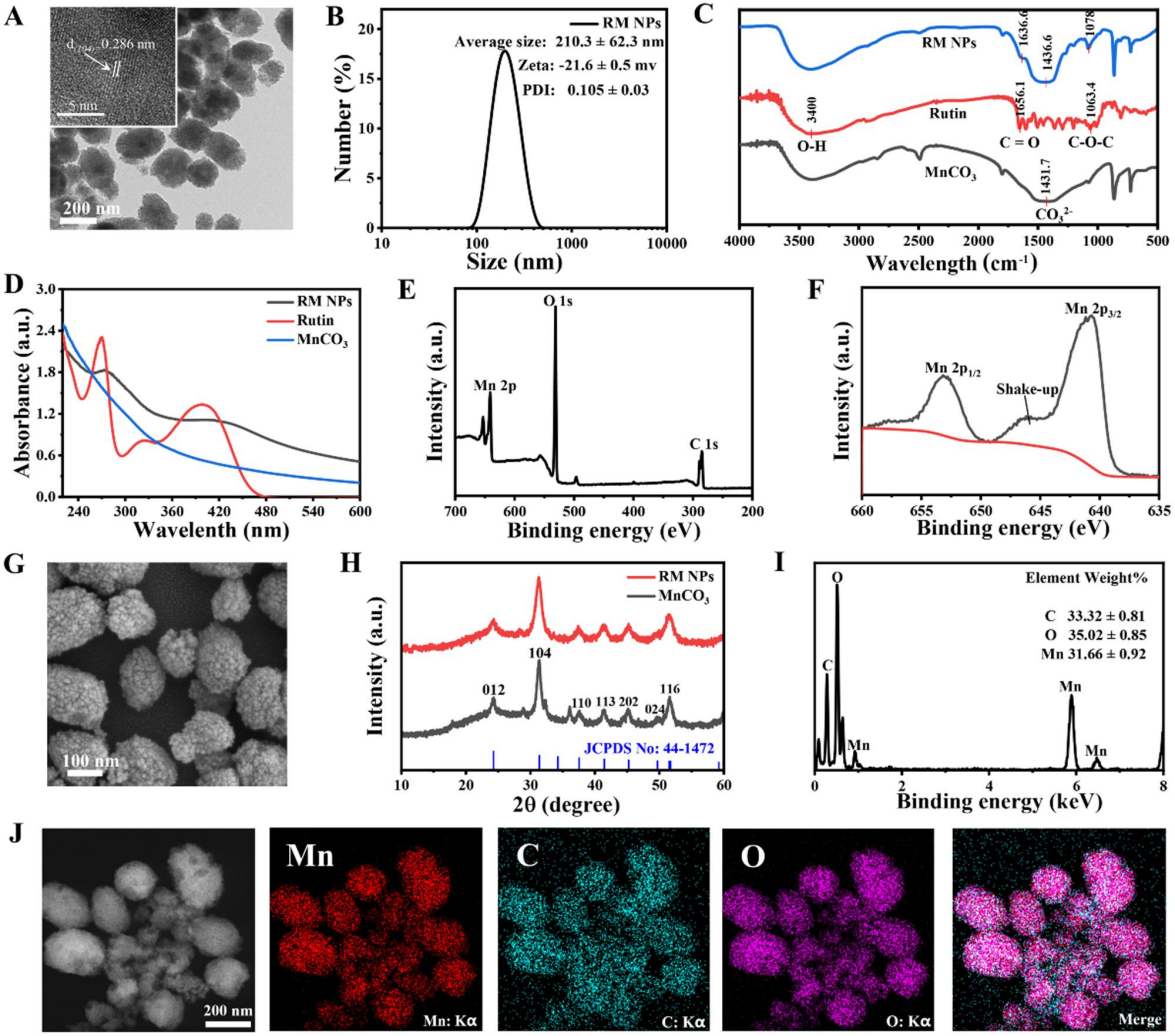
Synthesis and preparation of RM NPs. (**A**) TEM characterization of the morphology of RM NPs, and HRTEM shows their lattice spacing. (**B**) DLS characterizing the hydrodynamic diameter, aqueous dispersity and zeta potential of RM NPs. (**C**) FTIR spectroscopy showing the functional groups contained in RM NPs. (**D**) UV–vis spectroscopy showing the absorption peak corresponding to the rutin component in RM NPs. (**E**, **F**) XPS showing the elemental composition of RM NPs and the valence state of Mn^2+^. (**G**) SEM showing the 3D morphology of RM NPs. (**H**) XRD showing the crystal structure of RM NPs. (**I**, **J**) EDS spectrum and elemental mapping revealing the elemental composition ratios and distribution of RM NPs.

DLS analysis revealed a hydrodynamic diameter of 210.3 ± 62.3 nm and a PDI of 0.105 ± 0.03 for the optimized RM NPs. NPs ranging from 50 to 200 nm in size are efficiently taken up by the liver (60–90%) [44], thereby providing a foundation for subsequent MRI and treatment of BCLM. The low PDI further confirms the excellent dispersibility of RM NPs. An appropriately negative zeta potential (Figure 2B) minimizes nonspecific uptake during systemic circulation [45]. TEM visually confirmed the near-spherical morphology and size of RM NPs. High-resolution TEM (HRTEM) revealed that RM NPs are clusters consisting of MnCO_3_ nanocrystals, with a lattice stripe spacing of 0.286 nm (Figure 2A). This corresponds to a 104 crystal plane of MnCO_3_. X-ray diffraction (XRD) analysis further corroborated the crystal structure of RM NPs, which showed consistency with synthesized MnCO_3_ NPs and a standard MnCO_3_ pattern (JCPDS No: 44-1472) (Figure 2H). Scanning electron microscopy (SEM) revealed additional insight into the 3D structure of RM NPs (Figure 2G).

Fourier transform infrared (FTIR) spectroscopy confirmed the presence of CO_3_ groups in RM NPs, with a characteristic peak at 1436.6 cm^−1^, which aligned with the peak at 1431.7 cm^−1^ in the synthesized MnCO_3_ NPs. Additional peaks at 1636.6 and 1078 cm^−1^ corresponded to the C=O and C–O–C bonds of the rutin ligand, respectively (Figure 2C). Rutin typically displays two UV absorption bands: band I (300–400 nm) and band II (220–280 nm), which are attributed to π–π* transitions of aromatic rings and n–π* transitions of hydroxyl substituents, respectively [46]. Ultraviolet spectrum–vis spectroscopy of RM NPs showed absorption peaks near 400 and 280 nm, which are consistent with rutin. The overall increased baseline intensity of RM NPs compared with pure rutin is attributed to the successful formation of MnCO_3_ (Figure 2D). XPS indicated the presence of manganese (Mn), carbon (C) and oxygen (O) in RM NPs. Deconvolution of the Mn 2p spectrum revealed the characteristic shakeup satellite peak of Mn^2+^, and the binding energy of the Mn 2p_3/2_ main peak (640–642 eV) confirmed the Mn^2+^ oxidation state (Figure 2E and F) [47]. EDS mapping further verified the exclusive presence of C, O and Mn in RM NPs, which was consistent with the XPS results. Elemental quantification via EDS revealed an approximate rutin-to-MnCO_3_ ratio of 4:6 (Figure 2I and J), which was corroborated by AAS measurements (MnCO_3_: 60.34%, rutin: 39.66%). Overall, these results confirm the successful synthesis of rutin-wrapped MnCO_3_ NPs.

### Evaluation of pH-responsive properties and relaxivity performance of RM NPs

**Figure 3:**
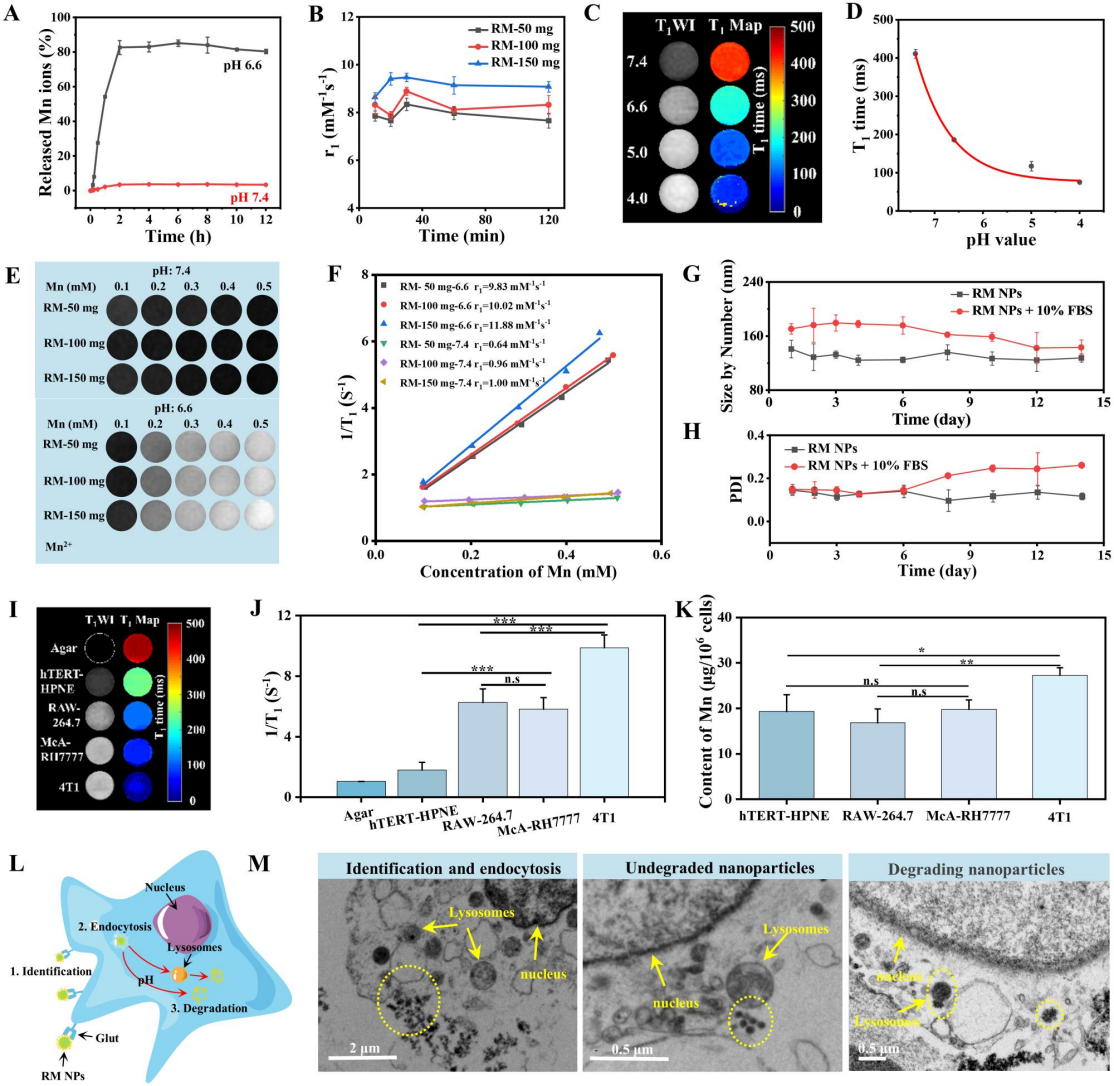
Evaluation of the release characteristics and pH response of RM NPs. (**A**) Time-dependent Mn^2+^ release properties of RM NPs at pH 6.6. (**B**) Time-dependent T_1_ relaxation rate of RM NPs in a slightly acidic environment (pH 6.6) at 0.5 T. (**C, D**) The T_1_ value of RM NPs solution at different pH values. (**E, F**) The T_1_ relaxation rate of RM NPs and free Mn^2+^ at 3.0 T in neutral (pH 7.4) and slightly acidic (pH 6.6) conditions. (**G, H**) Stability of RM NPs dispersed in water and 10% serum at 14 days. (**I, J**) T_1_ value of cell gel for hTERT-HPNE, RAW 264.7, McA-RH7777 and 4T1 incubated with RM NPs for 2 h, and (**K**) amount of Mn^2+^ in each cell. (**L**) Postuptake schematic of RM NPs in 4T1 cells. (**M**) Bio-TEM showing the recognition, internalization and degradation of RM NPs in 4T1 cells. Statistical significance was calculated using one-way analysis of variance (ANOVA). *P < 0.05, **P < 0.01, ***P < 0.001 vs. control, n.s: no significance. Data are expressed as mean ± SD (*n* = 3).

To examine the pH-dependent Mn^2+^ release capability of RM NPs, we simulated the tumor microenvironment (mildly acidic, pH 6.4–6.8) and normal physiological conditions (blood, pH 7.35–7.45) *in vitro*. RM NPs rapidly released approximately 80% of their Mn^2+^ content in PBS at pH 6.6 within 2 h, and were stabilized thereafter. In contrast, minimal Mn^2+^ release was observed at pH 7.4 (Figure 3A). TEM analysis corroborated these findings. RM NPs exhibited significant degradation after 2 h at pH 6.6 but were largely intact at pH 7.4 (Supplementary Figure S3A and B). These results confirm the pH-responsive degradation and Mn^2+^ release capability of RM NPs under mildly acidic conditions.

To further characterize the pH-responsive properties *in vitro*, we first monitored the longitudinal relaxivity (*r*_1_) of RM NPs prepared with a range of rutin ligand concentrations (3.3, 6.7, 10 mg/mL, corresponding to RM-50 mg, RM-100 mg and RM-150 mg) in pH 6.6 buffer for various times (10, 20, 30, 60 and 120 min). The *r*_1_ value peaked at approximately 30 min and subsequently stabilized. Notably, RM-150 mg exhibited significantly higher *r*_1_ compared with RM-50 mg and RM-100 mg (Figure 3B), which confirms the time-dependent relaxivity enhancement under acidic pH. This phenomenon may be attributed to RM-150 mg exhibiting a more polycrystalline structure, and their porous nature allows more water molecules to readily penetrate and reach the crystal surface to interact with the released Mn^2+^ to enhance inner-sphere relaxation. The stabilization of *r*_1_ after ∼30 min corresponds to the transition of Mn^2+^ release from a rapid initial phase to a plateau. The apparent temporal discrepancy between the Mn^2+^ release profile (Figure 3A, plateau at ∼2 h) and the *r*_1_ kinetics (Figure 3B, plateau at ∼30 min) arises from the distinct methodologies employed. Figure 3A quantified the cumulative Mn^2+^ concentration in the dialysate using a dialysis method, where diffusion across the membrane is rate-limiting, resulting in a slower measured release profile. In contrast, Figure 3B directly and in real-time measured the relaxivity change of the nanoparticle dispersion, which is more sensitive to the immediate state of Mn^2+^ release and its interaction with water molecules. Therefore, these two experiments serve complementary purposes—validating the total release amount versus characterizing the pH-activated relaxivity response—and are not contradictory in mechanism.

Second, RM NPs (Mn concentration, 0.3 mM) were dispersed in buffers of different pH (7.4, 6.6, 5.0 and 4.0). T_1_ mapping revealed progressively brighter signals with decreasing pH (Figure 3C). The measured 1/T_1_ showed a clear pH-dependent increase (Figure 3D). This phenomenon is attributed to the increased release of Mn^2+^ ions during the transition from bound to free states at a lower pH, whereas highly acidic conditions promote the structural degradation of RM NPs, thereby exposing additional manganese sites. Overall, these data provide robust evidence for the pH-responsive nature of RM NPs.

The T_1_WI performance of the three RM NP formulations (RM-50 mg, RM-100 mg, RM-150 mg) was evaluated at 3.0 T. At pH 7.4, the T_1_WI signal enhancement was minimal with increasing Mn concentrations (0.1–0.5 mM); however, at pH 6.6, the T_1_WI signal intensity increased progressively with Mn concentration. Therefore, the *r*_1_ of the three RM NP was tested under the condition of pH 6.6. The experimental results show that the *r*_1_ increased with higher rutin ligand concentration. All three RM NPs exhibited higher *r*_1_ compared with that of the commercially available Magnevist and Primovist (4.22 and 4.85 mM^−1^ s^−1^) (Figure 3E and F, Supplementary Figure S4 and Table S1). In particular, RM-150 mg achieved an *r*_1_ of 11.88 mM^−1^ s^−1^, which was 1.41-fold higher than that of MnCO_3_-PDA [42]. Similar trends were observed at lower field strengths (0.5 and 1.41 T), with RM-150 mg showing the highest *r*_1_ (Table S1). To eliminate the effect of the buffer, the *r*_1_ and *r*_2_ of Mn^2+^ in neutral aqueous solution were not significantly different compared with those in the pH 6.6 buffer (Supplementary Figure S5). At pH 6.6, the *r*_1_ of RM-150 mg (11.88 mM^−1^ s^−1^) was slightly higher than that of free Mn^2+^ (9.98 mM^−1^ s^−1^). This increase likely resulted from the released Mn^2+^ further chelating with rutin molecules, which increased their rotational correlation time (*τ*_R_), thereby enhancing T_1_ relaxation (Figure 3E and F). All three RM NPs showed higher *r*_2_ compared with free Mn^2+^. This was attributed to a more pronounced decrease in transverse relaxation time resulting from partial aggregation of the RM NPs following their pH 6.6-responsive behavior (Supplementary Figure S6). Moreover, the *r*_2_/*r*_1_ ratio is an important parameter to estimate the efficiency of T_1_ contrast agents (CAs). Of these, the lower *r*_2_/*r*_1_ ratio facilitated a highly efficient T_1_ contrast image. An *r*_2_/*r*_1_ > 8 resulted in a T_2_-dominated MRI CA, whereas an *r*_2_/*r*_1_ < 5 resulted in a T_1_-dominated MRI CA [48]. The *r*_2_/*r*_1_ ratios for all three RM NPs formulations were below 4, and they decreased with increasing rutin concentration (Table S1). This confirmed their suitability as effective T_1_-positive CAs, rather than T_2_-negative CAs.

The stability of RM NPs was determined by dispersing them in water and 10% fetal bovine serum (FBS). Size and PDI were monitored every 2 days for 14 days. The RM NPs maintained relatively stable size and PDI in water throughout the study. In 10% FBS, the stability was maintained during the first 6 days, followed by a gradual decrease in size and an increase in PDI. This change may have arisen from the partial detachment of unstable components following nonspecific chelation with serum proteins over time (Figure 3G and H). Based on its smaller size, superior dispersity, high *r*_1_ (suitability as a T_1_ contrast agent), and good *in vitro* stability, the RM-150 mg formulation (prepared with 10 mg/mL rutin) was selected for further analysis.

### *In vitro* cellular response evaluation

To determine the responsiveness of RM NPs within the tumor microenvironment, we examined two normal cell lines (macrophages RAW 264.7, pancreatic ductal epithelial cells hTERT-HPNE) and two tumor cell lines (rat hepatoma McA-RH7777 cells, mouse breast cancer 4T1 cells). The cells were incubated with RM NPs (Mn concentration: 120 µg/mL) for 2 h, counted to an equal density, immobilized in 8% agarose gel and subjected to T_1_WI at 3.0 T. The T_1_WI signal intensity was highest in the 4T1 cells (Figure 3I). 1/T_1_ values were significantly higher in 4T1 and McA-RH7777 tumor cells than in normal hTERT-HPNE cells (Figure 3J), which was attributed to the mildly acidic cytoplasmic pH (6.4–6.8), which is characteristic of tumor cells that trigger RM NP degradation. The elevated 1/T_1_ value in RAW 264.7 cells likely results from their high phagocytic capacity because of abundant microvilli and pseudopods [49, 50]. Notably, the 1/T_1_ value in 4T1 cells exceeded that in RAW 264.7 cells (Figure 3J), which was corroborated by higher intracellular Mn^2+^ content in 4T1 cells post-lysis (Figure 3K). This may be attributed to the overexpression of Gluts in 4T1 cells, which facilitate the targeted uptake of glucose-functionalized RM NPs. A mechanistic diagram depicting the recognition, internalization and degradation of RM NPs by 4T1 cells is shown in Figure 3L. Bio-TEM imaging directly visualized the recognition, internalization, and degradation of RM NPs within 4T1 cells after a 2-h coincubation (Figure 3M).

### Biosafety assessment of RM NPs

**Figure 4:**
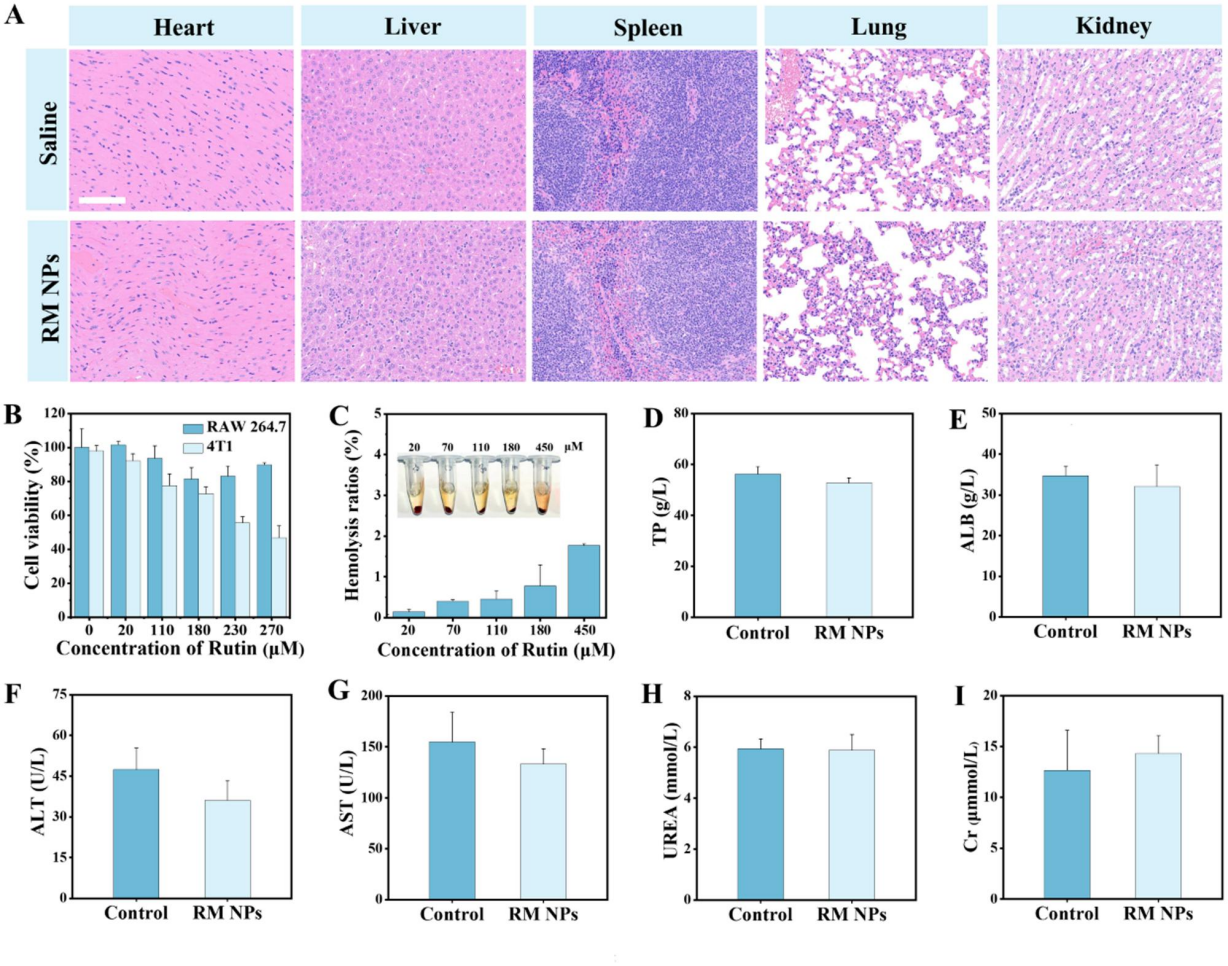
Biosafety assessment of RM NPs. After 10 days of tail vein injection of RM NPs (0.1 mmol/kg Mn), H&E staining of each organ was (**A**) compared with saline. (**B**) CCK8 cytotoxicity assay and (**C**) hemolysis assay (*n* = 4) revealed that RM NPs had a favorable safety profile. A serum biochemical analysis measuring protein content: (**D**) TP and (**E**) ALB; liver function: (**F**) ALT and (**G**) AST and kidney function: (**H**) UREA and (**I**) Cr. All parameters were consistent with those of the saline group (*n* = 3). Scale bar: 100 μm for all images. Data are expressed as mean ± SD.

The biosafety of nanomaterials is important for clinical translation and subsequent applications [51]. Cytotoxicity assays and *in vivo* murine toxicity studies were performed to evaluate the biosafety and biocompatibility of RM NPs before *in vivo* studies. Cytotoxicity was assessed using RAW 264.7 and 4T1 cells. The viability of RAW 264.7 cells remained above 80% across increasing rutin concentrations (0–270 µM) within RM NPs, thereby demonstrating good *in vitro* biocompatibility (Figure 4B). In contrast, 4T1 cell viability decreased progressively with increasing rutin concentration, which was attributed to the inherent anticancer effect of rutin [52]. Moreover, hemolysis assays confirmed the excellent hemocompatibility of RM NPs *in vitro*. Following coincubation with rat red blood cells for 1 h, the hemolysis rate, which was calculated after subtracting the intrinsic absorption of the materials measured at 450 nm, remained below 5% across all RM NPs concentrations tested (Figure 4C).

The *in vivo* biosafety of RM NPs was further evaluated. Female BALB/c were intravenously injected with RM NPs (0.1 mmol Mn/kg). After 10 days, the major organs and serum were collected for biosafety analysis. Serum biochemistry revealed total protein (TP) and albumin (ALB) levels comparable to those of the saline control group (Figure 4D and E). Liver function markers, including alanine aminotransferase (ALT) and aspartate aminotransferase (AST) (Figure 4F and G), and kidney function markers, including blood urea nitrogen (UREA) and creatinine (Cr) (Figure 4H and I), showed no significant increase, indicating an absence of inflammatory damage. Histopathological examination following H&E staining of the major organs (heart, liver, spleen, lungs and kidneys) revealed no substantial pathological lesions (Figure 4A). Taken together, these results indicate the favorable biosafety profile of RM NPs at the tested dose (0.1 mmol Mn/kg) and provide a solid foundation for *in vivo* studies.

### Evaluation of the targeting capability of RM NPs

**Figure 5:**
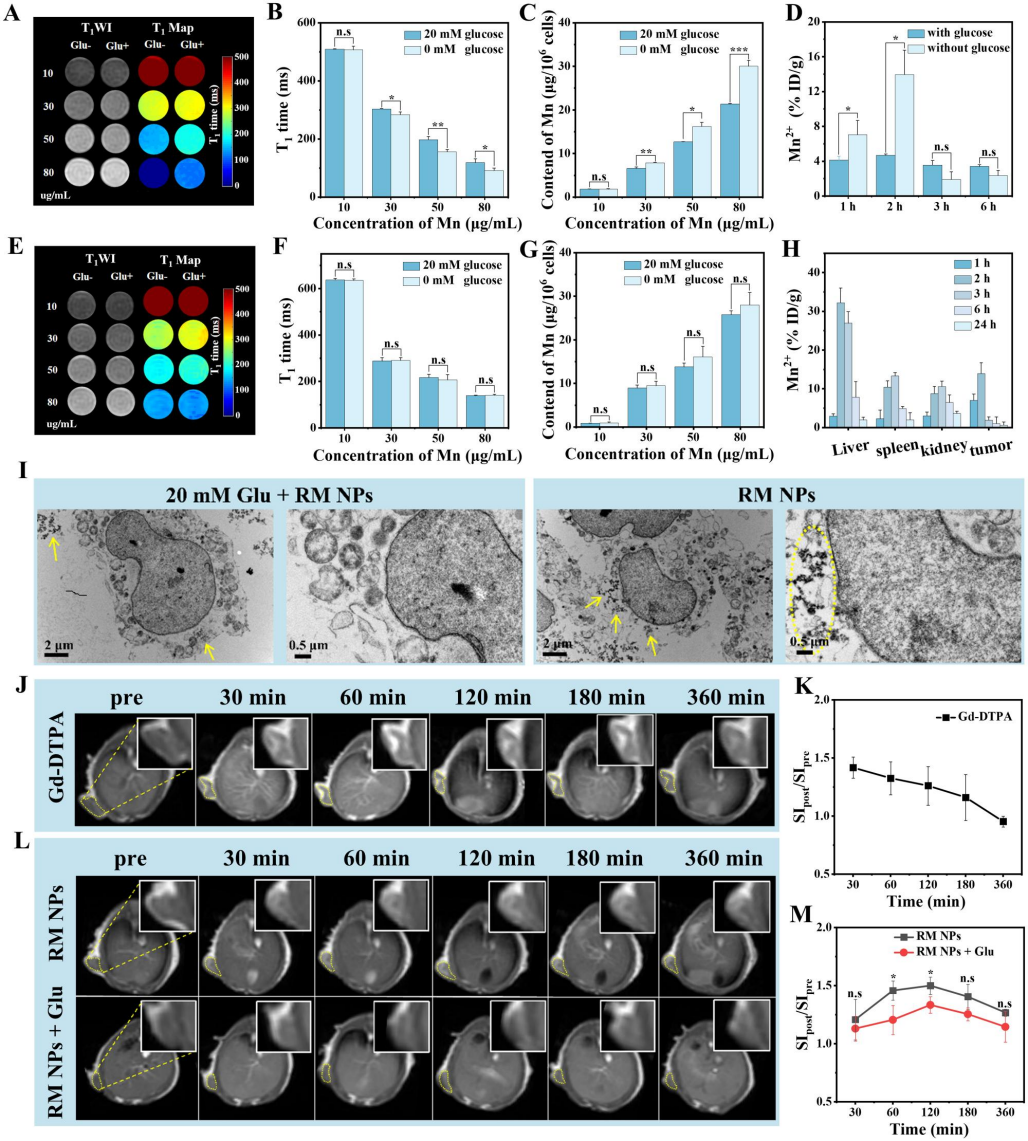
Evaluation of the targeted glucose receptor properties of RM NPs. (**A, B**) T_1_ relaxation times and (**C**) Mn content in 4T1 cells (10^6^) post 2 h incubation with RM NPs. (**D**) Mn^2+^ content (%ID/g) in the tumor and (**H**) major organs of 4T1 tumor-bearing mice at 1, 2, 3 and 6 h post injection. (**E, F**) T_1_ relaxation times and (**G**) Mn content in RAW 264.7 cells following incubation with RM NPs under identical conditions. (**I**) Distinct intracellular distributions of RM NPs in 4T1 cells under 20 mM glucose and glucose-free conditions following a 2 h incubation (arrows indicate internalized NPs). Subcutaneous breast cancer T_1_WI and corresponding SIpost/SIpre ratios (post- to pre-injection signal value ratios extracted from tumor regions) in 4T1 tumor-bearing mice following an intravenous injection of (**J, K**) Gd-DTPA, (**L, M**) RM NPs with 100 μL 20% glucose gavage, and RM NPs. Imaging parameters (conventional spin echo sequence): TE = 9 ms, TR = 513 ms, slice thickness = 0.4 mm, spacing = 2 mm, FOV = 80 mm, averages = 2 and flip angle = 90°. Statistical significance (independent samples *t*-test): *P < 0.05, **P < 0.01, ***P < 0.001 vs. control, n.s.: no significance. Data are expressed as mean ± SD (*n* = 3).

To assess the targeting capability of rutin on RM NPs toward Glut transporters, the cellular uptake of the MRI contrast agent was assessed. This uptake is typically evaluated by measuring cellular relaxation times (T_1_/T_2_) and quantifying the intracellular content of metal ions, such as Fe, Mn or Gd [53–55]. 4T1 and RAW 264.7 cells were incubated with RM NPs and a range of Mn concentrations (10, 30, 50 and 80 µg/mL) for 2 h. Preincubation of 4T1 cells with 20 mM glucose (to competitively inhibit Glut receptors) resulted in darker T_1_WI signals and higher T_1_ values compared with nonglucose-treated cells (Figure 5A and B). This was accompanied by significantly lower intracellular Mn content as measured using AAS (Figure 5C). Bio-TEM confirmed the reduced presence of RM NPs in the glucose-pretreated 4T1 cytoplasm (Figure 5I). Conversely, RAW 264.7 cells exhibited lower basal Mn uptake than 4T1 cells (Figure 5G), and glucose pretreatment had a negligible impact on RM NP uptake (Figure 5E–G), which is consistent with decreased Glut expression on macrophages. Further Western blot analysis also indicated that the expression level of GLUT1 on 4T1 cells was significantly higher than that on RAW 264.7 cells (Supplementary Figure S7).

To further validate the GLUT-mediated targeting, we conducted additional experiments using quercetin as a control ligand. Quercetin and rutin are structurally similar polyphenols, with the key difference that rutin contains two glycoside moieties, whereas quercetin is aglycone. To specifically test the role of the glycoside groups in targeting, we synthesized quercetin-coated manganese carbonate nanoparticles (QM NPs) using the same molar amount of quercetin. As shown in Supplementary Figure S8A and B, QM NPs exhibited a similar morphology and hydrodynamic diameter (250.6 ± 89.95 nm). The UV–vis spectrum (Supplementary Figure S8C) confirmed successful coating, indicated by the characteristic elevation at 374 nm corresponding to quercetin. Cellular uptake was then compared between RM NPs and QM NPs. As shown in Supplementary Figure S8E–G, the measured T_1_ values (at 3.0 T) and intracellular manganese content showed a statistically significant increase for RM NPs over QM NPs, supporting the enhanced targeting capability imparted by rutin’s glycoside groups toward GLUT.

### *In vivo* targeting and biodistribution in subcutaneous breast cancer model

Female BALB/c (6–8 weeks) bearing subcutaneous 4T1 tumors (∼50 mm^3^) in the left axilla were randomized (*n* = 12/group). One group received an oral gavage of 20% glucose solution 15–20 min before tail vein injection of RM NPs (0.1 mmol Mn/kg) to increase blood glucose levels and block Glut. The control group only received RM NPs. The mice (*n* = 3/time point/group) were sacrificed at 1, 2, 3 and 6 h post injection. Tumors and major organs (liver, spleen and kidney) were weighed, digested and analysed for Mn content via ICP-MS. Increased Mn accumulation was observed in tumors in the nonglucose group at 1 and 2 h (Figure 5D). Tumor Mn levels peaked at 2 h, followed by a decrease and stabilization. Pharmacokinetic analysis revealed that RM NPs were rapidly cleared from the bloodstream, with a Mn half-life (T_1/2_) of approximately 4.95 min (Supplementary Figure S9), indicating fast removal of the nanoparticles from circulation. Biodistribution analysis revealed that RM NPs were primarily metabolized by the liver and spleen (Mn levels increased initially, then decreased by 6 h), whereas partially degraded Mn^2+^ was excreted renally (Figure 5H). MRI biodistribution corroborated the hepatic clearance and renal excretion of Mn^2+^, with a signal decrease by 6 h, as evidenced by the sharp increase and subsequent gradual decrease of the gallbladder signal from 0.5 h onwards, confirming that RM NPs can also be cleared via biliary excretion (Supplementary Figure S10). These results confirm Glut-mediated tumor targeting and a favorable clearance profile for the RM NPs.

### *In vivo* MRI of subcutaneous breast cancer

To examine the targeting and MRI capability of the RM NPs, subcutaneous 4T1 tumors (∼50 mm^3^) were imaged. The Gd-based contrast agent Gd-DTPA served as a control. Post Gd-DTPA injection (0.1 mmol Gd/kg), the tumor signal-to-liver signal ratio (TLR = SI post, tumor/SI_post, liver) peaked at 30 min (TLR = 1.42), followed by a decrease over 6 h (Figure 5J and K), thus reflecting the rapid clearance of small molecules [56]. Post RM NP injection (0.1 mmol Mn/kg), tumor TLR increased, peaked at approximately 2 h (TLR = 1.50), which exceeded Gd-DTPA, and then gradually decreased (Figure 5L and M), which is consistent with tumor Mn^2+^ biodistribution (Figure 5D). Glucose pretreatment significantly reduced tumor TLR at 1 and 2 h (Figure 5L and M), which was consistent with reduced *in vivo* targeting (Figure 5D). Overall, RM NPs showed effective tumor targeting and comparable MRI performance to clinical Gd-DTPA.

### MRI of BCLM

**Figure 6:**
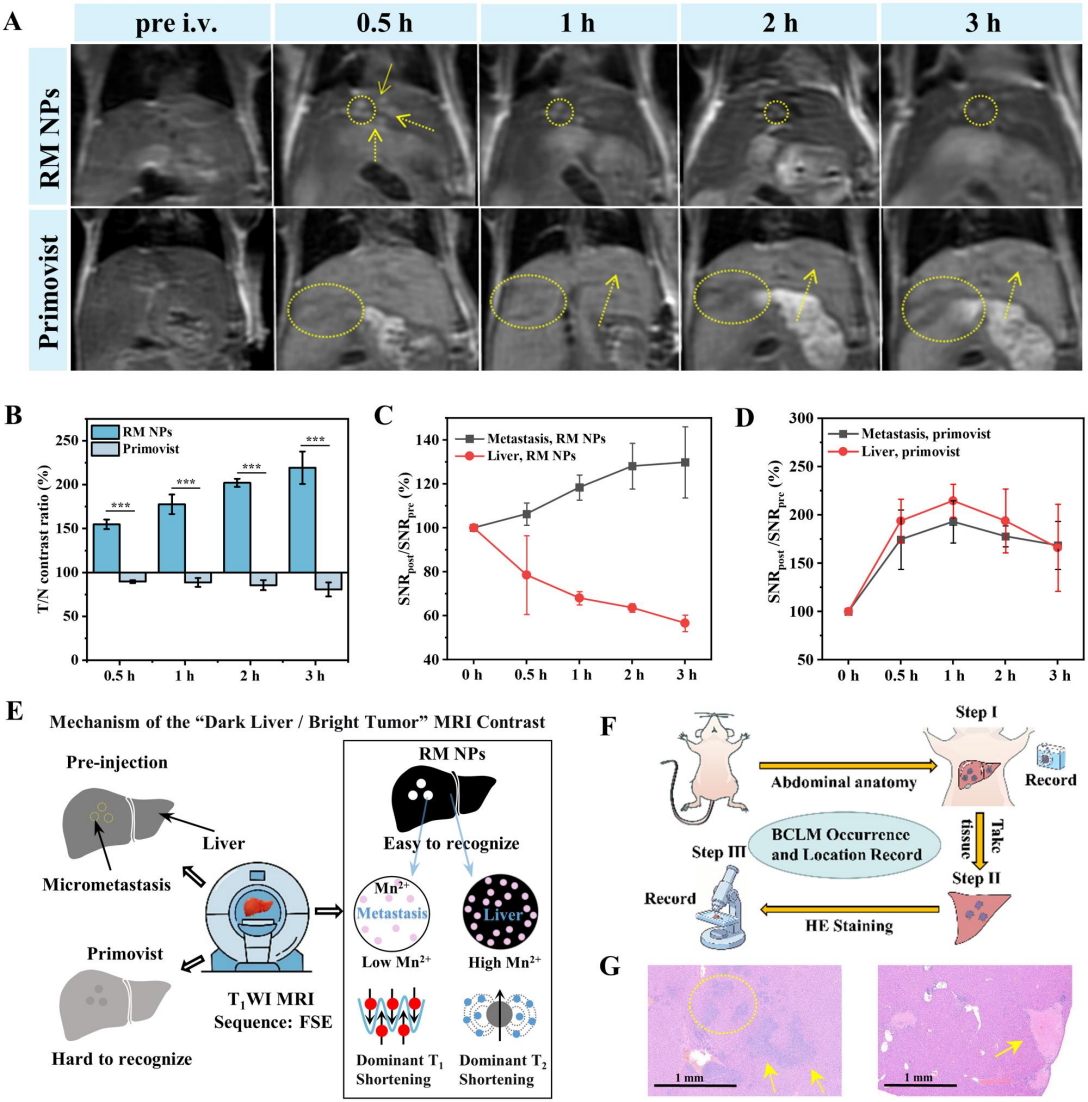
Assessment of the imaging capabilities of BCLM. RM NPs specifically enhance the contrast of LM. (**A**) T_1_-weighted MR images acquired using a 3.0 T scanner at representative time points following an intravenous injection of RM NPs and Primovist. The metastases (circles) are easily recognized. (**B**) Contrast intensity ratios indicate an ultrahigh contrast of 219% in the metastases for RM NPs but a negative contrast for Primovist. (**C, D**) SNR analysis of metastatic tumors and liver tissues following the administration of these probes. In contrast to Primovist, RM NPs cause a relaxivity increase in metastases and a concomitant *T*_2_ shortening effect in normal liver tissue. (**E**) Illustrates the imaging mechanism of RM nanoparticles in T_1_-weighted MRI of BCLM and highlights their superior performance compared to Primovist, shown as a round area in the liver. (**F**) Establishment of the BCLM model and experimental workflow for pathological verification. (**G**) Microscopic (H&E staining) images of the liver confirm the presence. Data are shown as mean ± SD (*n* = 3/group). *P < 0.05, **P < 0.01, ***P < 0.001 vs. control, n.s.: no significance. LM imaging at 3.0 T (GE scanner) using a T_1_-weighted rapid acquisition with relaxation enhancement (RARE) sequence with the following parameters: TE = 17 ms, TR = 480 ms, slice thickness = 0.4 mm, spacing between slices = 0.8 mm, ETL = 3, FOV = 100 mm, number of averages = 2 and flip angle = 142°.

Because distant metastasis, particularly to the liver (50–70% of cases) [2], accounts for most breast cancer-related deaths [57], the precise detection of micrometastases (<1 mm) is important. The clinical liver-specific agent Primovist was used as a control. Mice with established 4T1 liver micrometastases (induced by splenic injection of 1 × 10^6^ cells 4–5 days prior) were subjected to abdominal RM NP injection (0.1 mmol Mn/kg) before and after T_1_WI. The pre-injection images displayed indistinguishable lesions. At 30 min post injection, lesions (circles/arrows) were clearly visible (Figure 6A). The tumor-to-normal liver contrast ratio (T/N) reached 154% at 30 min, increasing to a peak of 219% by 3 h (Figure 6B). This high contrast resulted from opposing signal trends. Tumor SNR increased from 106% at 30 min to 128% at 2 h (indicating rapid microenvironment response), whereas liver SNR decreased from 79% to 64% over the same period (Figure 6C), thereby creating a bright lesion/dark background (‘positive contrast’) effect (Figure 6A). The signal decrease in the liver was attributed to T_2_ shortening effects from the high local Mn^2+^ concentration accumulated within Kupffer cells and hepatocytes. However, because of the specific uptake of Primovist by hepatocytes, the benign liver region gradually brightened. Primovist yielded lower BCLM lesion SNR than liver tissue (‘negative contrast’), indicating a poor distinction of micrometastases and vasculature (Figure 6A and D). In contrast, RM NPs displayed a higher imaging contrast than Primovist. The MRI effect of RM NPs can be visualized through a flowchart (Figure 6E). As shown in the workflow in Figure 6F, the first step involved abdominal dissection and photography to confirm that the location of BCLM was consistent with the MRI findings. In the second step, liver tissue containing BCLM was collected. Finally, the harvested liver tissue with BCLM was subjected to H&E staining. The results successfully confirmed the presence of BCLM, and its location was consistent with that observed in MRI (Figure 6G and Supplementary Figure S11).

In MRI, T_1_WI and T_2_WI are determined relative to the TE and TR values. Next, we identified the mechanism through which RM NPs can achieve the ‘dark liver, bright tumor’ phenomenon. Consistent with the TE value in the abdominal T_1_WI sequence of LM, this TE value was fixed, and a series of RM NPs with different Mn concentrations (0.02–0.5 mM) was prepared in a mildly acidic solution (pH 6.6). The results indicated that within the TR range of 400–600 ms, RM NPs exhibited high relative SI at all concentrations, which increased with increasing Mn concentration. This occurred because the T_1_WI effect dominates under these conditions. Based on the signal intensity formula (Figure 1), shortening of the T_1_ value results in an increased T_1_WI effect, thereby increasing the SI (Supplementary Figure S12A–C). However, as the TR value increased from 600 ms, the T_2_WI component became more pronounced, and the relative SI for RM NPs at all concentrations gradually decreased (Supplementary Figure S12A–C). This occurred because as the TR value increases, the high Mn^2+^ concentration yields a greater T_2_ shortening effect. Based on the formula, a shortening of the T_2_ value results in a decrease in the *A* term, thereby reducing SI (Figure 1). Notably, RM NPs with high Mn concentrations exhibit a rapid decline in the relative signal. This is attributed to the enhanced T_2_-shortening effect associated with higher Mn^2+^ concentrations as the TR value increases (Supplementary Figure S12A–C). Normal liver tissue contains a large number of hepatocytes and Kupffer cells, which take up more RM NPs than liver tumor tissue. This results in a higher concentration of Mn^2+^ in normal liver parenchyma. Notably, as shown in Supplementary Figure S6, the manganese ions released from RM NPs via acid-responsive degradation also exhibit excellent *r*_2_ relaxivity, contributing to a strong T_2_-shortening effect in regions with high Mn^2+^ accumulation. To maximize the hyperintense presentation of metastases in T_1_-weighted images, we fixed the TE value and minimized it, thereby enhancing T_1_-weighting while suppressing T_2_-weighted influence. As illustrated in the signal intensity curve in Figure 1, selecting an optimal TR value and exploiting the differences in Mn^2+^ content between tissues result in pronounced signal differences (△SI) between the two tissues. In particular, a high Mn^2+^ content in the liver exhibits a significant T_2_-shortening effect. Based on the signal intensity formula, this causes the SI in the liver to decrease. In contrast, the relatively low Mn^2+^ content in the tumor primarily presents T_1_WI imaging characteristics. Based on the formula, the SI increases. As a result, the ‘dark liver, bright tumor’ signal difference is evident on MRI.

### Apoptosis induction in 4T1 cells by RM NPs

**Figure 7:**
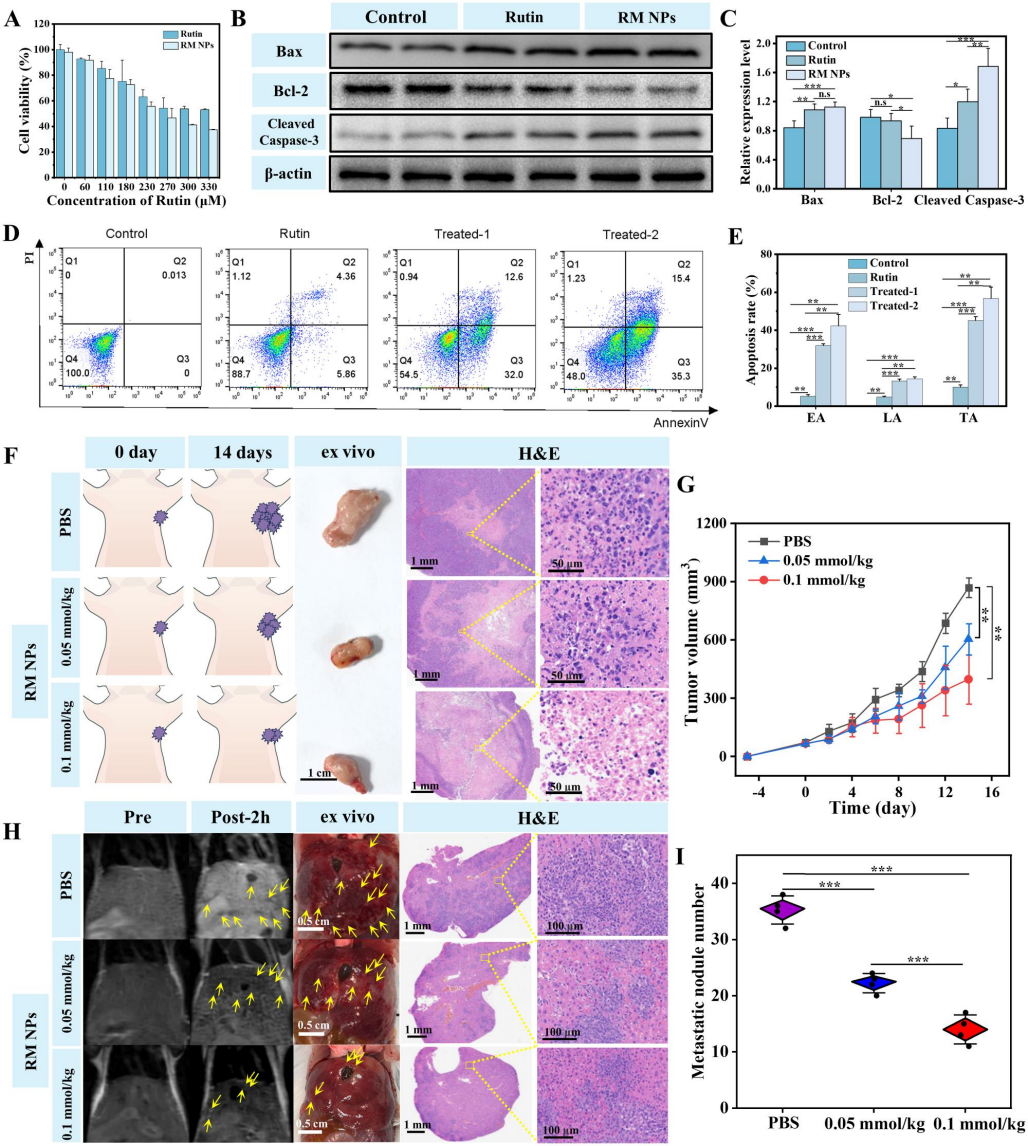
Evaluation of the therapeutic efficacy and mechanisms of RM NPs in subcutaneous breast cancer and BCLM. (**A**) Cytotoxicity of RM NPs and free rutin against 4T1 cells as assessed using the CCK-8 assay (*n* = 3 per well). (**B**) Western blot images and (**C**) relative expression levels of apoptosis-related proteins (Bax, Bcl-2 and cleaved Caspase-3) normalized to β-actin (*n* = 4 per group). (**D, E**) Flow cytometry plots quantifying apoptosis in 4T1 cells across treatment groups. Treated-2: cells were treated with the half-maximal inhibitory concentration (IC_50_) of RM NPs (*n* = 4 per group); Treated-1: half the IC_50_ concentration. (**F**) Schematic representation of the 4T1 subcutaneous tumor mouse model and excised tumors following PBS and RM NP treatments for 14 days. H&E staining indicates severe apoptosis in the tumor tissues treated with RM NPs. (**G**) Relative tumor volume changes in mice during treatment (*n* = 4 per group). (**H**) Abdominal T_1_-weighted MR images of LM at day 8 pre and post injection of RM NPs (0.1 mmol/kg Mn and 0.05 mmol/kg Mn) and PBS, *ex vivo* images, and H&E staining of the excised livers on day 8. Arrows indicate metastatic nodules. (**I**) The number of visually detected liver metastatic nodules (*n* = 4 per group). Statistical significance was calculated using a one-way analysis of variance (ANOVA). *P < 0.05, **P < 0.01, ***P < 0.001 vs. control. Data are expressed as mean ± SD (*n* = 4 per group). The parameters for abdominal T_1_-weighted imaging were as follows: TE = 17 ms, TR = 480 ms, slice thickness = 0.4 mm, spacing between slices = 0.8 mm, ETL = 3, FOV = 100 mm, number of averages = 2 and flip angle = 142°.

Rutin is a naturally occurring flavonoid that is ubiquitous in fruits and vegetables. It exhibits significant inhibitory effects against various cancer cells, including breast cancer, leukemia, liver cancer, colorectal cancer, endometrial cancer and gastric cancer cells [58]. However, its poor solubility results in low bioavailability and suboptimal absorption. The encapsulation of rutin within nanoparticles significantly enhances its bioavailability and efficacy [59]. CCK8 cytotoxicity assays revealed that RM NPs reduced the viability of 4T1 cells in a concentration-dependent manner, which is consistent with the trend observed with free rutin. The half-maximal inhibitory concentration (IC_50_) of RM NPs against 4T1 cells was 133.52 ± 10.47 µg/mL (Figure 7A).

To determine the apoptotic effect of RM NPs on 4T1 cells, apoptosis was assessed by Annexin V-FITC and propidium iodide staining followed by flow cytometry after a 24 h incubation with RM NPs. The cells were treated with an IC_50_ concentration of RM NPs (designated Treated-2) and half the IC_50_ concentration (designated Treated-1). The negative and blank control groups, which consisted of cells treated with free rutin at Treated-2 concentration and untreated cells, respectively, were included (*n* = 4 per well). The results indicated that incubation with RM NPs induced significant apoptosis in 4T1 cells compared with the blank control. Moreover, the apoptosis rates for Treated-1 and Treated-2 groups were significantly higher compared with those of the negative control (free rutin group) (Figure 7D and E). This enhanced apoptotic effect is likely attributable to the nanoparticle formulation facilitating the enhanced cellular uptake and absorption of rutin. In addition, the release of Mn^2+^ from the nanoparticles may synergistically induce apoptosis through the modulation of oxidative stress, possibly through mechanisms, such as the Fenton reaction. To verify this hypothesis, 4T1 cells were treated for 24 h with Mn^2+^, Rutin or RM NPs, each at their respective IC_50_ concentrations—consistent with the flow-cytometry apoptosis assay in Figure 7D. The cells were then stained with JC-1 and imaged by confocal microscopy, with untreated cells as the control group and CCCP-treated cells as the positive control. The results showed the presence of green fluorescent JC-1 monomers in the free Mn^2+^ treatment group, confirming that Mn^2+^ alone can also induce apoptosis in 4T1 cells. Moreover, all three treatment groups exhibited apoptotic effects (Supplementary Figure S13).

### Western blot analysis of apoptosis induced by RM NPs

To maintain consistency with the aforementioned apoptosis induction protocol, 4T1 cells were incubated with RM NPs at their IC_50_ value for 24 h. An equivalent concentration of rutin served as the drug control, whereas untreated 4T1 cells were designated as the blank control. This design further indicated that RM NPs regulate the expression of apoptosis-related proteins. Within the slightly acidic tumor microenvironment of 4T1 cells, RM NPs released rutin molecules, which induced apoptosis by attenuating levels of Bcl-2 family proteins and cleaved Caspase-3. This mechanism is consistent with the established antitumor effects of multiple flavonoids. Post-release, rutin significantly upregulated Bax and suppressed Bcl-2 levels relative to the blank control (Figure 7B and C), thus triggering mitochondrial outer membrane permeabilization. Consistent with the reported mechanisms of flavonoids, such as luteolin [60] and xanthohumol [61], which involve Bax/Bcl-2 modulation and cytochrome c release resulting in caspase-9/3 activation, our results further support the Bax/Bcl-2–Caspase signaling axis as a primary mechanism for flavonoid-induced apoptosis. The elevated cleaved Caspase-3 levels (Figure 7B and C) confirmed the irreversible commitment to apoptosis following RM NP treatment. Importantly, RM NP-treated 4T1 cells exhibited significantly higher cleaved Caspase-3 expression compared with the rutin control group (Figure 7B and C), thus demonstrating enhanced apoptotic efficacy. This indicates that the rutin-loaded nano-delivery system promotes intracellular drug accumulation in tumor cells, thereby improving rutin bioavailability.

Furthermore, rutin also exerts multi-target anticancer effects by modulating several key signaling pathways, including Wnt/β-catenin, JAK-STAT, EGFR, AP-1 and NF-κB. Specifically, rutin downregulates matrix metalloproteinase-2 (MMP-2) expression, inhibits extracellular matrix-mediated adhesion and migration, and modulates intracellular ROS levels, collectively contributing to its antiproliferative, anti-migratory and pro-apoptotic activities *in vitro*. These multi-faceted mechanisms synergistically enhance the comprehensive therapeutic efficacy of rutin in the tumor microenvironment, providing a coherent molecular explanation for the significant tumor suppression observed in the 4T1 xenograft model following RM NPs treatment [62, 63].

### *In vivo* therapy for subcutaneous and metastatic breast cancer

Mice bearing subcutaneous 4T1 tumors (approximately 50–80 mm^3^, *n* = 4/group) were administered PBS (control) or RM NPs (0.05 or 0.1 mmol Mn/kg) via the tail vein on days 0, 3 and 6. The tumor volume was measured every 2 days for 14 days. RM NPs significantly inhibited tumor growth (Figure 7G) and reduced the final tumor weight (Supplementary Figure S14A) and size (Supplementary Figure S15) compared with the control, thereby exhibiting a dose-dependent effect. Tumor anatomy and gross morphology visually confirmed growth suppression (Figure 7F and Supplementary Figure S16). Mouse body weight remained stable (Supplementary Figure S14B). H&E staining of the treated tumors revealed characteristic apoptotic features, including nuclear pyknosis, karyorrhexis, karyolysis, cytoplasmic condensation and enhanced eosinophilia (Figure 7F).

Mice with established 4T1 liver metastases (induced 4 days prior through splenic injection of 1 × 10^6^ cells, *n* = 4/group) were administered a single dose of PBS (control) or RM NPs (0.05 or 0.1 mmol Mn/kg). On day 8, the mice were administered RM NPs (0.1 mmol Mn/kg) for abdominal T_1_WI (pre and post 2 h). The control mice (post 2 h) exhibited a high liver background signal with numerous hypointense metastatic spots (arrows, Figure 7H), which were confirmed by extensive metastases on liver anatomy and H&E staining (Figure 7H). In contrast, the treated mice (post 2 h) displayed a low liver background signal and hyperintense metastases. RM NPs significantly reduced metastasis number in a dose-dependent manner: 22.3 ± 1.7 (0.05 mmol/kg) and 14.0 ± 2.6 (0.1 mmol/kg) vs. 35.3 ± 2.5 in the control (Figure 7I). This was confirmed through *ex vivo* liver examination and H&E staining (Figure 7H, Supplementary Figures S17 and S18).

### Mechanism of variable liver signal

We hypothesized that the differential hepatic background MRI signal observed in the experimental groups is attributed to variations in the extent of liver injury. This inconsistency in liver damage results in the heterogeneous uptake of RM NPs by hepatic tissue and the differential release of Mn^2+^. Consequently, the Mn^2+^-mediated T_2_ shortening effect exhibited corresponding variations. To test this, the following mouse (*n* = 3/group) experiment was conducted: control (saline splenic injection), early stage (4T1 splenic injection, sacrificed day 4) and late stage (4T1 splenic injection, sacrificed day 8). All received RM NPs (0.1 mmol Mn/kg) and were sacrificed 2 h after liver ICP-MS analysis. To ensure comparability, the left liver lobe (a superficial and anatomically consistent site) was uniformly sampled across all animals to minimize regional variability in hepatic function and nanoparticle uptake. The 2 h time point was selected to align with the peak tumor Mn accumulation observed in our prior biodistribution study (Figure 5D) and the maximal MRI signal contrast in both metastatic and subcutaneous tumor models, thereby maintaining temporal consistency across the experimental logic. Early stage metastatic livers showed the highest Mn content, which exceeded the control and late stage groups (Supplementary Figure S19). Bio-TEM confirmed higher RM NP accumulation (yellow arrows) in the early stage livers (Supplementary Figure S20). Reduced lipid droplet formation in hepatocytes near early metastases suggested that energy demands depleted the lipid reserves [64, 65]. During the early phase of BCLM, a structurally incomplete neovasculature exhibits high permeability. This characteristic likely enables nanoparticles to extravasate more readily and accumulate within the metastatic lesions through an enhanced permeability and retention effect. Furthermore, Kupffer cells exhibit increased phagocytic activity at this stage, which contributes to enhanced nanoparticle uptake [66]. Late-stage livers showed reduced uptake, which was likely the result of decreased perfusion, necrosis/fibrosis in large lesions and disrupted Kupffer cell function within the suppressive tumor microenvironment.

### RM NPs improve prognosis in a metastatic breast cancer model

Advanced BCLM can obstruct hepatic veins (draining to the right atrium), resulting in portal hypertension, ascites, hepatomegaly and cirrhosis [67]. To assess the effect of RM NPs on prognosis, mice (*n* = 3/group) with BCLM (established by day 4) were administered RM NPs (0.05 mmol Mn/kg) on days 4 and 8 or PBS (control). On day 12, they were administered RM NPs (0.1 mmol Mn/kg), and abdominal MRI (pre, post 2 h) was conducted to evaluate liver size and ascites. MRI and *ex vivo* anatomy revealed significantly smaller livers in the treated group than in the control group (Supplementary Figures S21, S22A and S23). Post-2 h MRI also revealed a heterogeneous liver signal and perhepatic fluid (indicating ascites) in the control group, which was correlated with visibly distended abdomens (Supplementary Figures S21 and S24). The treated mice had smaller abdomens. H&E staining confirmed a reduced metastasis burden in the treated group (Supplementary Figure S22B). RM NPs ameliorated complications such as ascites and hepatomegaly, suppressed metastasis growth and potentially improved survival and quality of life.

### Structure–activity relationship of stimuli-responsive nanoprobes for MRI

Constructing stimuli-responsive nanomaterials by leveraging a reasonable microenvironment is beneficial for enhancing the specificity of disease diagnosis. For instance, manganese-based materials (e.g. MnO_2_, MnCO_3_) can dissolve in weakly acidic environments and release Mn^2+^ with high T_1_ relaxivity, while ultrasmall Fe_3_O_4_ nanoparticles can serve as T_1_ contrast agents under appropriate magnetic fields and undergo aggregation upon GSH stimulation to switch their relaxation behavior to T_2_ contrast. Surface chemistry and functionalization are key to regulating response specificity and kinetics. The introduction of pH-sensitive bonds (e.g. hydrazone, imine) or environment-sensitive polymers as ‘gates’ enables controlled payload release. Meanwhile, grafting targeting ligands (e.g. folate, peptides) enhances active targeting efficiency and influences intracellular response processes. Nanomorphology, size and assembly structure directly affect the signal-output mode. Small-size, high-surface-area probes favor T_1_ contrast enhancement, whereas larger aggregates tend to strengthen T_2_ effects. By constructing smart architectures such as core–shell or hollow structures, the switch from ‘isolated’ to ‘released’ states of relaxing ions can be achieved, thereby transforming the relaxation mode (e.g. T_2_ to T_1_) into a readable activation signal. In theranostic design, the spatial arrangement and release mechanisms of imaging and therapeutic units must be coordinately engineered to ensure that stimulus-triggered imaging signal enhancement and therapeutic drug release are highly synchronized in time and space. This allows MRI not only to localize lesions but also to monitor the treatment process in real time, enabling early efficacy assessment [68, 69].

## Conclusion

In summary, pH-responsive RM NPs were successfully synthesized using a simple, one-pot mineralization approach. The synthesized RM NPs exhibited favorable biocompatibility, colloidal stability and precise pH-responsive degradation within the tumor microenvironment (pH 6.4–6.8). They effectively targeted Glut receptors overexpressed on breast cancer cells. Acid-triggered Mn^2+^ release significantly enhanced longitudinal relaxivity (*r*_1_), which enabled high-contrast T_1_-weighted MRI of primary breast tumors. Notably, exploiting the T_2_-shortening effect of accumulated Mn^2+^ in normal liver parenchyma resulted in an ultrahigh tumor-to-liver contrast ratio (219%), thereby facilitating the clear detection of micrometastases (<1 mm). Simultaneously, the released rutin exerted potent antitumor effects by modulating the mitochondrial apoptosis pathway (Bax/Bcl-2 ratio, Caspase-3 activation) and suppressing the growth of primary tumors and liver metastases. Furthermore, RM NPs ameliorated the complications associated with advanced metastatic disease (e.g. ascites and hepatomegaly), demonstrating their potential to improve patient prognosis. Biodegradable RM NPs are also efficiently cleared via the hepatic and renal pathways. This biocompatible theranostic platform, which features a straightforward synthesis, significantly enhances MRI sensitivity for detecting liver metastases while offering therapeutic benefits and improved prognosis for patients with BCLM.

## Supplementary Material

rbag049_Supplementary_Data

## Data Availability

Data will be made available on request.

## References

[1] Milette S , Sicklick JK, Lowy AM, Brodt P. Molecular pathways: targeting the microenvironment of liver metastases. Clin Cancer Res 2017;23:6390–9. 28615370 10.1158/1078-0432.CCR-15-1636 PMC5668192

[2] Cummings MC , Simpson PT, Reid LE, Jayanthan J, Skerman J, Song S, McCart Reed AE, Kutasovic JR, Morey AL, Marquart L, O’Rourke P, Lakhani SR. Metastatic progression of breast cancer: insights from 50 years of autopsies. J Pathol 2014;232:23–31. 24122263 10.1002/path.4288 PMC4288974

[3] Adam R , Aloia T, Krissat J, Bralet M-P, Paule B, Giacchetti S, Delvart V, Azoulay D, Bismuth H, Castaing D. Is liver resection justified for patients with hepatic metastases from breast cancer. Ann Surg 2006;244:897–907. 17122615 10.1097/01.sla.0000246847.02058.1b PMC1856635

[4] Xu X , Zhou X, Xiao B, Xu H, Hu D, Qian Y, Hu H, Zhou Z, Liu X, Gao J, Slater NKH, Shen Y, Tang J. Glutathione-responsive magnetic nanoparticles for highly sensitive diagnosis of liver metastases. Nano Lett 2021;21:2199–206. 33600181 10.1021/acs.nanolett.0c04967

[5] Renzulli M , Clemente A, Ierardi AM, Pettinari I, Tovoli F, Brocchi S, Peta G, Cappabianca S, Carrafiello G, Golfieri R. Imaging of colorectal liver metastases: new developments and pending issues. Cancers (Basel) 2020;12:151–62. 31936319 10.3390/cancers12010151 PMC7017094

[6] Kuo PH , Kanal E, Abu-Alfa AK, Cowper SE. Gadolinium-based MR contrast agents and nephrogenic systemic fibrosis. Radiology 2007;242:647–9. 17213364 10.1148/radiol.2423061640

[7] Li JJ , Wu C, Hou PF, Zhang M, Xu K. One-pot preparation of hydrophilic manganese oxide nanoparticles as T1 nano-contrast agent for molecular magnetic resonance imaging of renal carcinoma in vitro and in vivo. Biosens Bioelectron 2018;102:1–8. 29101783 10.1016/j.bios.2017.10.047

[8] Gulani V , Calamante F, Shellock FG, Kanal E, Reeder SB; International Society for Magnetic Resonance in Medicine. Gadolinium deposition in the brain: summary of evidence and recommendations. Lancet Neurol 2017;16:564–70. 28653648 10.1016/S1474-4422(17)30158-8

[9] Ronot M , Clift AK, Vilgrain V, Frilling A. Functional imaging in liver tumours. J Hepatol 2016;65:1017–30. 27395013 10.1016/j.jhep.2016.06.024

[10] Wu C , Chen W, Yan S, Zhong J, Du L, Yang C, Pu Y, Li Y, Lin J, Zeng M, Zhang X. MRI-guided photothermal/photodynamic immune activation combined with PD-1 inhibitor for the multimodal combination therapy of melanoma and metastases. Regen Biomater 2024;11:rbae019. 38525327 10.1093/rb/rbae019 PMC10960927

[11] Lu F , Du L, Chen W, Jiang H, Yang C, Pu Y, Wu J, Zhu J, Chen T, Zhang X, Wu C. T1–T2 dual-modal magnetic resonance contrast-enhanced imaging for rat liver fibrosis stage. RSC Adv 2022;12:35809–19. 36545112 10.1039/d2ra05913d PMC9749127

[12] Jiao M , Zhang P, Meng J, Li Y, Liu C, Luo X, Gao M. Recent advancements in biocompatible inorganic nanoparticles towards biomedical applications. Biomater Sci 2018;6:726–45. 29308496 10.1039/c7bm01020f

[13] Colombo M , Carregal-Romero S, Casula MF, Gutiérrez L, Morales MP, Böhm IB, Heverhagen JT, Prosperi D, Parak WJ. Biological applications of magnetic nanoparticles. Chem Soc Rev 2012;41:4306–34. 22481569 10.1039/c2cs15337h

[14] Reddy LH , Arias JL, Nicolas J, Couvreur P. Magnetic nanoparticles: design and characterization, toxicity and biocompatibility, pharmaceutical and biomedical applications. Chem Rev 2012;112:5818–78. 23043508 10.1021/cr300068p

[15] Zhao Z , Wang X, Zhang Z, Zhang H, Liu H, Zhu X, Li H, Chi X, Yin Z, Gao J. Real-time monitoring of arsenic trioxide release and delivery by activatable T1 imaging. ACS Nano 2015;9:2749–59. 25688714 10.1021/nn506640h

[16] Zhang CH , Cai K, Zhang PG, Wu Z, Ma M, Chen B. pH-responsive DNA nanoassembly for detection and combined therapy of tumor. Biosens Bioelectron 2022;195:113654. 34592499 10.1016/j.bios.2021.113654

[17] Yu J , Zhao F, Gao W, Yang X, Ju Y, Zhao L, Guo W, Xie J, Liang X-J, Tao X, Li J, Ying Y, Li W, Zheng J, Qiao L, Xiong S, Mou X, Che S, Hou Y. Magnetic reactive oxygen species nanoreactor for switchable magnetic resonance imaging guided cancer therapy based on pH-sensitive Fe_5_C_2_@Fe_3_O_4_ nanoparticles. ACS Nano 2019;13:10002–14. 31433945 10.1021/acsnano.9b01740

[18] Zhou Z , Deng H, Yang W, Wang Z, Lin L, Munasinghe J, Jacobson O, Liu Y, Tang L, Ni Q, Kang F, Liu Y, Niu G, Bai R, Qian C, Song J, Chen X. Early stratification of radiotherapy response by activatable inflammation magnetic resonance imaging. Nat Commun 2020;11:3032–44. 32541769 10.1038/s41467-020-16771-y PMC7295999

[19] Tseng YJ , Chou SW, Shyue JJ, Lin SY, Hsiao JK, Chou PT. A versatile theranostic delivery platform integrating magnetic resonance imaging/computed tomography, pH/cis-diol controlled release, and targeted therapy. ACS Nano 2016;10:5809–22. 27163375 10.1021/acsnano.5b08130

[20] Hu Y , Wang Y, Wen X, Pan Y, Cheng X, An R, Gao G, Chen H-Y, Ye D. Responsive trimodal probes for in vivo imaging of liver inflammation by coassembly and GSH-driven disassembly. Research (Wash D C) 2020;2020:4087069. 33029587 10.34133/2020/4087069 PMC7520820

[21] Peng Y , Ye C, Yan R, Lei Y, Ye D, Hong H, Cai T. Activatable core–shell metallofullerene: an efficient nanoplatform for bimodal sensing of glutathione. ACS Appl Mater Interfaces 2019;11:46637–44. 31747242 10.1021/acsami.9b18807

[22] Zhu X , Lin H, Wang L, Tang X, Ma L, Chen Z, Gao J. Activatable T1 relaxivity recovery nanoconjugates for kinetic and sensitive analysis of matrix metalloprotease 2. ACS Appl Mater Interfaces 2017;9:21688–96. 28603956 10.1021/acsami.7b05389

[23] Gallo J , Kamaly N, Lavdas I, Stevens E, Nguyen Q-D, Wylezinska-Arridge M, Aboagye EO, Long NJ. CXCR4-targeted and MMP-responsive iron oxide nanoparticles for enhanced magnetic resonance imaging. Angew Chem Int Ed Engl 2014;53:9550–4. 25045009 10.1002/anie.201405442 PMC4321346

[24] Zhu X , Tang X, Lin H, Shi S, Xiong H, Zhou Q, Li A, Wang Q, Chen X, Gao J. A fluorinated ionic liquid-based activatable 19F MRI platform detects biological targets. Chem 2020;6:1134–48. 34084948 10.1016/j.chempr.2020.01.023 PMC8171808

[25] Yan R , Hu Y, Liu F, Wei S, Fang D, Shuhendler AJ, Liu H, Chen H-Y, Ye D. Activatable NIR fluorescence/MRI bimodal probes for in vivo imaging by enzyme-mediated fluorogenic reaction and self-assembly. J Am Chem Soc 2019;141:10331–41. 31244188 10.1021/jacs.9b03649

[26] Mercadante CJ , Prajapati M, Conboy HL, Dash ME, Herrera C, Pettiglio MA, Cintron-Rivera L, Salesky MA, Rao DB, Bartnikas TB. Manganese transporter Slc30a10 controls physiological manganese excretion and toxicity. J Clin Invest 2019;129:5442–61. 31527311 10.1172/JCI129710 PMC6877324

[27] Katz N , Rader DJ. Manganese homeostasis: from rare single-gene disorders to complex phenotypes and diseases. J Clin Invest 2019;129:5082–5. 31682237 10.1172/JCI133120 PMC6877306

[28] Wu C , Zhong J, Li J, Luo Y, Wang J, Zeng X, Mao J, Lu J, Xu J, Wu C, Wang Z. Facile construction of manganese-based contrast agent with high T1 relaxivity for magnetic resonance imaging via flash technology-based self-assembly. Regen Biomater 2025;12:rbaf009. 40270577 10.1093/rb/rbaf009 PMC12017619

[29] Li Y , Zhao X, Liu X. A bioinspired nanoprobe with multilevel responsive T1-weighted MR signal-amplification illuminates ultrasmall metastases. Adv Mater 2020;4:e1906799. 10.1002/adma.201906799 31799765

[30] Dai Y , Xu C, Sun X, Chen X. Nanoparticle design strategies for enhanced anticancer therapy by exploiting the tumour microenvironment. Chem Soc Rev 2017;46:3830–52. 28516983 10.1039/c6cs00592f PMC5521825

[31] Zhu X , Xiong H, Zhou Q, Zhao Z, Zhang Y, Li Y, Wang S, Shi S. A pH-activatable MnCO_3_ nanoparticle for improved magnetic resonance imaging of tumour malignancy and metastasis. ACS Appl Mater Interfaces 2021;13:18462–71. 33871955 10.1021/acsami.0c22624

[32] Birts CN , Banerjee A, Darley M, Dunlop CR, Nelson S, Nijjar SK, Parker R, West J, Tavassoli A, Rose-Zerilli MJJ, Blaydes JP. p53 is regulated by aerobic glycolysis in cancer cells by the CtBP family of NADH-dependent transcriptional regulators. Sci Signal 2020;13:eaau9529. 32371497 10.1126/scisignal.aau9529 PMC7244340

[33] Yang L , Li J, Li Y, Zhou Y, Wang Z, Zhang D, Liu J, Zhang X. Diclofenac impairs the proliferation and glucose metabolism of triple-negative breast cancer cells by targeting the c-Myc pathway. Exp Ther Med 2021;21:584–93. 33850556 10.3892/etm.2021.10016 PMC8027724

[34] Wu Q , Ba-Alawi W, Deblois G, Cruickshank J, Duan S, Lima-Fernandes E, Haight J, Tonekaboni SAM, Fortier A-M, Kuasne H, McKee TD, Mahmoud H, Kushida M, Cameron S, Dogan-Artun N, Chen W, Nie Y, Zhang LX, Vellanki RN, Zhou S, Prinos P, Wouters BG, Dirks PB, Done SJ, Park M, Cescon DW, Haibe-Kains B, Lupien M, Arrowsmith CH. GLUT inhibition blocks growth of RB1-positive triple negative breast cancer. Nat Commun 2020;11:4205–17. 32826891 10.1038/s41467-020-18020-8 PMC7442809

[35] Choi SS , Park HR, Lee KA. A comparative study of rutin and rutin glycoside: antioxidant activity, anti-inflammatory effect, effect on platelet aggregation and blood coagulation. Antioxidants 2021;10:1696. 34829567 10.3390/antiox10111696 PMC8614652

[36] Pandey P , Rahman M, Bhatt PC, Beg S, Paul B, Hafeez A, Al-Abbasi FA, Nadeem MS, Baothman O, Anwar F, Kumar V. Implication of nano-antioxidant therapy for treatment of hepatocellular carcinoma using PLGA nanoparticles of rutin. Nanomedicine (Lond) 2018;13:849–70. 29565220 10.2217/nnm-2017-0306

[37] Kersemans V , Wallington S, Allen PD. Manganese-free chow, a refined non-invasive solution to reduce gastrointestinal signal for T1-weighted magnetic resonance imaging of the mouse abdomen. Lab ANIM-UK 2019;4:353–64. 10.1177/0023677219869363 PMC7425378 31526094

[38] Jeong JJ , Ha YM, Jin YC, Lee EJ, Kim JS, Kim HJ, Seo HG, Lee JH, Kang SS, Kim YS, Chang KC. Rutin from lonicera japonica inhibits myocardial ischemia/reperfusion-induced apoptosis in vivo and protects H9c2 cells against hydrogen peroxide-mediated injury via ERK1/2 and PI3K/Akt signals in vitro. Food Chem Toxicol 2009;47:1569–76. 19362115 10.1016/j.fct.2009.03.044

[39] Ke H , Wang X, Zhou Z, Ai W, Wu Z, Zhang Y. Effect of weimaining on apoptosis and caspase-3 expression in a breast cancer mouse model. J Ethnopharmacol 2021;264:113363. 32916234 10.1016/j.jep.2020.113363

[40] Qi C, , He J, , Fu L-H, , He T, , Blum NT, , Yao X, , Lin J, , Huang P. Tumor-specific activatable nanocarriers with gas-generation and signal amplification capabilities for tumor theranostics. ACS Nano 2021;15:1627–39. 33356128 10.1021/acsnano.0c09223

[41] Cheng Y , Zhang S, Kang N, Huang J, Lv X, Wen K, Ye S, Chen Z, Zhou X, Ren L. Polydopamine-coated manganese carbonate nanoparticles for amplified magnetic resonance imaging-guided photothermal therapy. ACS Appl Mater Interfaces 2017;9:19296–306. 28508635 10.1021/acsami.7b03087

[42] Wu X , Caol M, Lü H, He X, Hu C. Microemulsion-mediated solvothermal synthesis and morphological evolution of MnCO_3_ nanocrystals. J Nanosci Nanotechnol 2006;6:2123–8. 17025136 10.1166/jnn.2006.371

[43] Zhou M , Zhang G, Hu J, Zhu Y, Lan H, Shen X, Lv Y, Huang L. Rutin attenuates sorafenib-induced chemoresistance and autophagy in hepatocellular carcinoma by regulating BANCR/miRNA-590-5P/OLR1 axis. Int J Biol Sci 2021;17:3595–607. 34512168 10.7150/ijbs.62471 PMC8416719

[44] Wilhelm S , Tavares AJ, Dai Q, Ohta S, Audet J, Dvorak HF, Chan WCW. Analysis of nanoparticle delivery to tumour. Nat Rev Mater 2016;1:16014.

[45] Peng S , Liu J, Qin Y, Wang H, Cao B, Lu L, Yu X. Metal–organic framework encapsulating hemoglobin as a high-stable and long-circulating oxygen carriers to treat hemorrhagic shock. ACS Appl Mater Interfaces 2019;11:35604–12. 31495166 10.1021/acsami.9b15037

[46] He JB , Wang Y, Deng N, Lin XQ. Study of the adsorption and oxidation of antioxidant rutin by cyclic voltammetry–voltabsorptometry. Bioelectrochemistry 2007;71:157–63. 17462963 10.1016/j.bioelechem.2007.03.003

[47] Zeng Y , Xu J, Wang Y, Li S, Luan D, Lou XWD. Formation of CuMn Prussian blue analog double-shelled nanoboxes toward long-life Zn-ion batteries. Angew Chem Int Ed Engl 2022;61:e202212031. 36177990 10.1002/anie.202212031

[48] Dong L , Zhang P, Lei P, Song S, Xu X, Du K, Feng J, Zhang H. Pegylated GdF3: Fe nanoparticles as multimodal T1/T2-weighted MRI and X-ray CT imaging contrast agents. ACS Appl Mater Interfaces 2017;9:20426–34. 28557419 10.1021/acsami.7b04438

[49] Wang S , Ma S, Li H, Dao M, Li X, Karniadakis GE. Two-component macrophage model for active phagocytosis with pseudopod formation. Biophys J 2024;123:1069–84. 38532625 10.1016/j.bpj.2024.03.026 PMC11079866

[50] Yan L , Wang J, Cai X, Liou Y-C, Shen H-M, Hao J, Huang C, Luo G, He W. Macrophage plasticity: signaling pathways, tissue repair, and regeneration. MedComm (2020) 2024;5:e658. 39092292 10.1002/mco2.658 PMC11292402

[51] Tang H , Li Q, Yan W, Jiang X. Reversing the chirality of surface ligands can improve the biosafety and pharmacokinetics of cationic gold nanoclusters. Angew Chem Weinheim Bergstr Ger 2021;133:13948–53. 10.1002/anie.202101609 33755292

[52] Pandey P , Khan F, Qari HA, Oves M. Rutin (bioflavonoid) as cell signaling pathway modulator: prospects in treatment and chemoprevention. Pharmaceuticals 2021;14:1069–84. 34832851 10.3390/ph14111069 PMC8621917

[53] Chen J , Zhang W-J, Guo Z, Wang H-B, Wang D-D, Zhou J-J, Chen Q-W. pH-responsive iron manganese silicate nanoparticles as T1–T2 dual-modal imaging probes for tumor diagnosis. ACS Appl Mater Interfaces 2015;7:5373–83. 25685956 10.1021/acsami.5b00727

[54] Hall RC , Qin J, Laney V, Ayat N, Lu ZR. Manganese (II) EOB-Pyclen diacetate for liver-specific MRI. ACS Appl Bio Mater 2022;5:451–8. 10.1021/acsabm.1c01259 35148050

[55] Li H , Hai Z, Zou L, Zhang L, Wang L, Wang L, Liang G. Simultaneous enhancement of T1 and T2 magnetic resonance imaging of liver tumor at respective low and high magnetic fields. Theranostics 2022;12:410–7. 34987653 10.7150/thno.67155 PMC8690926

[56] Chen H , Shou K, Chen S, Qu C, Wang Z, Jiang L, Zhu M, Ding B, Qian K, Ji A, Lou H, Tong L, Hsu A, Wang Y, Felsher DW, Hu Z, Tian J, Cheng Z. Smart self-assembly amphiphilic cyclopeptide-dye for near-infrared window-II imaging. Adv Mater 2021;33:e2006902. 33709533 10.1002/adma.202006902

[57] Zhang C , Qi L, Cai J, Wu H, Xu Y, Lin Y, Li Z, Chekhonin VP, Peltzer K, Cao M, Yin Z, Wang X, Ma W. Clinicomics-guided distant metastasis prediction in breast cancer via artificial intelligence. BMC Cancer 2023;23:239–55. 36918809 10.1186/s12885-023-10704-w PMC10012565

[58] Farha AK , Gan R-Y, Li H-B, Wu D-T, Atanasov AG, Gul K, Zhang J-R, Yang Q-Q, Corke H. The anticancer potential of the dietary polyphenol rutin: current status, challenges, and perspectives. Crit Rev Food Sci Nutr 2022;62:832–59. 33054344 10.1080/10408398.2020.1829541

[59] Gullón B , Lú-Chau TA, Moreira MT, Lema JM, Eibes G. Rutin: a review on extraction, identification and purification methods, biological activities and approaches to enhance its bioavailability. Trends Food Sci Tech 2017;67:220–35.

[60] Choi AY , Choi JH, Yoon H, Hwang K-Y, Noh MH, Choe W, Yoon K-S, Ha J, Yeo E-J, Kang I. Luteolin induces apoptosis through endoplasmic reticulum stress and mitochondrial dysfunction in neuro-2a mouse neuroblastoma cells. Eur J Pharmacol 2011;668:–115–26. 21762691 10.1016/j.ejphar.2011.06.047

[61] Guo D , Zhang B, Liu S, Jin M. Xanthohumol induces apoptosis via caspase activation, regulation of Bcl-2, and inhibition of PI3K/Akt/mTOR-kinase in human gastric cancer cells. Biomed Pharmacother 2018;106:1300–6. 30119200 10.1016/j.biopha.2018.06.166

[62] Imani A , Maleki N, Bohlouli S, Kouhsoltani M, Sharifi S, Maleki Dizaj S. Molecular mechanisms of anticancer effect of rutin. Phytother Res 2021;35:2500–13. 33295678 10.1002/ptr.6977

[63] Ben Sghaier M , Pagano A, Mousslim M, Ammari Y, Kovacic H, Luis J. Rutin inhibits proliferation, attenuates superoxide production and decreases adhesion and migration of human cancerous cells. Biomed Pharmacother 2016;84:1972–8. 27829548 10.1016/j.biopha.2016.11.001

[64] Li Y , Su X, Rohatgi N, Zhang Y, Brestoff JR, Shoghi KI, Xu Y, Semenkovich CF, Harris CA, Peterson LL, Weilbaecher KN, Teitelbaum SL, Zou W. Hepatic lipids promote liver metastasis. JCI Insight 2020;5:e136215. 32879136 10.1172/jci.insight.136215 PMC7487169

[65] Cruz ALS , Barreto EA, Fazolini NPB, Viola JPB, Bozza PT. Lipid droplets: platforms with multiple functions in cancer hallmarks. Cell Death Dis 2020;11:105–21. 32029741 10.1038/s41419-020-2297-3 PMC7005265

[66] Covarrubias G , Moon TJ, Loutrianakis G, Sims HM, Umapathy MP, Lorkowski ME, Bielecki PA, Wiese ML, Atukorale PU, Karathanasis E. Comparison of the uptake of untargeted and targeted immunostimulatory nanoparticles by immune cells in the microenvironment of metastatic breast cancer. J Mater Chem B 2022;10:224–35. 34846443 10.1039/d1tb02256c PMC8732314

[67] Diamond JR , Finlayson CA, Borges VF. Hepatic complications of breast cancer. Lancet Oncol 2009;10:615–21. 19482250 10.1016/S1470-2045(09)70029-4

[68] Wang Q , Liang Z, Li F, Lee J, Low LE, Ling D. Dynamically switchable magnetic resonance imaging contrast agents. Exploration (Beijing) 2021;1:20210009. 37323214 10.1002/EXP.20210009 PMC10191000

[69] Li X , Yue R, Guan G, Zhang C, Zhou Y, Song G. Recent development of pH-responsive theranostic nanoplatforms for magnetic resonance imaging-guided cancer therapy. Exploration (Beijing) 2023;3:20220002. 37933379 10.1002/EXP.20220002 PMC10624388

