# Supplementary material for: Rutin-encapsulated manganese carbonate nanoparticles for ‘liver-dark/tumor-bright’ magnetic resonance imaging and inhibition of breast cancer with liver metastases
Source: Regen Biomater. 2026 Mar 9;13:rbag049. doi: 10.1093/rb/rbag049 (PMC13143423; doi:10.1093/rb/rbag049)

Supporting Information

Rutin-encapsulated manganese carbonate nanoparticles for “Liver-dark/tumor-bright” magnetic resonance imaging and inhibition of breast cancer with liver metastases

Lingfeng Zhao^1†^, Yuanyuan Gao^1†^, Tao Chen^1^, Yongxi Li^1^, Aijia Chen^1^, Zhengju Hou^1^, Yu Pu^1,2^, Wenxue Li^1^, Xinghui Li^1,2^, Xiaoming Zhang^1,2,^*, Changqiang Wu^1,^*

^1^Medical Imaging Key Laboratory of Sichuan Province and School of Medical Imaging, North Sichuan Medical College, Nanchong 637000, P. R. China

^2^Department of Radiology, Affiliated Hospital of North Sichuan Medical College, Nanchong 637000, Sichuan, P. R. China

*Corresponding author:

Changqiang Wu, Medical Imaging Key Laboratory of Sichuan Province and School of Medical Imaging, North Sichuan Medical College, Nanchong 637000, Sichuan, P. R. China. E-mail: [wucq1984@nsmc.edu.cn](mailto:wucq1984@nsmc.edu.cn)

Xiaoming Zhang, Department of Radiology, Affiliated Hospital of North Sichuan Medical College, Nanchong 637000, Sichuan, P. R. China. E-mail: [zhangxm@nsmc.edu.cn](mailto:zhangxm@nsmc.edu.cn)

†These authors contributed equally to this work.

1. **Additional Experimental Details**
   1. **Materials and Reagents**

Rutin (95%, Aladdin), manganese (II) chloride tetrahydrate (MnCl₂·4H₂O, >99%, Thermo Fisher Scientific), dimethyl sulfoxide (DMSO, >99%, Aladdin), anhydrous sodium carbonate (Na₂CO₃, >99.8%, Chengdu Kelong), gadoxetic acid disodium salt (98%, Macklin), CCK-8 Assay Kit (500 tests, Beyotime), Annexin V-FITC Apoptosis Detection Kit (50 tests, Beyotime), and formaldehyde (37 wt.% in H₂O, stabilized with 10%–15% methanol, Macklin) were used in this study. All reagents were used as received without further purification. Ultrapure water (18.2 MΩ·cm) was obtained from a Milli-Q water purification system. The following primary antibodies were used: Bax Recombinant Rabbit Monoclonal Antibody (Bax, 21 kDa, HuaAn) (1:20,000), Bcl-2 Recombinant Rabbit Monoclonal Antibody (Bcl-2, 26 kDa, HuaAn) (1:5,000), Caspase-3 Recombinant Rabbit Monoclonal Antibody (Caspase-3, 32kDa, HuaAn) (1:2,000), Cleaved Caspase-3 (Asp175) Recombinant Rabbit Monoclonal Antibody (Cleaved Caspase-3, 17/19 kDa, Beyotime) (1:1,000), and β-Actin Recombinant Rabbit Monoclonal Antibody (β-Actin, 42 kDa, Beyotime) (1:2,000).

- 1. **Synthesis of RM NPs with varying rutin ligand concentrations**

RM NPs were synthesized using various rutin ligand concentrations (0, 0.3, 0.7, 3.3, 6.7, and 10 mg/mL). Rutin was dissolved in 10 mL dimethyl sulfoxide (DMSO) in a 50 mL round-bottom flask. Next, 50 mg MnCl₂·4H₂O was added with constant stirring (500 rpm) for 1 h at room temperature. Next, 5 mL of aqueous sodium carbonate solution (200 mM) was added dropwise under ultrasonication (40 kHz, 300 W). After 5 min of sonication, the reaction mixture was stirred continuously (500 rpm) for 4 h at 25°C. The resulting nanoparticles were collected via centrifugation at 8,000 rpm for 5 min and washed once with ethanol and twice with deionized water to yield rutin-coated manganese carbonate nanoparticles (RM NPs) for each designated rutin concentration. Particles synthesized without rutin (0 mg/mL) served as control manganese carbonate NPs.

- 1. **Characterization techniques**

The morphology and particle size of RM NPs were assessed using transmission electron microscopy (TEM, JEOL JEM F200, Japan). Surface morphology was assessed by scanning electron microscopy (SEM, ZEISS Gemini SEM 300, Germany). Successful synthesis of RM NPs was confirmed via Fourier-transform infrared spectroscopy (FTIR, Bruker Vertex 70, Germany) and ultraviolet-visible spectroscopy (UV-vis, Shimadzu UV-1900i, Japan). Hydrodynamic diameter and zeta potential were measured via dynamic light scattering (DLS, Malvern Zetasizer Nano ZS90, UK). Elemental composition, oxidation state, and crystalline structure were determined using X-ray photoelectron spectroscopy (Thermo Scientific K-Alpha, USA) and X-ray diffraction (Rigaku Ultima IV, Japan). Elemental distribution mapping was determined using energy-dispersive X-ray spectroscopy (EDS) coupled with TEM (JEOL JEM-F200, Japan). The optical density (OD) was measured using a microplate reader (Thermos Scientifics, Varioskan LUX, USA).

- 1. ***In Vitro* toxicity of RM NPs against 4T1 cells**

To determine the cytotoxic effect of the rutin component within RM NPs on murine breast cancer 4T1 cells, the concentration of manganese ions in the initial material solution was measured. The material was subsequently lyophilized and weighed to calculate the rutin content, which was further corroborated using EDS elemental mapping. The lyophilized material was reconstituted in a fixed volume of ultrapure water.

Using free rutin as a control, cytotoxicity was assessed by the CCK8 assay. Briefly, 4T1 cells were seeded into 96 well plates at a density of 10,000 cells per well and incubated for 24 h. The wells were designated as blank (medium only), control (medium + cells), and experimental (medium + cells + material). The old medium was replaced with fresh medium containing RM NPs at various rutin concentrations (60, 110, 180, 230, and 270 μmol/L). After an additional 24 h incubation, the medium was replaced with fresh medium containing 10% CCK8 reagent. The plates were incubated in the dark for 30 min. The OD at 450 nm was measured using a microplate reader. Cell viability was calculated using the following formula:

Cell viability = $\frac{{OD}_{\mathrm{sample}}-{OD}_{\mathrm{blank}}}{{OD}_{\mathrm{control}}-{OD}_{blank}}$ × 100%

- 1. **pH-responsive release** **profile and stability of RM NPs**

The Mn²⁺ release characteristics of the RM NPs were determined in PBS buffers with different pH values. In brief, 5 mg of lyophilized RM NPs were dissolved in 2.5 mL ultrapure water and sealed in dialysis bags (MWCO: 10 kDa). The bags were immersed in 47.5 mL PBS buffer (pH 7.4 or 6.6) with constant stirring. At predetermined time intervals, 1 mL aliquots were withdrawn and diluted with 2% nitric acid solution. Mn^2+^ concentration was quantified using AAS. The *in vitro*stability of the RM NPs was assessed using DLS. Samples were dispersed in either ultrapure water or 10% fetal bovine serum solution. Hydrodynamic diameter and polydispersity index (PDI) were measured using DLS at 2 day intervals over 14 days to evaluate colloidal stability. The analysis was performed using Origin software.

- 1. **T_1_ relaxivity measurement of RM NPs**

The T_1_ relaxation properties of RM NPs were determined at magnetic field strengths of 0.5 T, 1.41 T, and 3.0 T. The samples were prepared by reconstituting RM NPs synthesized with different amounts of rutin (50 mg, 100 mg, and 150 mg; designated RM-50, RM-100, and RM-150) to achieve 0.1–0.5 mM Mn²⁺ concentrations in a 1 mL solution. Mn²⁺ concentrations were verified using AAS. T_1_ relaxation times were measured in neutral (pH 7.4) and mildly acidic (pH 6.6) environments at each field strength. Relaxivity values (r₁) were calculated from the slope of the linear regression curve plotting 1/T_1_ relaxation time (s⁻¹) versus Mn²⁺ concentration (mM). At 3.0 T, T_1_ measurements used an inversion recovery fast spin-echo (IR-FSE) sequence with the following parameters: echo time (TE): 16 ms, repetition time (TR): 3000 ms, inversion time (TI): 50–1500 ms (incremental), slice thickness: 3 mm, field of view (FOV): 180 mm, echo train length (ETL): 24, and flip angle: 125°.

- 1. **pH-responsive relaxivity of RM NPs *In Vitro***

To confirm the time-dependent relaxivity (*r*_1_) of RM NPs, T_1_ relaxation times were measured at a field strength of 0.5 T at predetermined time points for RM NPs that were synthesized using different amounts of rutin ligand (RM-50 mg, RM-100 mg, RM-150 mg) in a mildly acidic environment (pH 6.6). The *r*_1_ was determined as the slope of the linear fit of 1/T_1_ relaxation time versus Mn^2+^ concentration. The results indicated that the *r*_1_ of RM NPs exhibited time-dependent behavior under acidic conditions (pH 6.6).

To confirm the pH-dependent response characteristics, RM NPs synthesized with 150 mg rutin ligand (at a fixed Mn^2+^ concentration of 0.3 mM) were dispersed in PBS buffers with a range of pH values (4.0, 5.0, 6.6, and 7.4). T_1_ relaxation times were measured at a field strength of 3.0 T. The results indicated a significant decrease in T_1_ relaxation times as the pH decreased, confirming the pH-dependent relaxivity response.

To evaluate the pH-responsive capability within a cellular context, the material was examined using two tumor cell lines (4T1 murine breast cancer cells and McA-RH7777 rat hepatoma cells) and two normal cell lines (RAW 264.7 macrophages and hTERT-HPNE human pancreatic ductal epithelial cells). The cells were cultured in 6 cm dishes. Upon reaching logarithmic growth, the culture medium was replaced with 4 mL of complete medium containing RM NPs (Mn^2+^ concentration: 100 μg/mL). The cells were incubated for 2 h, washed three times with PBS, trypsinized, and counted. An equal number of each cell type (1 × 10^6^ cells) was pelleted and embedded in 1 mL of 8% (w/v) agarose gel for immobilization. T_1_ relaxation times of the fixed cell pellets were measured at 3.0 T (n = 3). Following MRI, aliquots of the cell suspensions were digested overnight with concentrated nitric acid (75%). After sample weighing and dilution, the intracellular Mn concentration was quantified using AAS.

- 1. **Biosafety evaluation**
     1. ***In Vitro* cytotoxicity of RM NPs**

The cytotoxicity of RM NPs was evaluated using murine macrophage RAW 264.7 cells and murine breast cancer 4T1 cells via the CCK8 assay. In brief, the wells were designated as blank (culture medium only), control (culture medium + cells), and experimental (culture medium + RM NPs + cells) (n = 3 per group). RAW 264.7 and 4T1 cells were seeded into 96-well plates at a density of 10,000 cells per well and incubated for 24 h. Subsequently, RM NPs at various rutin concentrations (20, 110, 180, 230, 270, 300, 330 μmol/L) were added to the cells and coincubated for 24 h. After removing the medium and washing the cells thrice with PBS, fresh medium containing 10% CCK8 reagent was added, followed by incubation in the dark for 30 min. The OD of each well was measured at 450 nm using a microplate reader. To determine the half-maximal inhibitory concentration (IC_50_) for 4T1 cells, cell viability was first calculated as: Viability (%) = [(OD experiment – OD blank) / (OD control – OD blank)] × 100%. A dose-response curve was then generated by plotting viability against the logarithm of rutin concentration. The IC_50_ value was obtained by fitting the data to a logistic (sigmoidal) model using nonlinear regression analysis in Origin software. The reported IC_50_ of 133.52 ± 10.47 µg/mL (in terms of rutin) represents the mean ± standard deviation (SD) from three independent experiments.

- - 1. **Hemolysis assay of RM NPs**

Red blood cells (RBCs) were purified from 1 mL of whole rat blood by dilution with 2 mL of saline, followed by centrifugation (2000 rpm, 5 min). This washing step was repeated 3–4 times until the supernatant was clear. The RBC pellet was resuspended in saline at twice its volume to obtain a diluted RBC suspension. The test groups included: negative control (supernatant from centrifuged RBC suspension), positive control (supernatant from RBC suspension lysed with pure water), and experimental groups (supernatant from RBC suspension incubated with RM NPs). The experimental groups contained RMNPs at rutin concentrations of 20, 70, 110, 180, and 450 µM. The samples were adjusted to 1 mL final volume in Eppendorf tubes, incubated at 37°C for 1 h, and centrifuged at 2000 rpm for 5 min. The supernatant from each group (n = 4) was transferred (100 µL/well) to a 96-well plate, and the OD was measured at 545 nm using a microplate reader. The OD values for the RM NPs solutions with different rutin concentrations were also recorded. The OD values of the corresponding RM NPs solutions were deducted for each group of supernatants. The hemolysis rate was calculated using the following formula:

Hemolysis rate = $\frac{{OD}_{\mathrm{sample}}-{OD}_{\mathrm{control}}}{{OD}_{H_{2}O}-{OD}_{\mathrm{control}}}$× 100%

- - 1. **Histopathology and serum biochemistry**

Six female Balb/c mice (18–22 g) were randomly divided into two groups (n = 3 per group): experimental group received an intravenous injection of RM NPs (0.1 mmol Mn/kg), whereas control group received saline. After 10 days, blood samples were collected via the retro-orbital plexus and centrifuged at 2000 rpm for 5 min to obtain serum. Serum levels of liver function markers (alanine aminotransferase [ALT], aspartate aminotransferase [AST]), kidney function markers (creatinine [CREA], blood urea nitrogen [UREA]), serum albumin (ALB), and total protein were measured using a serum biochemistry analyzer. The mice were then euthanized, and the major organs (heart, liver, spleen, lungs, and kidneys) were harvested, fixed in formalin solution, processed for hematoxylin and eosin (H&E) staining, and subjected to histopathological examination.

- 1. ***In Vitro* apoptosis assay**

For the apoptosis assay, the following groups were used: RM NP, rutin, and blank control (n = 4 per group). The RM NP group contained rutin concentrations of 130 µM and 260 µM, which were designated as Treated-1 and Treated-2, respectively. The rutin group contained 260 µM rutin (matching Treated-2). When 4T1 murine breast cancer cells reached the logarithmic growth phase, the culture medium from each group was aspirated into centrifuge tubes. Adherent cells were washed once with PBS and trypsinized. The original collected medium was added back, and the cells were gently resuspended, transferred to centrifuge tubes, and centrifuged at 1000 rpm for 5 min. The supernatant was discarded, and the pellet was collected, gently resuspended in PBS, and counted. Cell suspensions containing 5–10 × 10⁴ cells were transferred to centrifuge tubes and centrifuged at 1000 rpm for 5 min. The supernatant was discarded. The cells were gently resuspended in 195 µL Annexin V-FITC binding buffer. Then, 5 µL Annexin V-FITC was added and mixed gently. Subsequently, 10 µL propidium iodide staining solution was added, mixed gently, and incubated at room temperature (20 °C–25 °C) in the dark for 10–20 min. The samples were analyzed via flow cytometry.

- 1. **Western blot analysis**

4T1 cells were coincubated with RM NPs (260 µM rutin equivalent; RM NPs group) or free rutin (260 µM; Rutin group) for 24 h, whereas untreated 4T1 cells served as the blank control. Proteins were extracted from all three groups, diluted with PBS, and the protein concentration was measured using a BCA assay kit. The samples were mixed with loading buffer and denatured by boiling for 10 min. The samples, along with markers, were loaded into wells for sodium dodecyl sulfate-polyacrylamide gel electrophoresis (SDS-PAGE). The proteins were then transferred to membranes using a transfer apparatus. The membranes were blocked with rapid blocking buffer, incubated with primary antibodies overnight, followed by incubation with secondary antibodies for 1–2 h. Protein bands were visualized using an enhanced chemiluminescence (ECL) detection system. Band intensity was quantified using ImageJ software to calculate the relative expression of the target proteins.

- 1. **Experimental animals**

Female Balb/c mice (6–8 weeks old, 18–22 g) were used for the animal experiments. To model orthotopic breast cancer, 5 × 10⁶ 4T1 cells suspended in 100 µL PBS were injected subcutaneously into the left axilla. After 4–5 days, when tumors reached approximately 50 mm³, the mice were used for imaging and therapy. To establish a breast cancer liver metastasis model, 1 × 10⁶ 4T1 cells suspended in 50 µL PBS were injected into the spleen. After 4–5 days, the mice were used for imaging and therapy of liver metastases. All animal experiments were approved by the Animal Ethics Committee of North Sichuan Medical College.

- 1. ***In Vivo* MRI of subcutaneous orthotopic breast tumors**

4T1 cells (5 × 10^6^) were implanted subcutaneously into the left axilla of female Balb/c mice (6–8 weeks old, 18–22 g). When tumors reached approximately 50 mm³, the mice were fasted for 24 h and divided into three groups (n = 3 per group). Groups I and II received intravenous injections of Gd-DTPA (a clinically used gadolinium-based contrast agent, 0.1 mmol Gd/kg body weight) and RMNPs (0.1 mmol Mn/kg body weight), respectively. Images were acquired at various time points using a 3.0-T MRI scanner. Tumor signal intensity (SI) was measured using DICOM Viewer software for subsequent analysis. To confirm the *in vivo* targeting capability of RM NPs, group III mice were orally administered 20% glucose solution, and 15–20 min later, RM NPs were injected intravenously (0.1 mmol Mn/kg). Tumor SI was acquired and analyzed. Imaging was performed using a conventional spin-echo sequence with the following parameters: TE = 9 ms, TR = 513 ms, slice thickness = 0.4 mm, spacing between slices = 2 mm, FOV = 80 mm, number of averages = 2, and flip angle = 90°.

**Supplementary Figures**


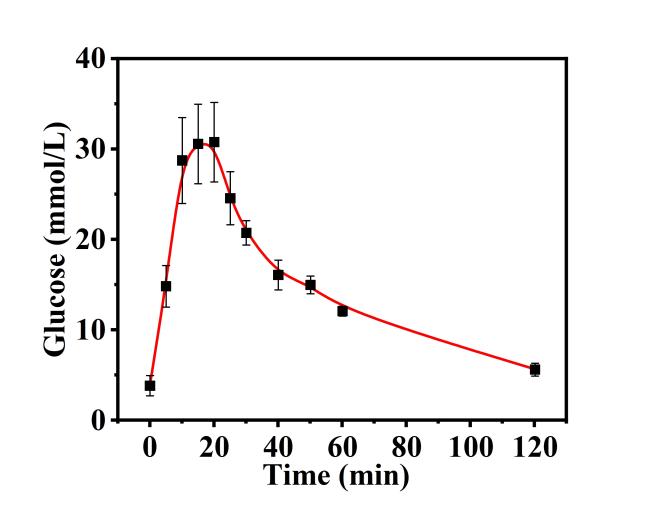


**Figure S1**. Changes in blood glucose levels in mice after glucose gavage. Mice were pre‑treated by oral gavage with a 20% glucose solution at a dose of 10 mL/kg. Blood glucose levels were monitored using a glucometer at the indicated time points (mean ± SD, n = 4).


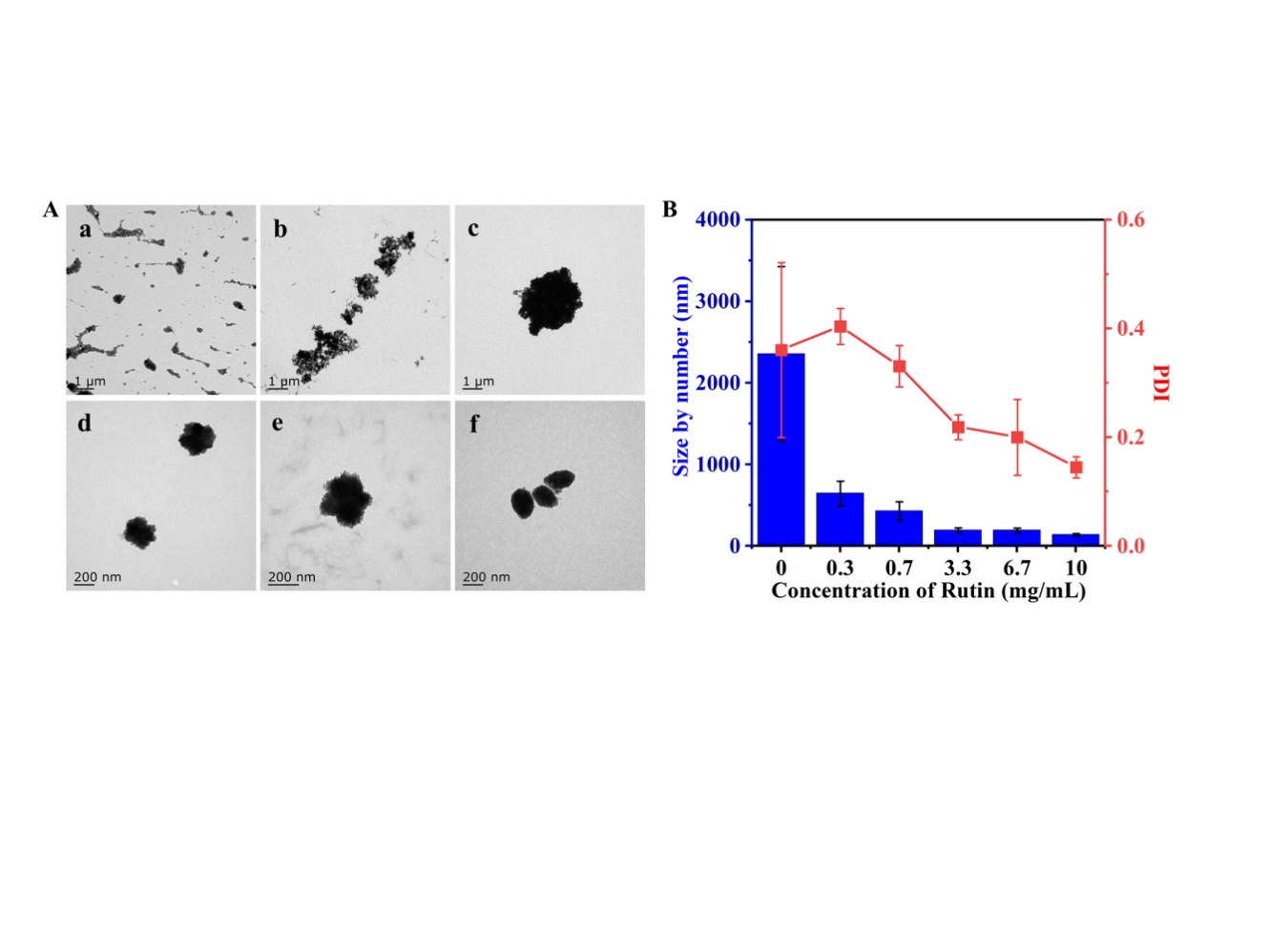


**Figure S2.** Characterization of RM NPs prepared with different rutin ligand concentrations. (**A**) TEM images showing morphological changes (a–f: 0, 0.3, 0.7, 3.3, 6.7, and 10 mg/mL, respectively). (**B**) Corresponding hydrodynamic diameter and PDI measurements (mean ± SD, n = 3).


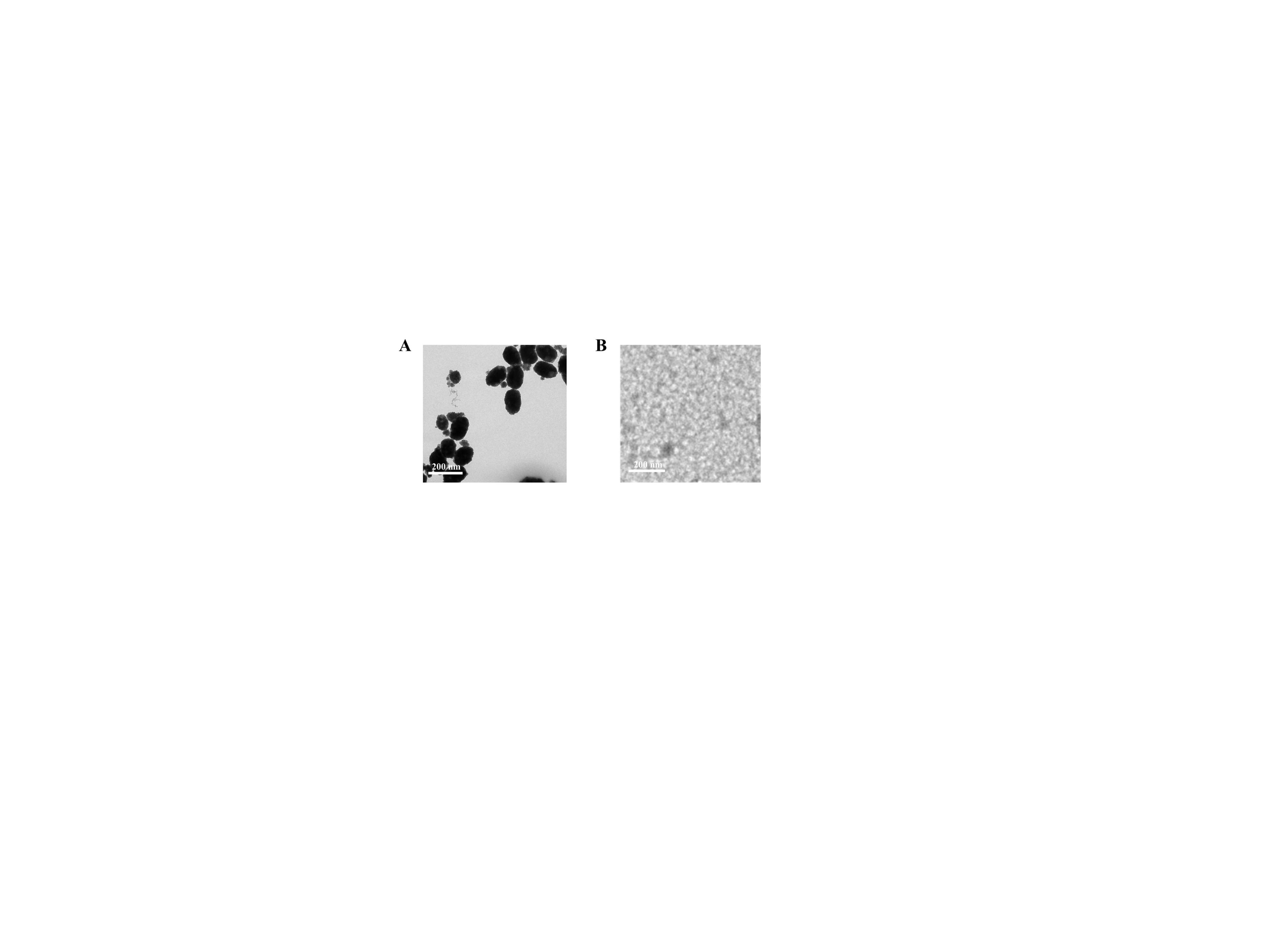


**Figure S3.** TEM images of RM NPs in different environments: (**A**) neutral environment and (**B**) weak acid environment.


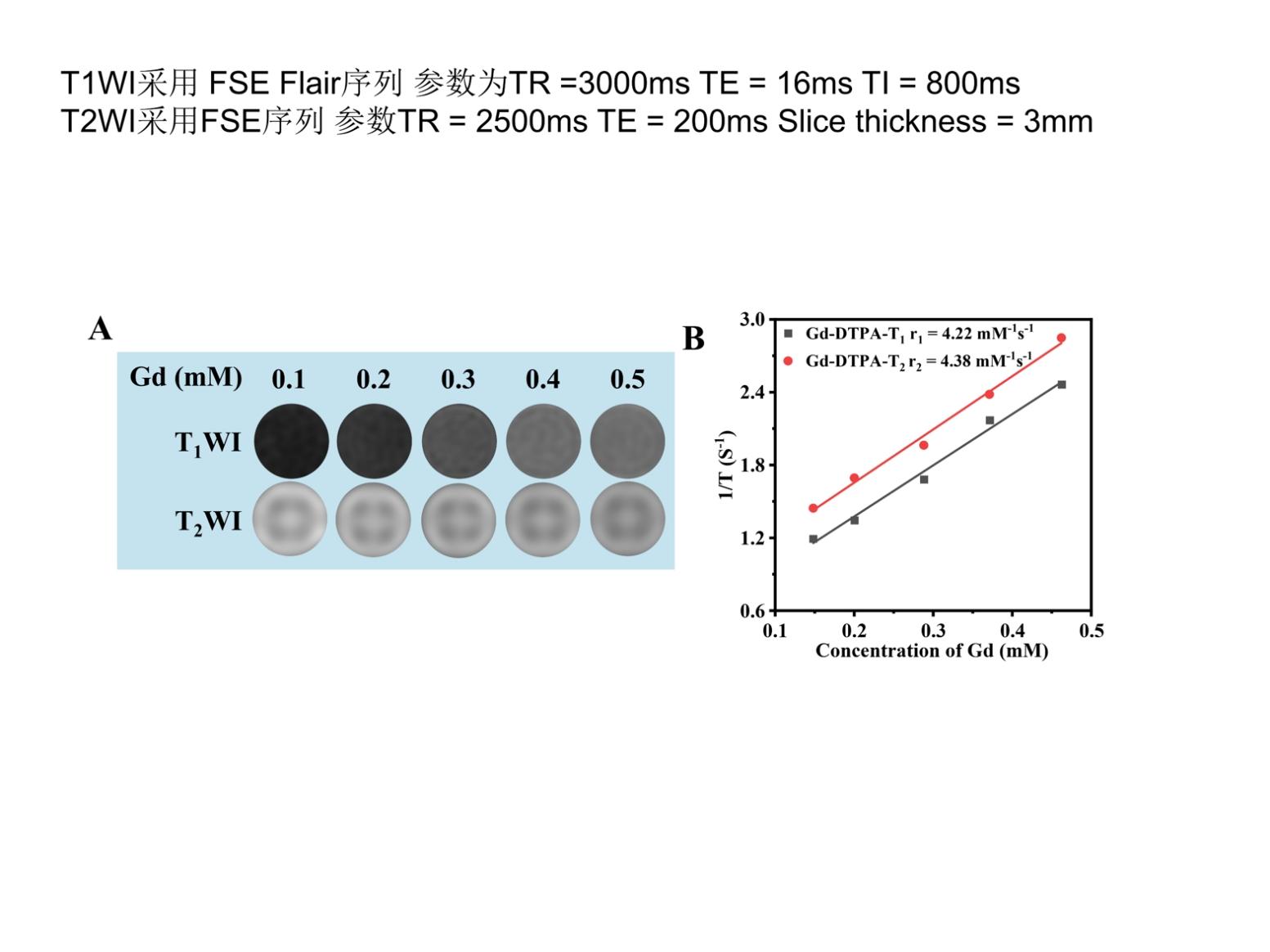


**Figure S4.** Evaluation of the relaxivity efficacy of Gd-DTPA at 3.0 T. (**A**) MR images of Gd-DTPA under T_1_WI and T_2_WI at 3.0 T. T_1_WI was acquired using the FSE flair sequence (TR = 3000 ms, TE = 16 ms, TI = 800 ms, slice thickness = 3 mm). T_2_WI was acquired using the FSE sequence (TR = 2500 ms, TE = 200 ms, slice thickness = 3 mm). (**B**) Assessment of *r*_1_ and *r*_2_ of Gd-DTPA. Relaxivity fitting was conducted using the FSE flair sequence and FSE sequence.


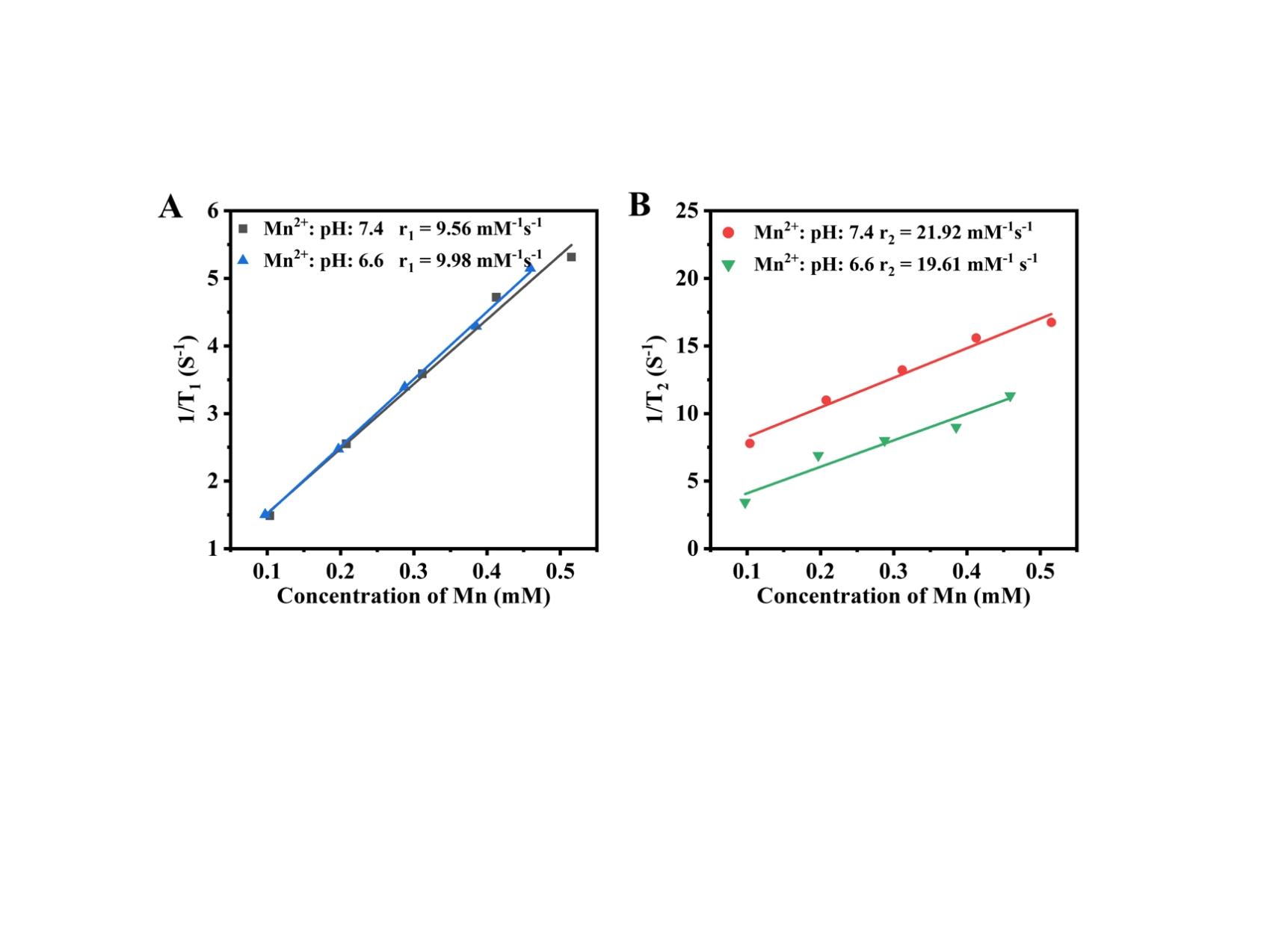


**Figure S5.** Relaxivity determination of Mn^2+^ in neutral aqueous solution and PBS (pH 6.6) at 3.0 T. (**A**) *r*_1_ was comparable between the aqueous solution and buffer. (**B**) *r*_2_ was also comparable under both conditions. *r*_1_ and *r*_2_ were fitted using the FSE flair and FSE sequences, respectively.


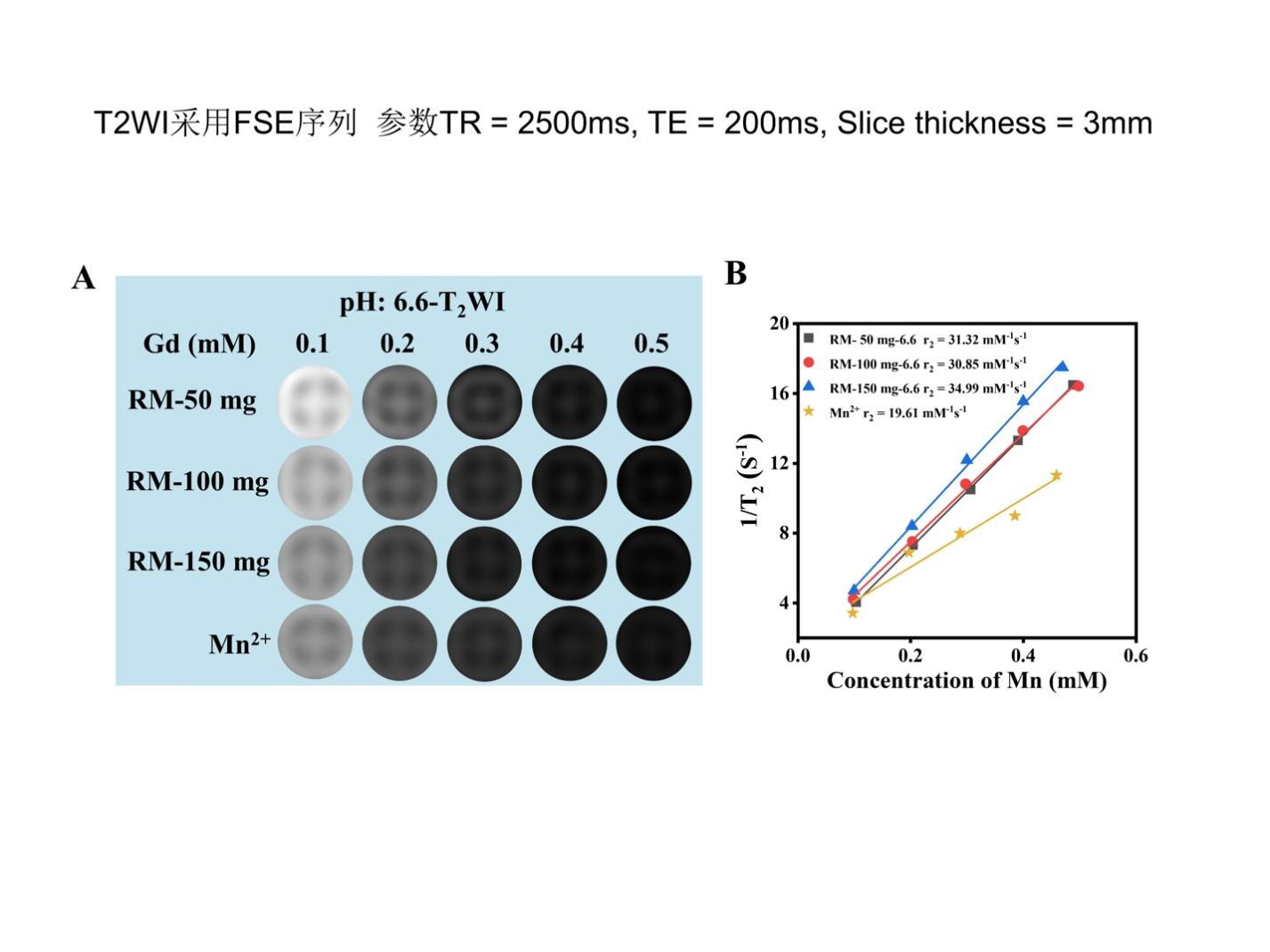


**Figure S6.** T_2_ relaxation efficacy evaluation of RM NPs and Mn^2+^ in PBS (pH 6.6) at 3.0 T. (**A**) T_2_WI MR images of RM NPs and Mn^2+^ acquired with the FSE T_2_WI sequence (TR = 2500 ms, TE = 200 ms, slice thickness = 3 mm). (**B**) *r*_2_ of three RM NPs with different ligand loadings and Mn^2+^.


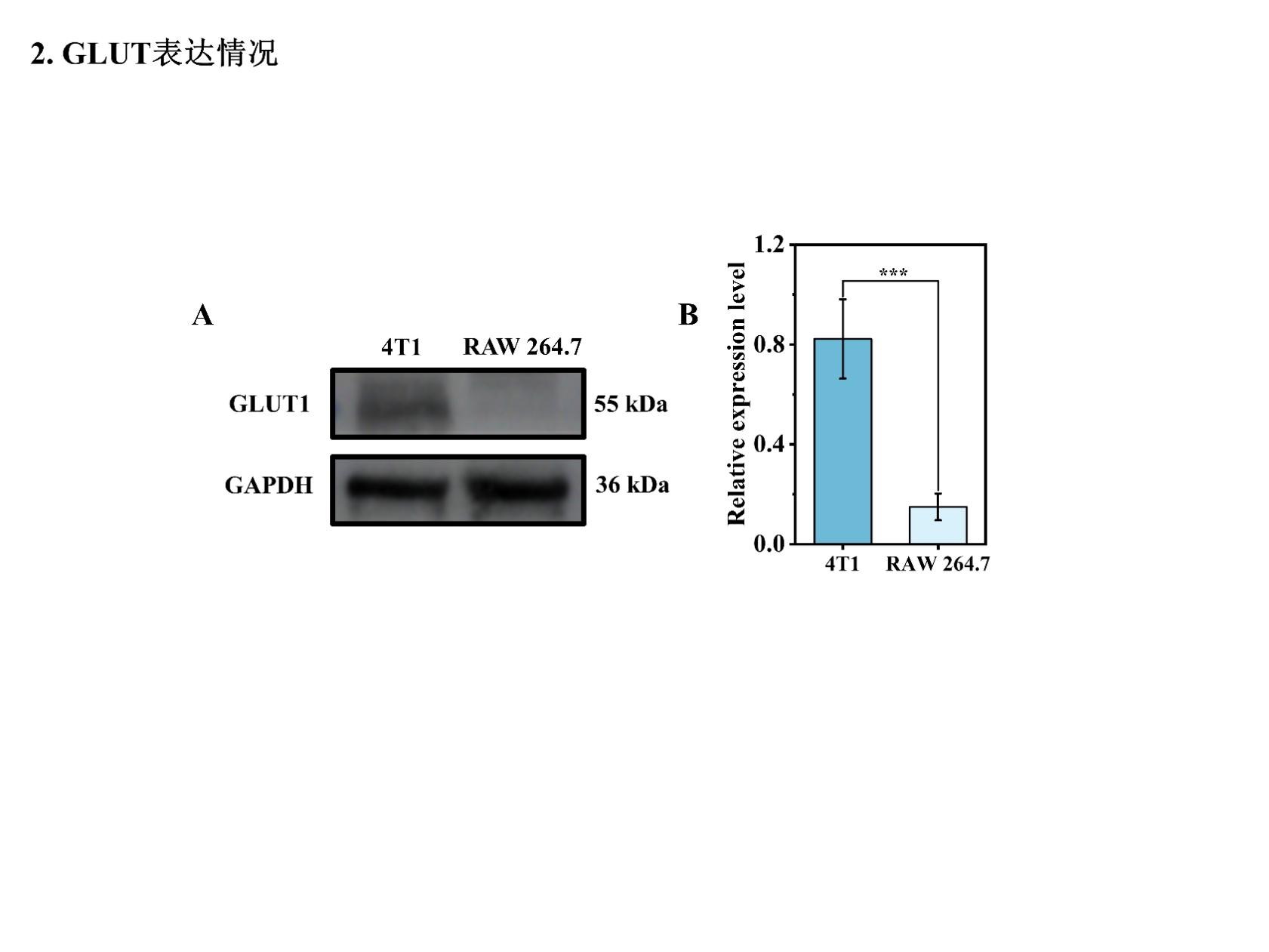


**Figure S7.** Glucose Receptor Expression. (**A**) shows the representative Western blot image, and (**B**) presents the relative expression levels of 4T1 and RAW 264.7 cells. n = 4, mean ± SD.


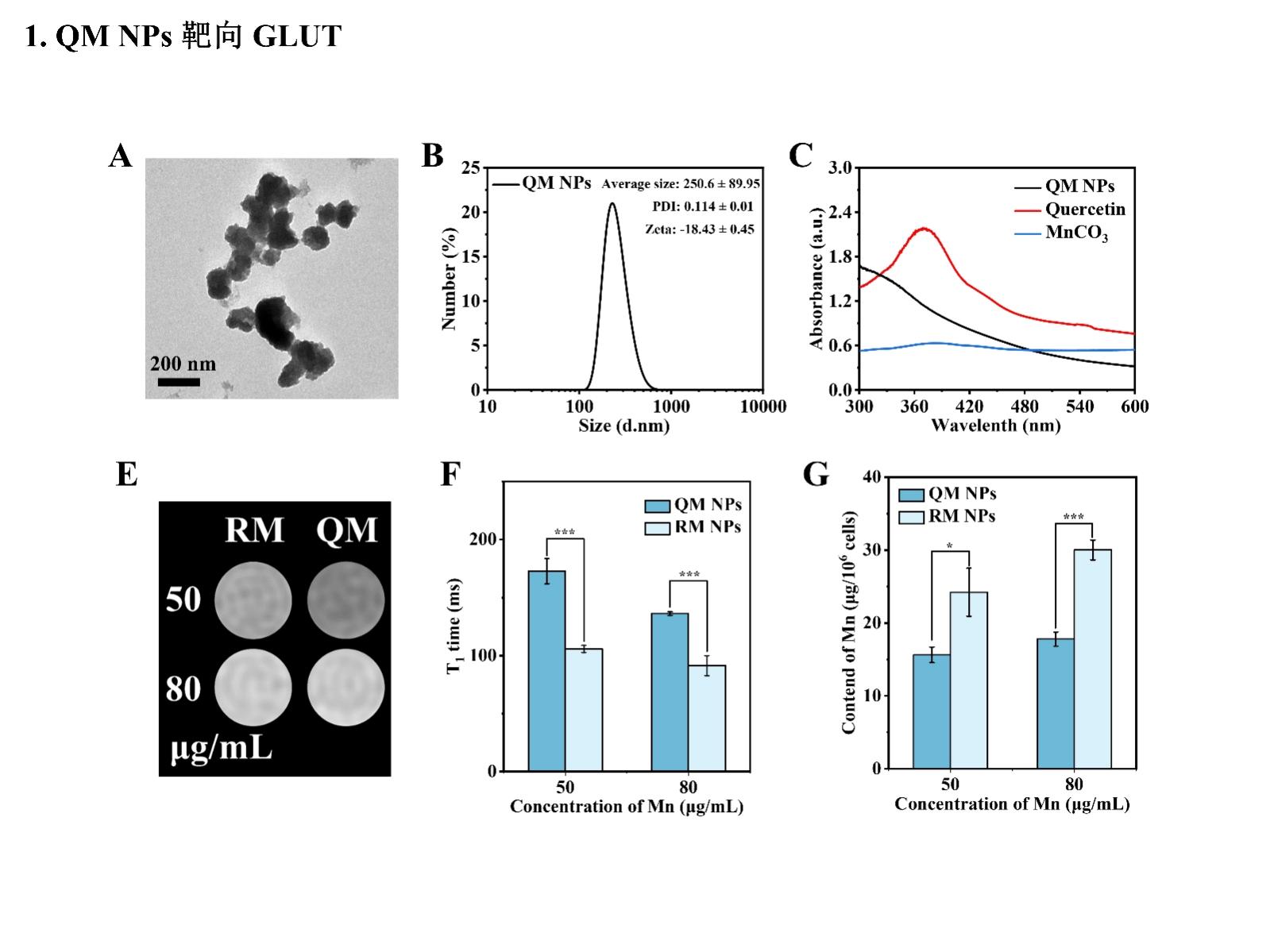


**Figure S8.** GLUT Targeting Validation. (**A**) and (**B**) show the morphology and hydrodynamic diameter of QM NPs, (**C**) is the UV spectrum of QM NPs confirming successful synthesis, and (**E**), (**F**), and (**G**) present the T_1_ values at 3.0 T and metal ion content after incubation of QM NPs and RM NPs with 4T1 cells. n = 3, mean ± SD.

**
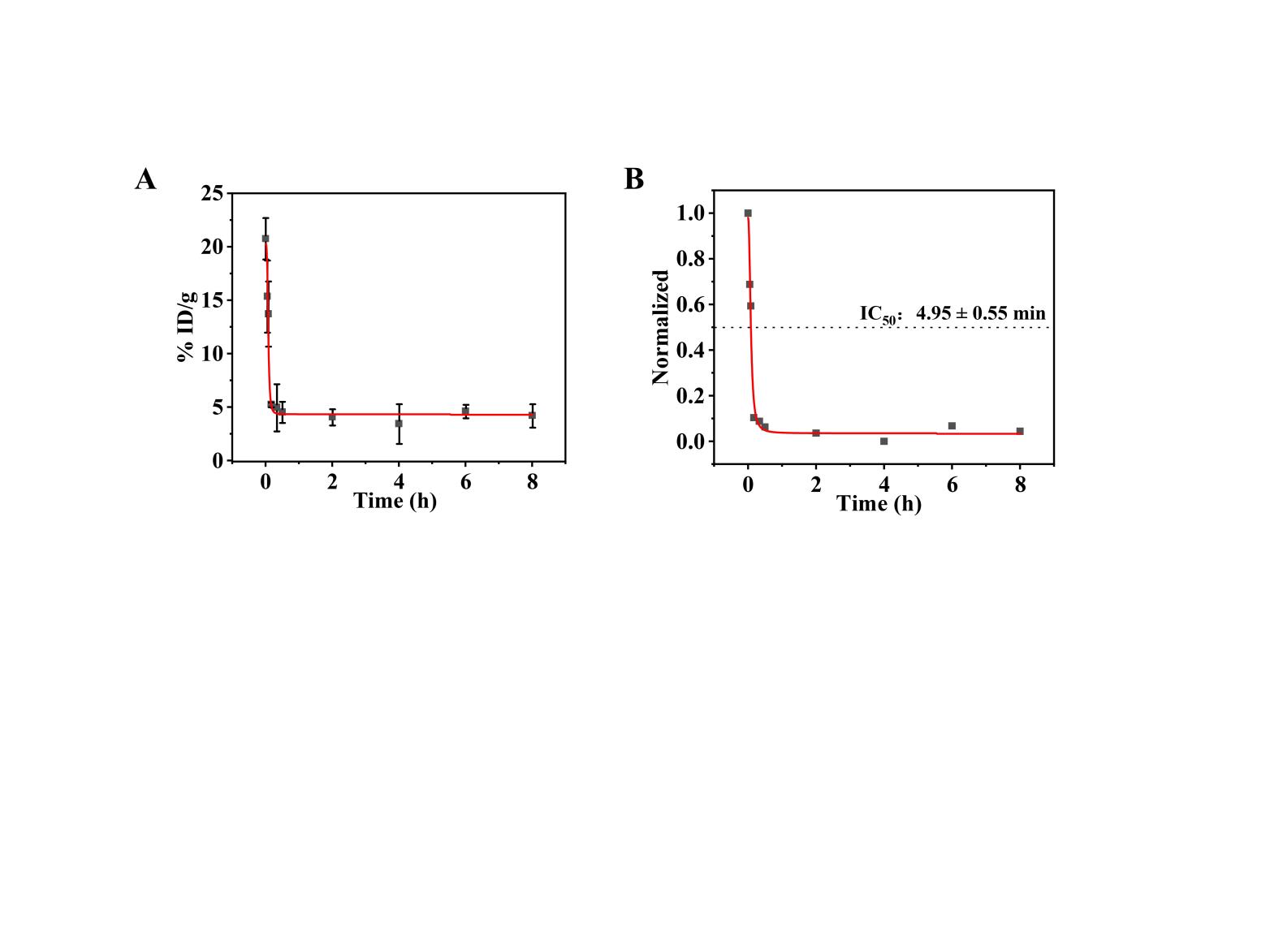
**

**Figure S9.** Blood circulation half-life of RM NPs. (**A**) After tail vein injection of RM NPs at a dose of 0.1 mmol/kg Mn^2+^, blood was collected via orbital sampling at different time points, followed by nitric acid digestion and measurement of the proportion of Mn^2+^. (**B**) To calculate the blood circulation half-life of RM NPs in mice, the data in (A) were normalized, yielding a half-life of 4.95 ± 0.55 min.


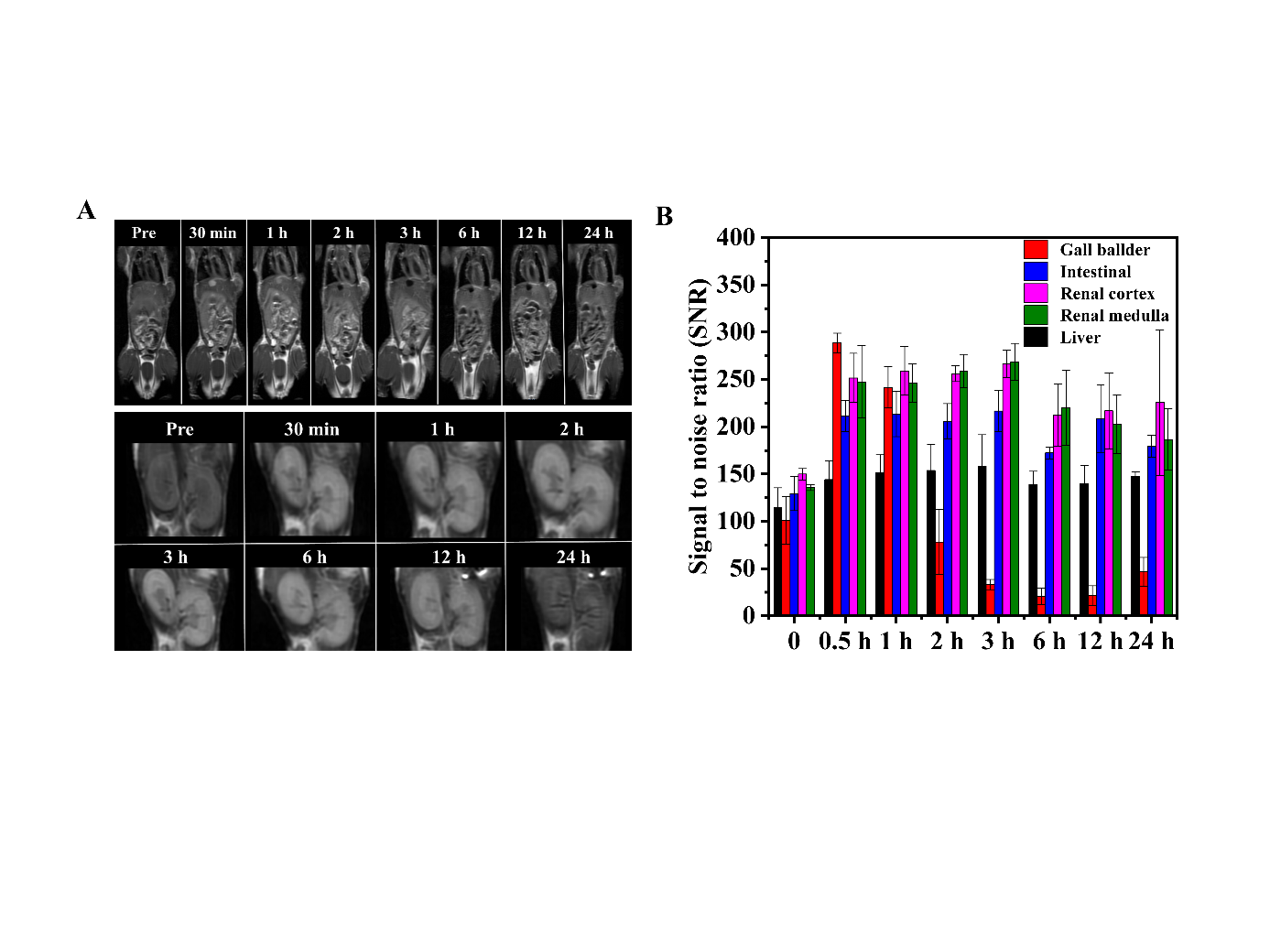


**Figure S10.** *In vivo* biodistribution of RM NPs monitored using MRI at 3.0 T. (**A**) T_1_WI before and 24 h post injection (0.05 mmol Mn/kg), (**B**) SNR quantification in the target organs (mean ± SD, n = 3).


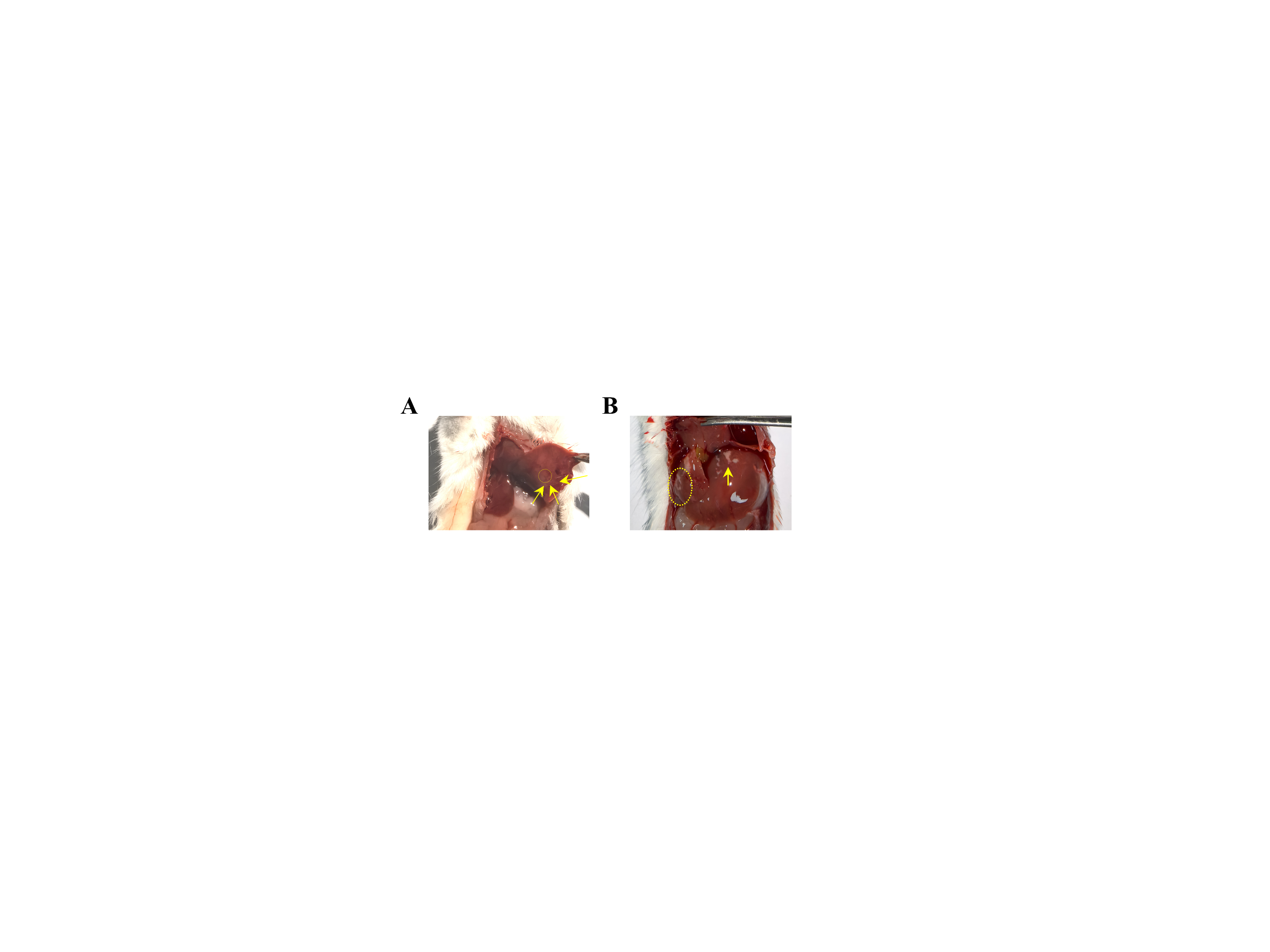


**Figure S11.** Abdominal dissection photographs of mice. (**A**) and (**B**) show the abdominal dissection photographs of mice in the experimental group (RM NPs) and the control group (Primovist), respectively.


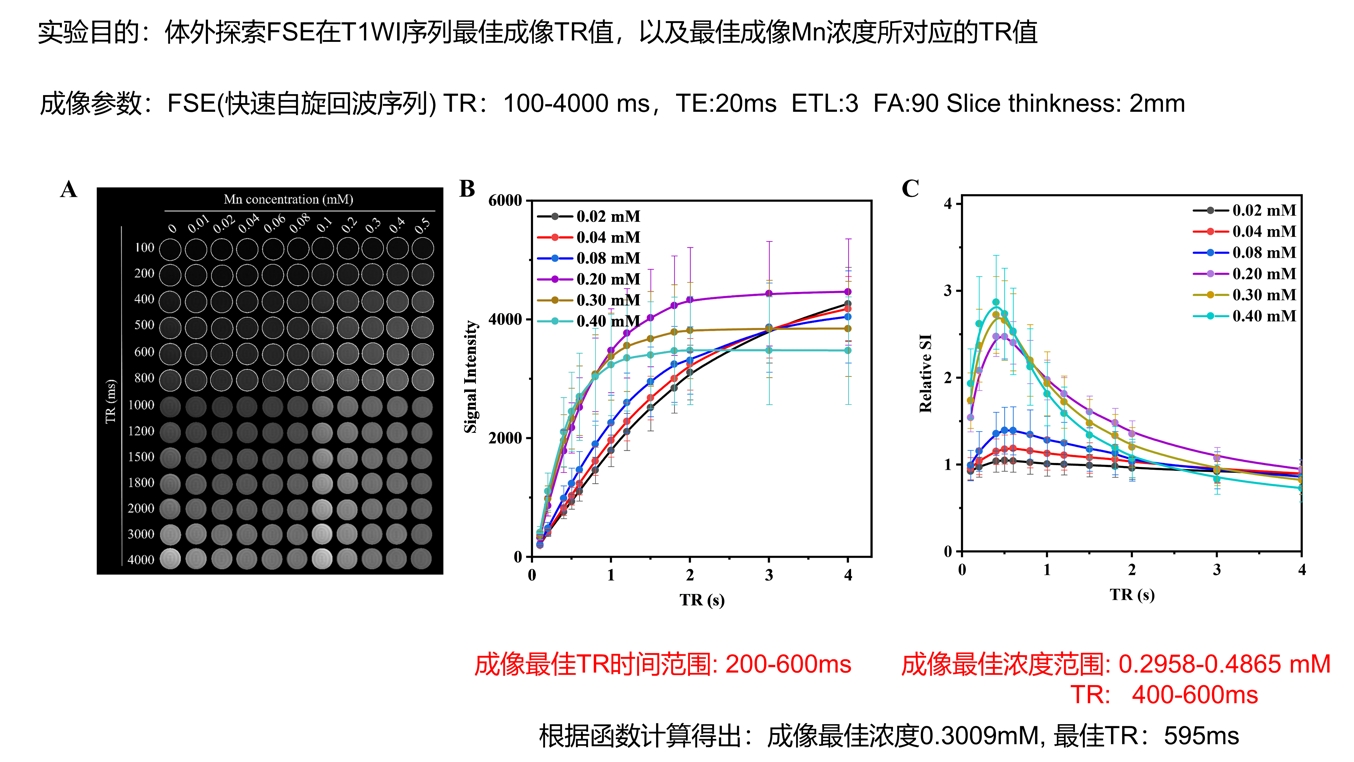


**Figure S12.** Evaluation of T_2_ shortening effect at 3.0 T. (**A**) With TE fixed at 20 ms, the changes in the MR signals of RM NPs at various Mn concentrations at pH 6.6 are shown as TR values increase. (**B**) With a fixed TE value of 20 ms and increasing TR values, the signal values corresponding to RM NPs with different Mn concentrations change as shown in the diagram. (**C**) The relative signal (relative SI) of RM NPs at different Mn concentrations as a function of TR prolongation (mean ± SD, n = 3). *Relative* *SI* = *SI* / *SI_0_*.The parameters are as follows: FSE, TR: 100–4000 ms, TE = 20 ms, ETL = 3, FA = 90°, and slice thickness = 2 mm


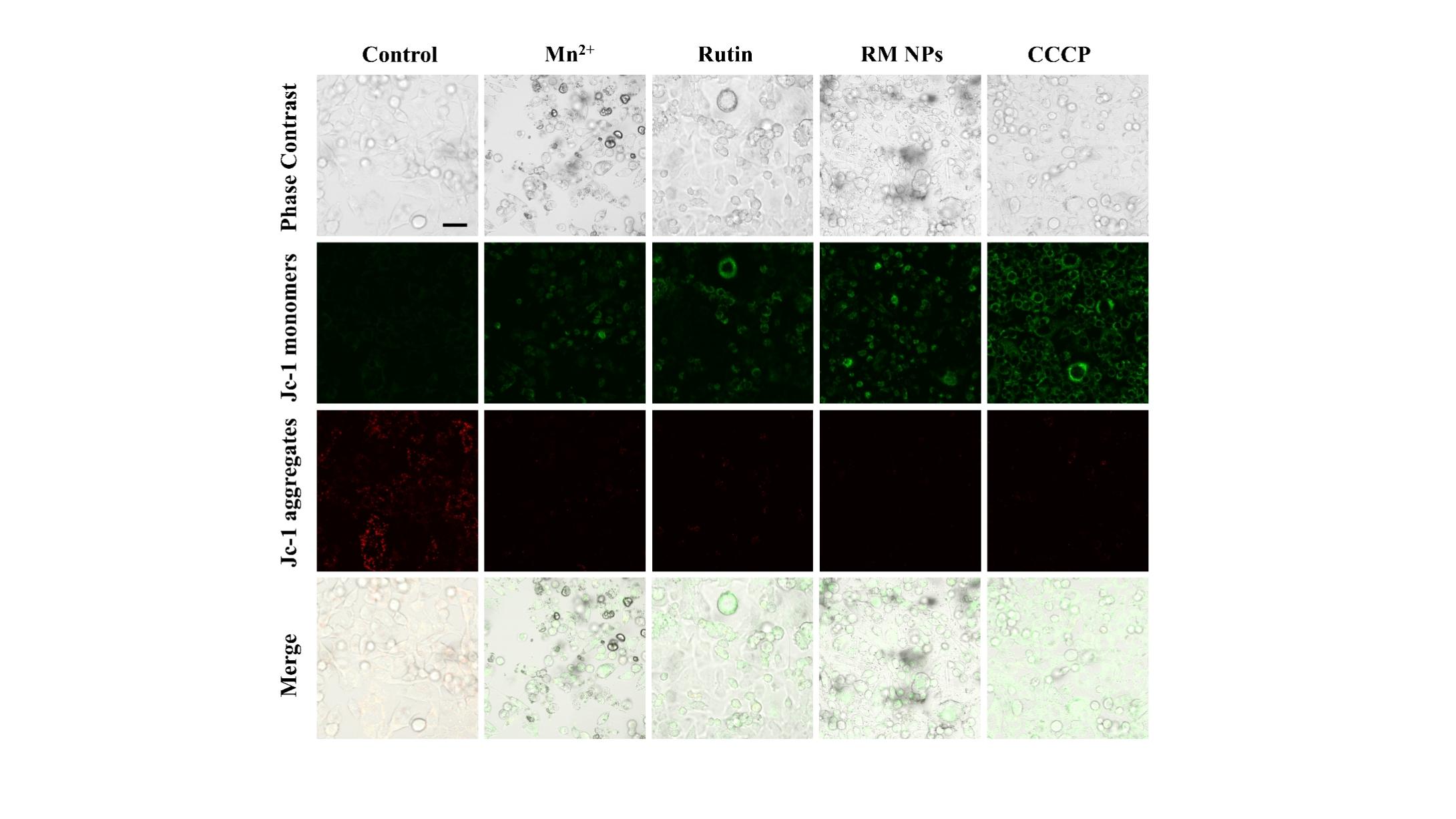


**Figure S13.** Apoptosis of 4T1 cells was observed by confocal microscopy using JC-1 staining. 4T1 cells were treated with the IC50 concentration in all three treatment groups, with Control and CCCP serving as the negative and positive control groups, respectively (Scale bar=40 µm).

**
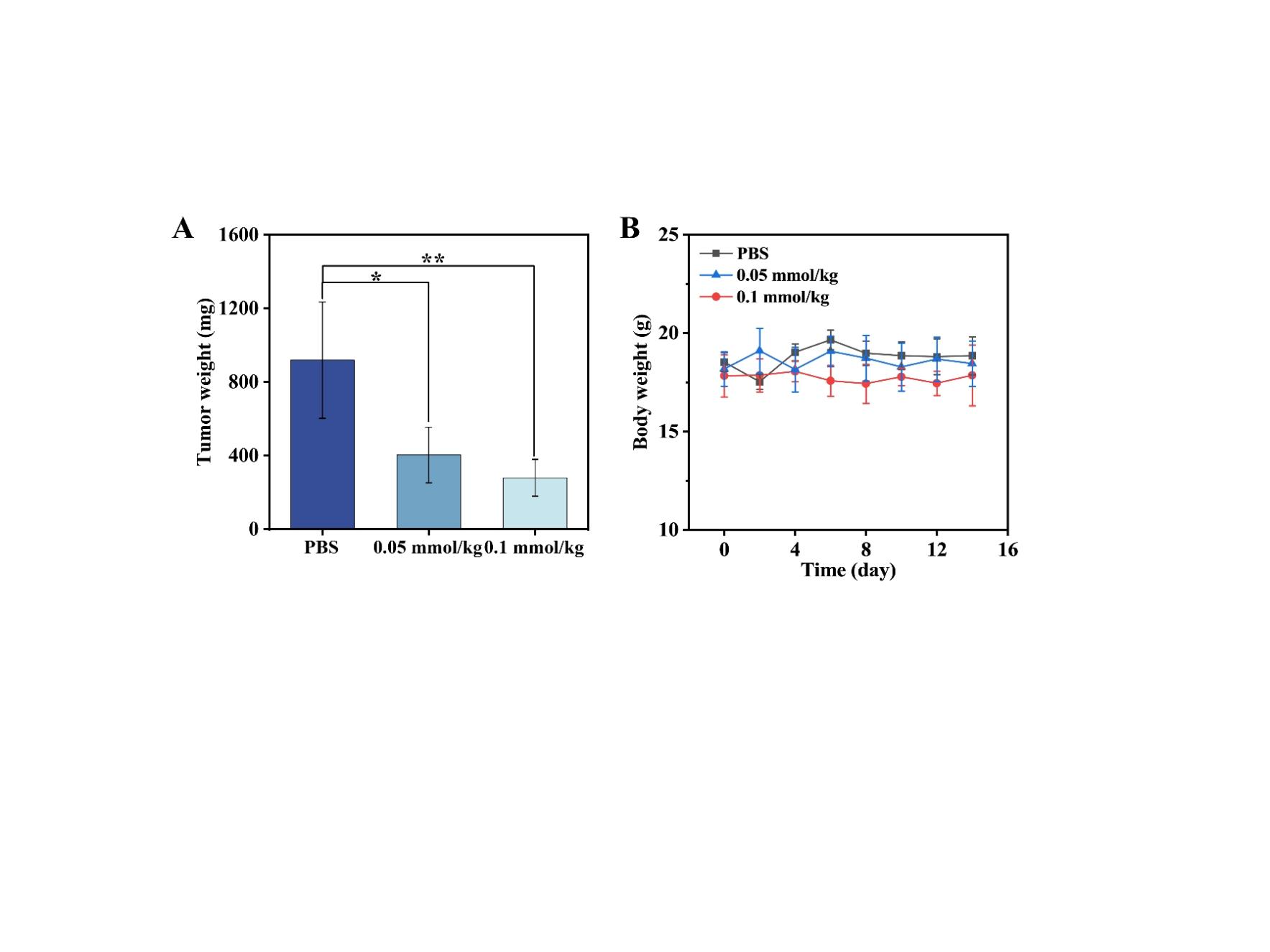
**

**Figure S14.** Post-treatment tumor mass and murine body weight dynamics. (**A**) *Ex vivo* tumor weight in different groups, further confirming the therapeutic effect of RM NPs. (**B**) Changes in mouse body weight during the 14-day treatment period (mean ± SD, n = 4).


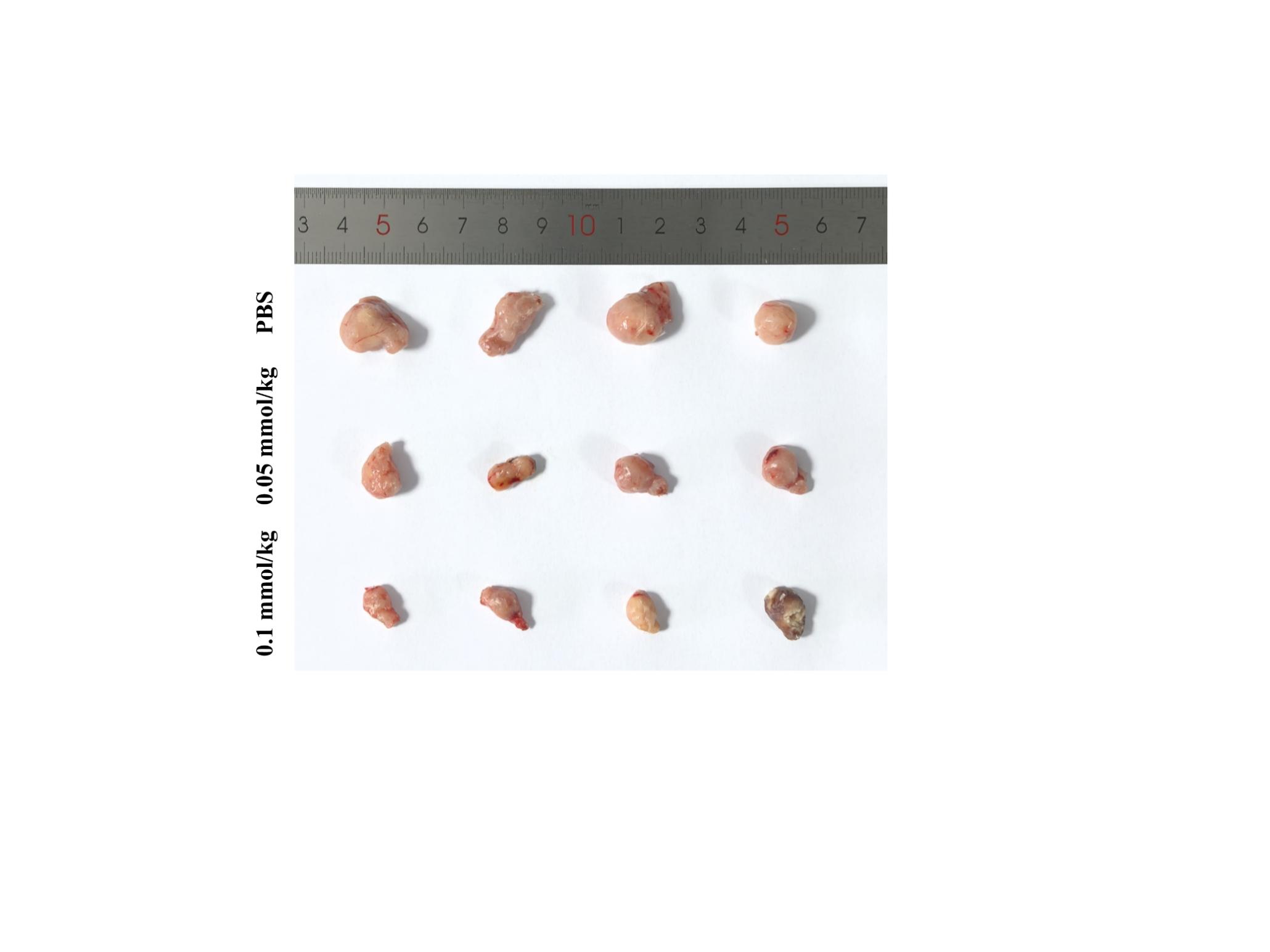


**Figure S15.** Subcutaneous breast tumor dissection images *ex vivo.*


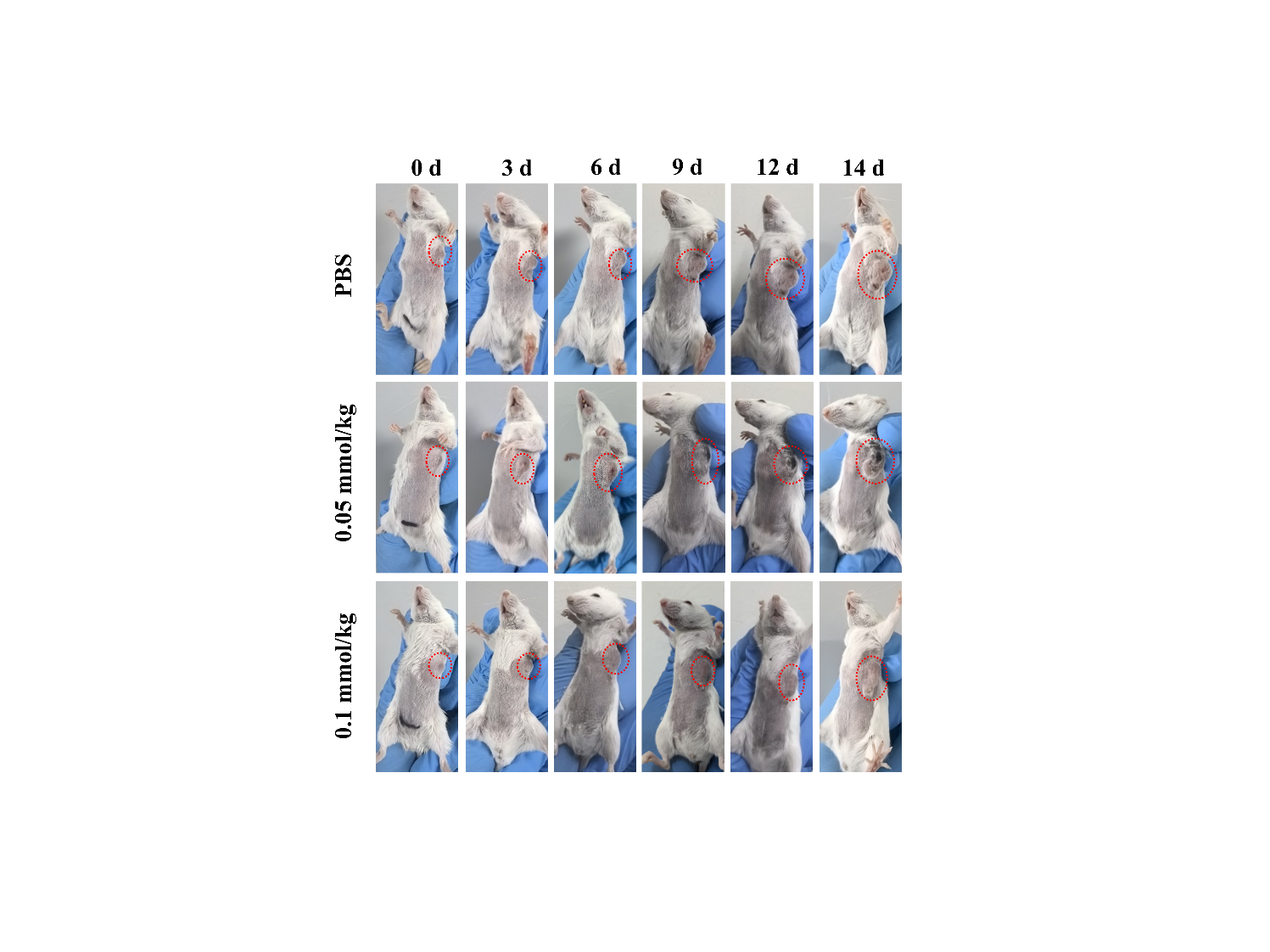


**Figure S16.** Real-time *in vivo* monitoring of subcutaneous breast tumors in the treatment and control groups over 14 days.


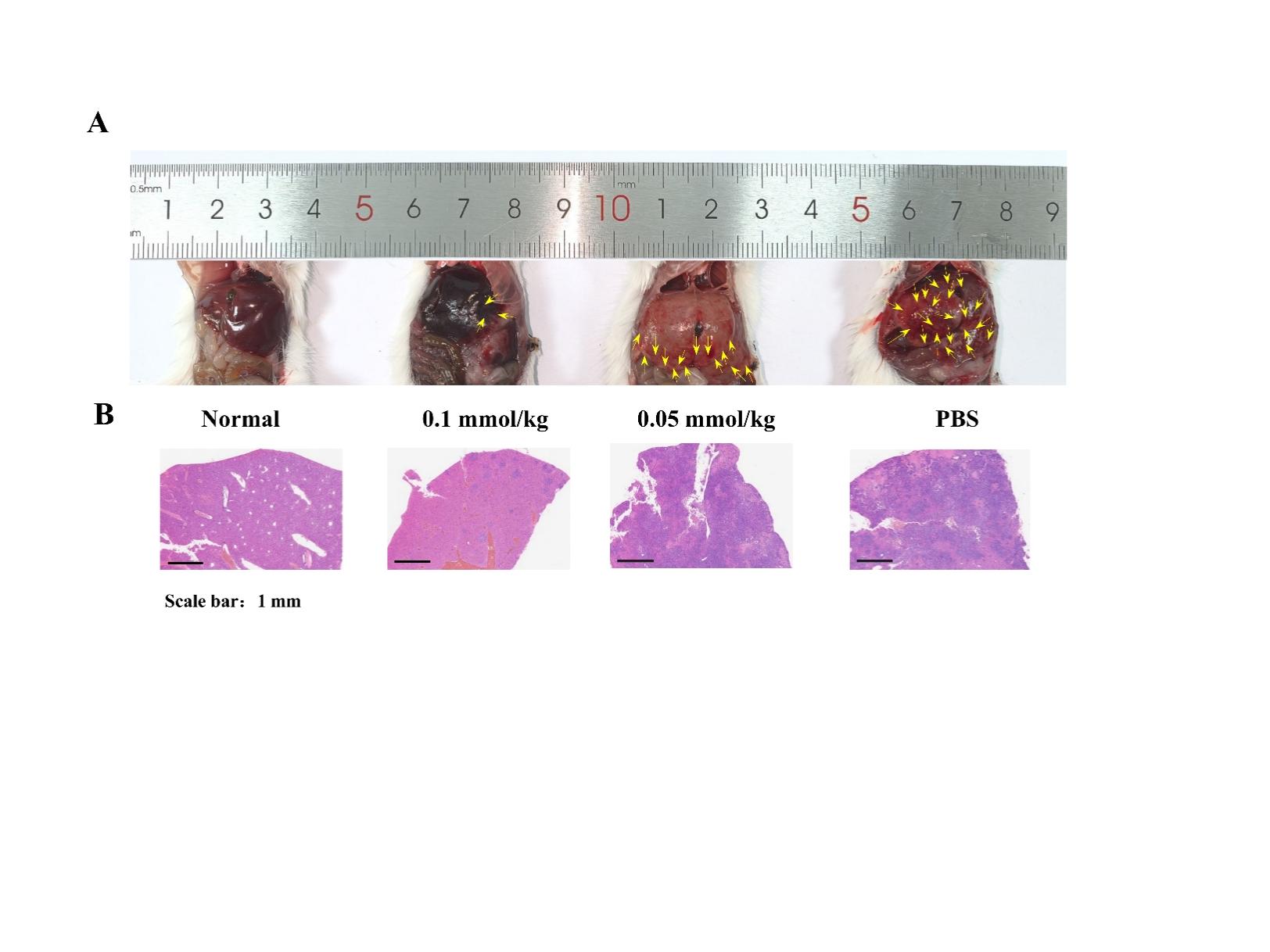


**Figure S17.** Therapeutic efficacy of RM NPs on breast cancer liver metastases. (**A**) Gross liver anatomy and (**B**) H&E staining (yellow arrows indicate metastatic foci).

**B**

**A**


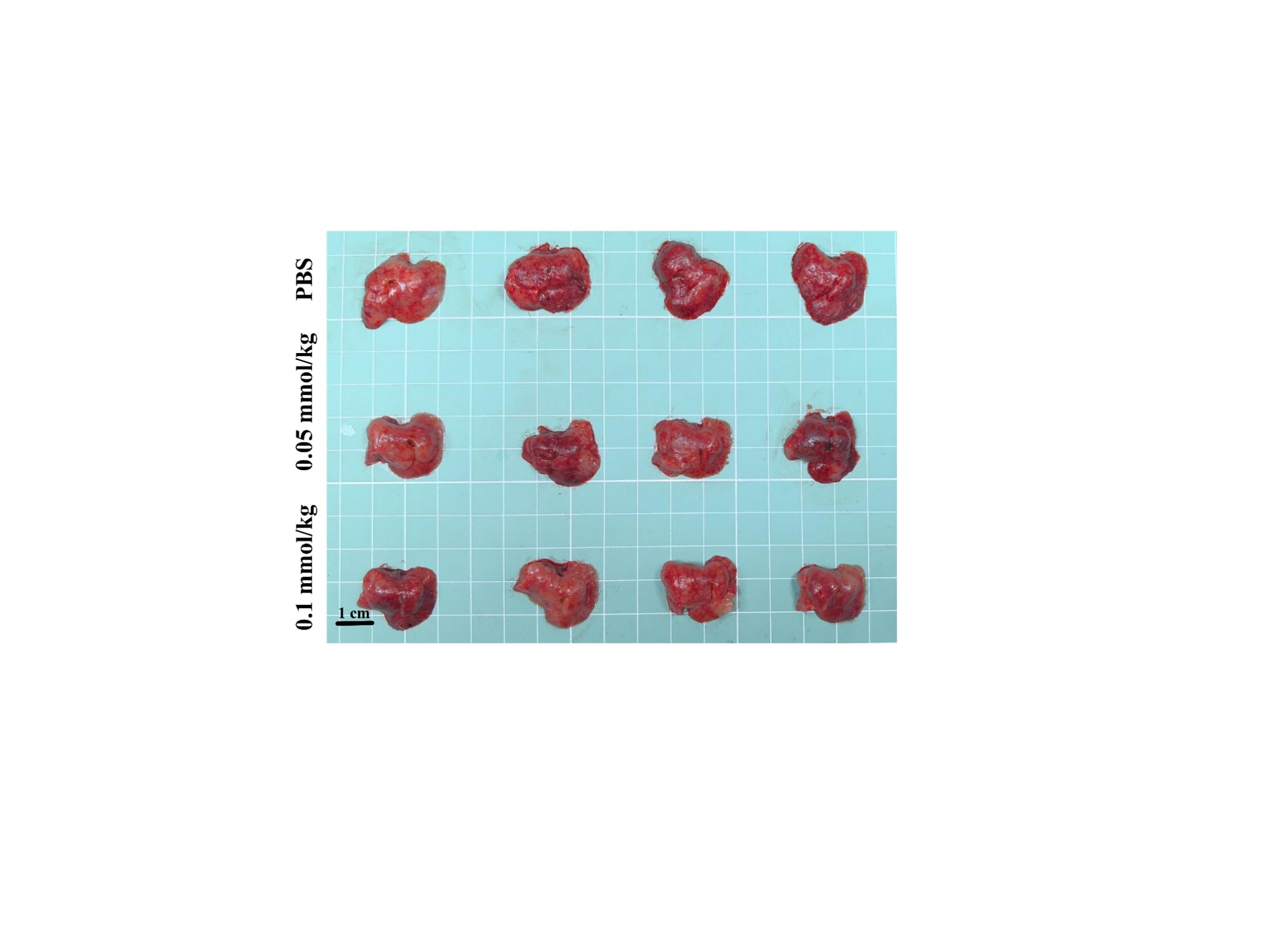


**Figure S18.** *Ex vivo* liver dissection images of mice from three treatment groups post therapy.


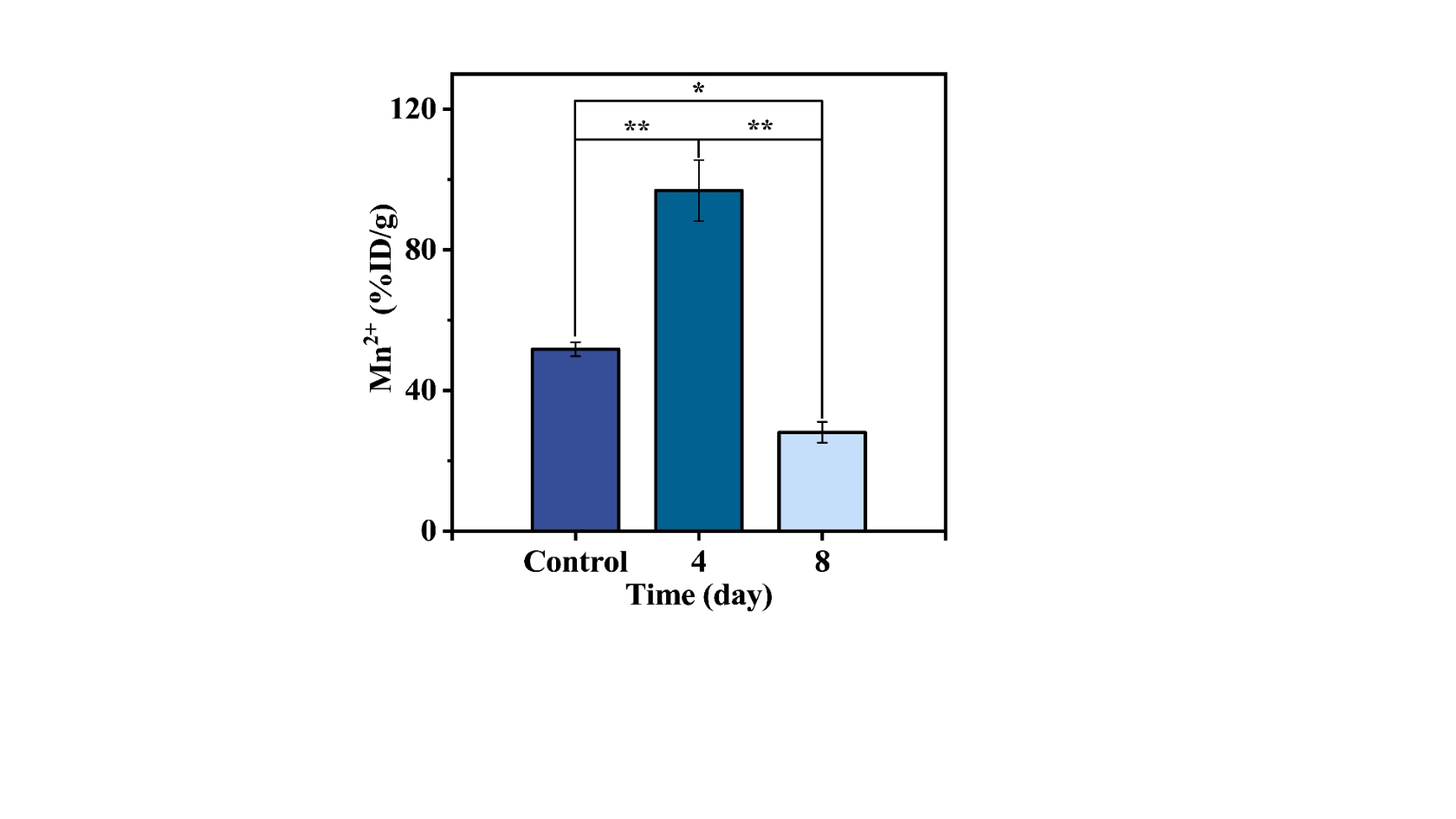


**Figure S19.** Hepatic manganese ion levels measured using ICP-MS in nonmodeled, 4 day postmodeling, and 8 day postmodeling groups. Except for the control group, all groups received RM NPs (0.1 mmol Mn/kg) and were sacrificed 2 h later for liver ICP-MS analysis.


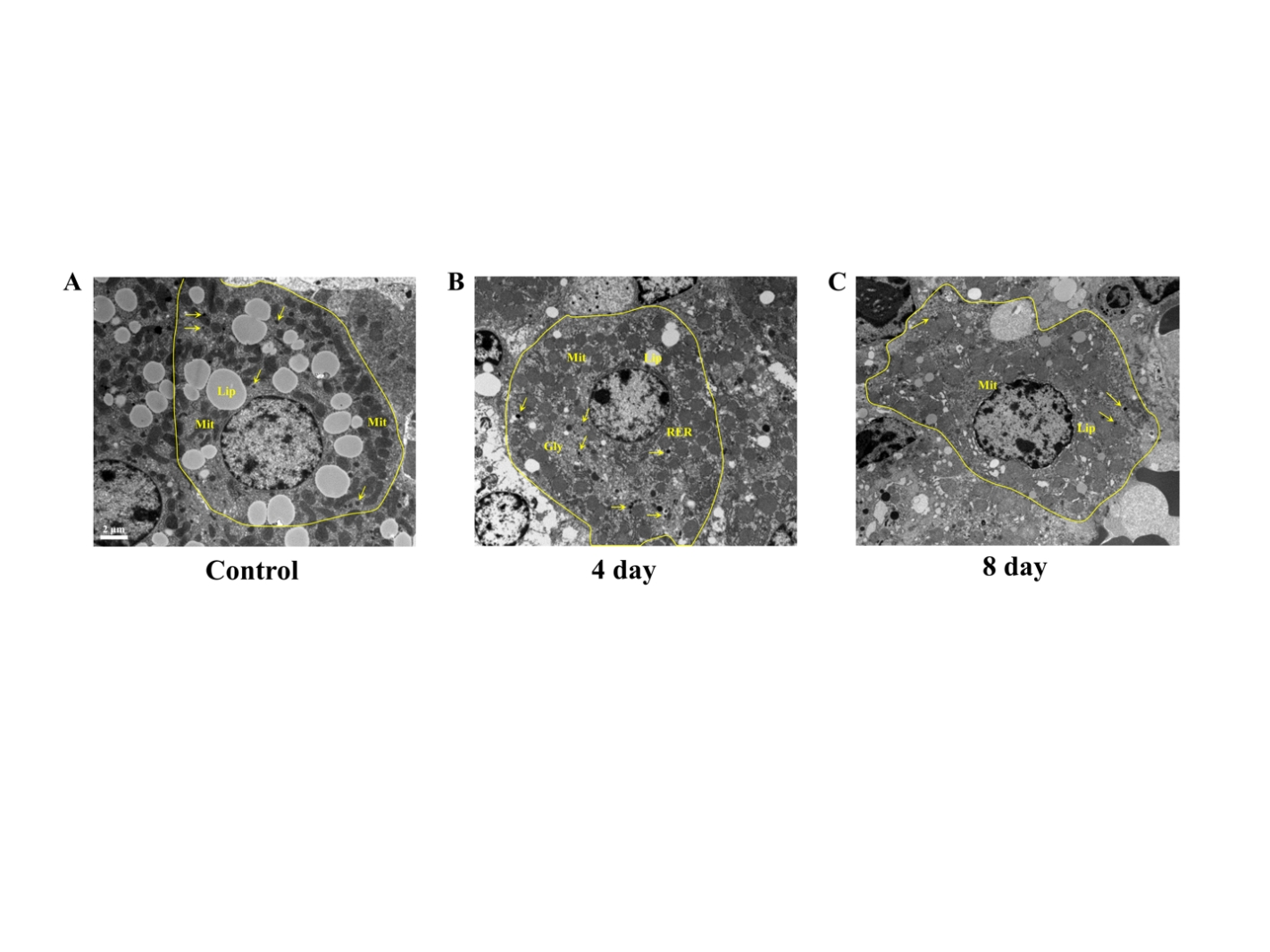


**Figure S20.** Bio-TEM visualization of RM NP internalization in hepatic cells at 2 h post injection.


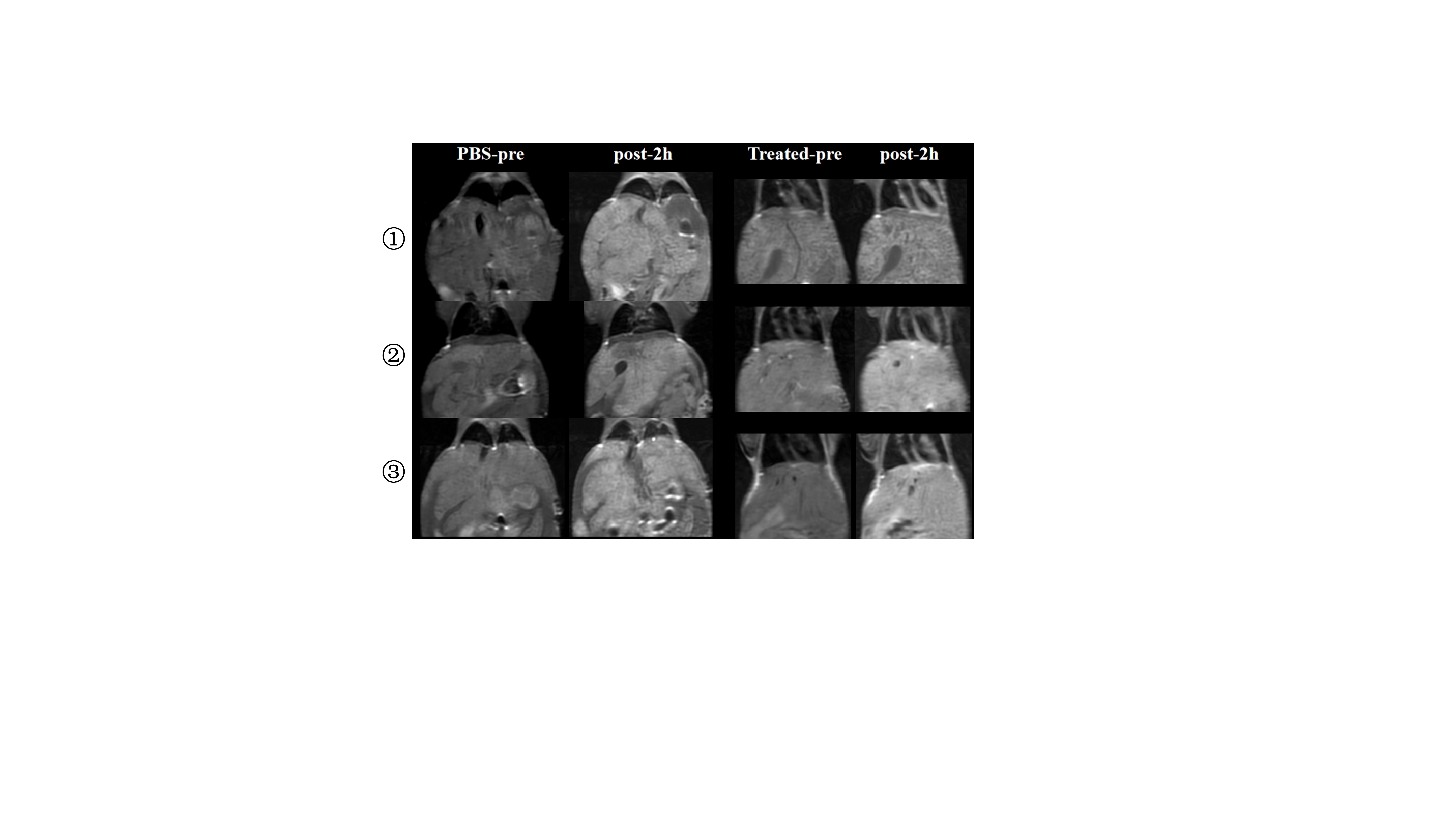


**Figure S21.** Assessment of prognosis improvement in liver metastases treated with RM NPs. The figure shows the comparison of the PBS control groups with the RM NP-treated groups treated with 0.05 mmol/kg RM NPs twice within 12 days (each group n = 3). Injection and post-2 h injection abdominal T_1_-weighted MRI scans are shown. The parameters for abdominal T_1_-weighted were as follows: TE = 17 ms, TR = 480 ms, slice thickness = 0.4 mm, spacing between slices = 0.8 mm, ETL = 3, FOV = 100 mm, number of averages = 2, and flip angle = 142°.


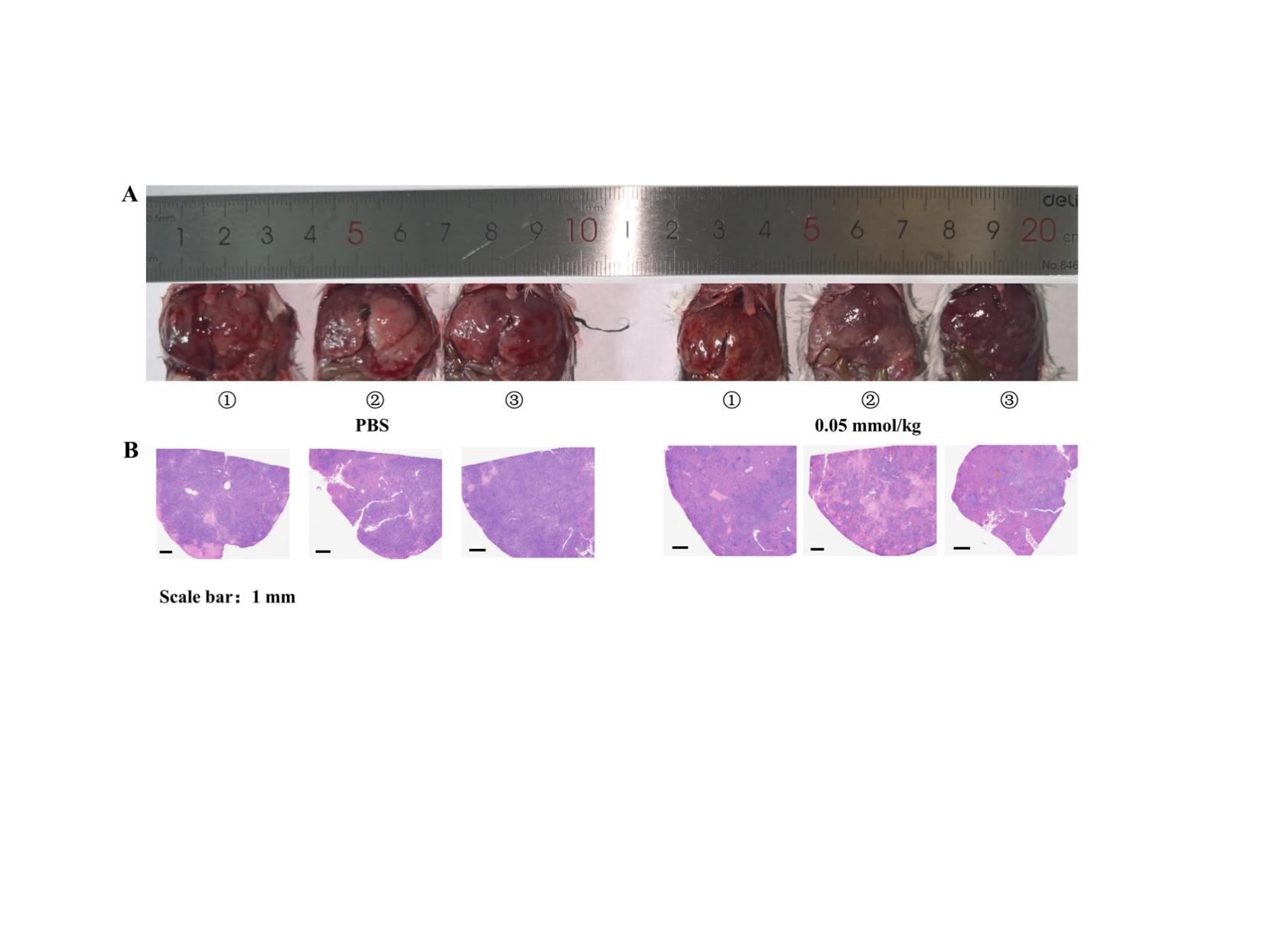


**Figure S22.** RM NPs improved prognostic outcomes in liver metastasis. (**A**) Post-treatment liver dissection images (each group n = 3), (**B**) Corresponding H&E-stained liver sections (Scale bar for all images = 1 mm).


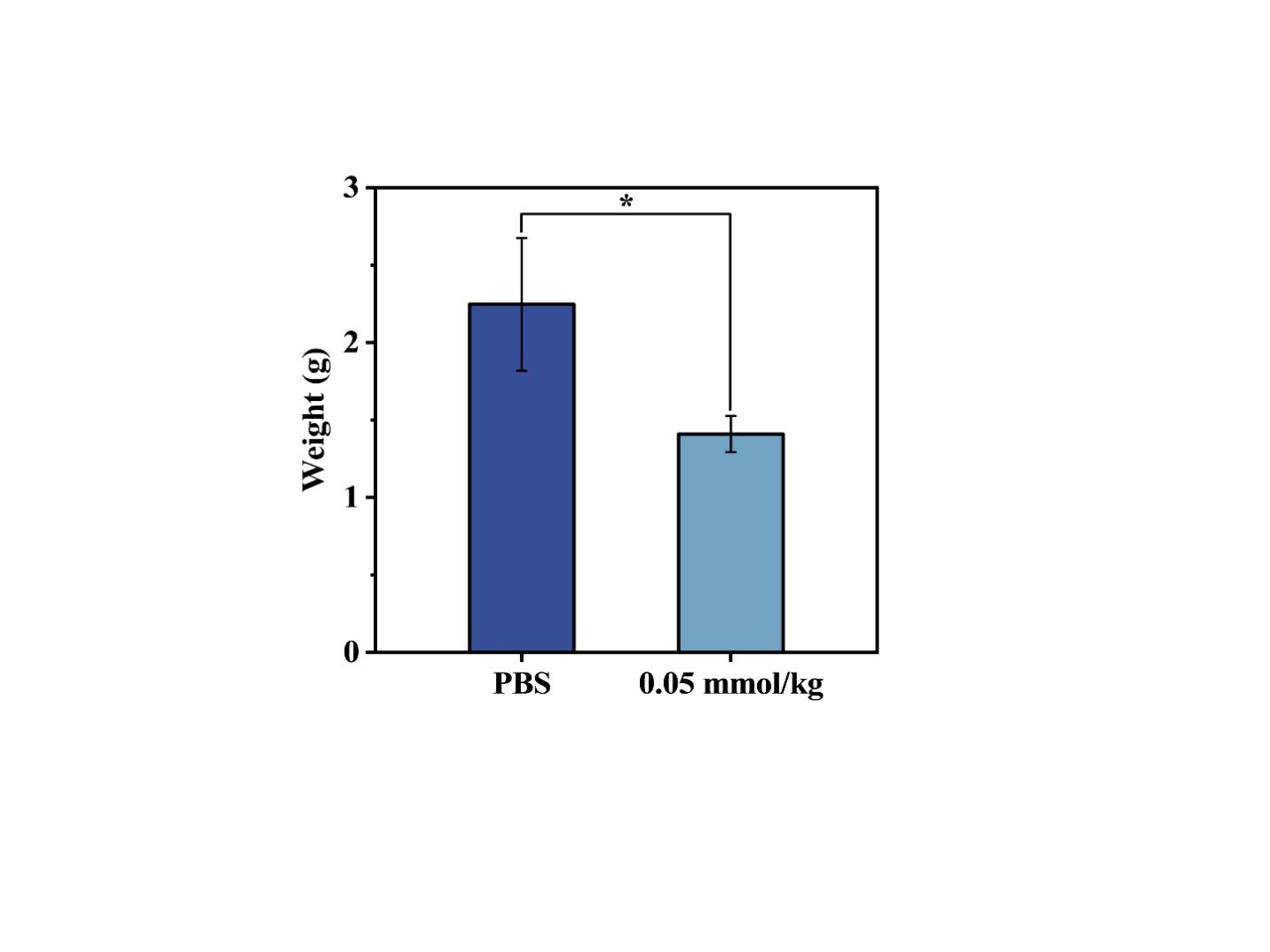


**Figure S23.** Liver weight of mice in the control group and treatment group (mean ± SD, n = 3).


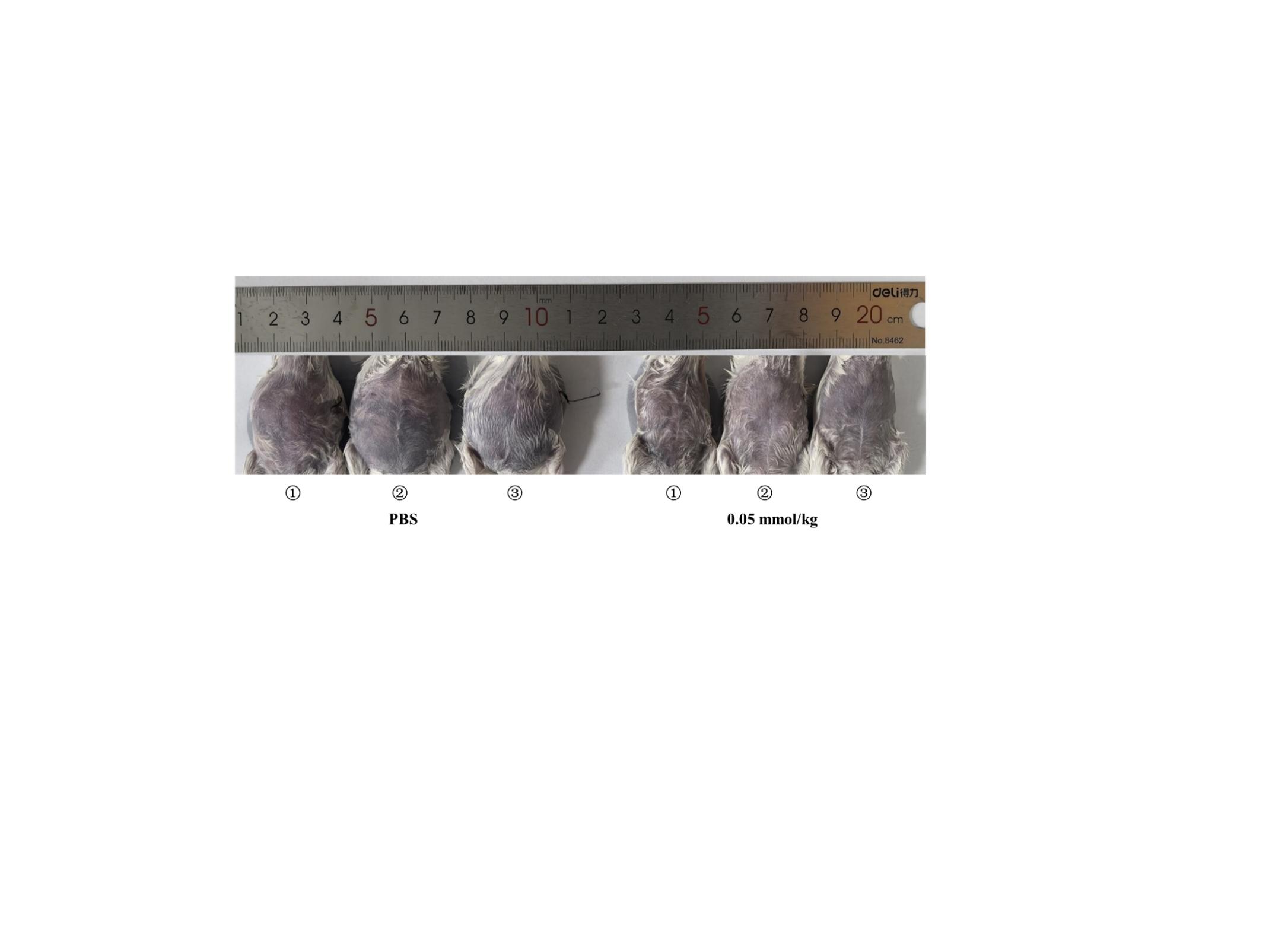


**Figure S24.** Photographs of the abdomen of mice in the control and treatment groups.

**Table S1.** Evaluation of r_1_ and r_2_ relaxivities of three RM NPs with different rutin ligand concentrations, Mn^2+^, and Gd-DTPA at three magnetic fields at room temperature


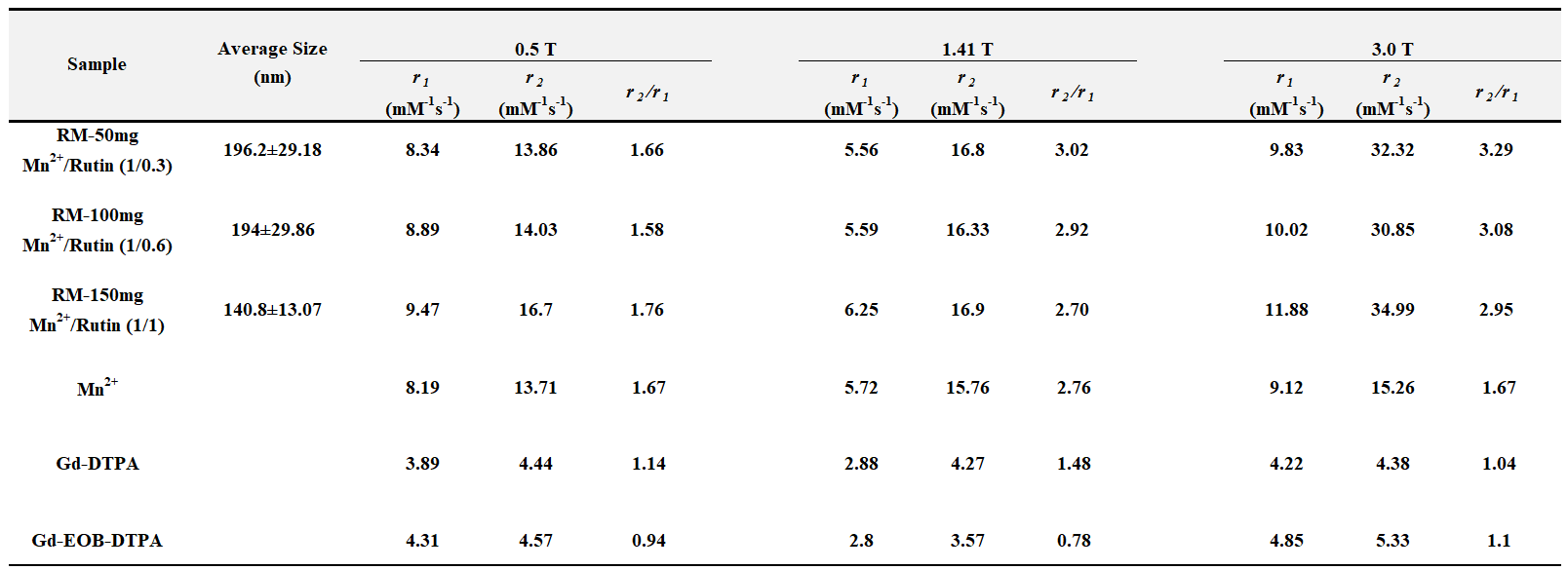

Supplement: rbag049_Supplementary_Data [file rbag049_supplementary_data.docx]
